# Reduction and Rearrangement of a Boron(I) Carbonyl Complex

**DOI:** 10.1002/anie.202014167

**Published:** 2020-12-11

**Authors:** Maximilian Rang, Felipe Fantuzzi, Merle Arrowsmith, Ivo Krummenacher, Eva Beck, Robert Witte, Alexander Matler, Anna Rempel, Tobias Bischof, Krzysztof Radacki, Bernd Engels, Holger Braunschweig

**Affiliations:** ^1^ Institute for Inorganic Chemistry Julius-Maximilians-Universität Würzburg Am Hubland 97074 Würzburg Germany; ^2^ Institute for Sustainable Chemistry & Catalysis with Boron Julius-Maximilians-Universität Würzburg Am Hubland 97074 Würzburg Germany; ^3^ Institute for Physical and Theoretical Chemistry Julius-Maximilians-Universität Würzburg Emil-Fischer-Straße 42 97074 Würzburg Germany

**Keywords:** Biradical, Boron carbonyl complex, density functional calculations, Rearrangement, Reduction

## Abstract

The one‐electron reduction of a cyclic (alkyl)(amino)carbene (CAAC)‐stabilized arylborylene carbonyl complex yields a dimeric borylketyl radical anion, resulting from an intramolecular aryl migration to the CO carbon atom. Computational analyses support the existence of a [(CAAC)B(CO)Ar]^.−^ radical anion intermediate. Further reduction leads to a highly nucleophilic dianionic (boraneylidene)methanolate.

## Graphical abstract

The one‐electron reduction of a cyclic (alkyl)(amino)carbene (CAAC)‐stabilized borylene carbonyl complex yields a dimeric borylketyl radical anion, resulting from an intramolecular aryl migration to the CO carbon atom. Computational analyses support the existence of a [(CAAC)B(CO)Ar]^.−^ radical anion intermediate. Further reduction leads to a highly nucleophilic dianionic (boraneylidene)methanolate.

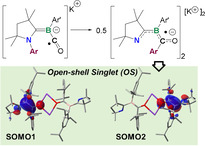

The two‐electron reduction of transition metal (TM) carbonyl complexes, [TM(CO)_*n*_], generally proceeds with loss of CO to the corresponding [TM(CO)_*n*−1_]^2−^ dianions (Figure 1 a), and/or anionic metal carbonyl clusters.[^1^, ^2^, ^3^, ^4^] Studied extensively throughout the last century, [TM(CO)_*n*−1_]^2−^ dianions are highly air‐sensitive nucleophiles[^5^, ^6^] with a rich reactivity toward organometallic, inorganic, and organic compounds.[^7^, ^8^, ^9^, ^10^, ^11^, ^12^] Our group and others have used [TM(CO)_*n*−1_]^2−^ precursors (TM=Cr, Mo, W, *n*=6; M=Fe, *n*=5) for the synthesis of terminal borylene complexes of the form [(OC)_*n*_TM=BR] (R=anionic substituent)[^13^, ^14^, ^15^, ^16^, ^17^] in which the BR ligand is isolobal with CO. The two‐electron reduction of aryl‐ and aminoborylene complexes of this type proceeds quite distinctly, however, from that of [TM(CO)_*n*_], resulting in double B−CO coupling and the release of an iminoborane dimer, respectively (Figure 1 b).^[18]^

**Figure 1:**
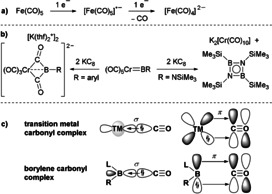
a) One‐ and two‐electron reduction of a TM carbonyl. b) Reduction of TM borylene complexes. c) Analogous orbital interactions between CO and TM or borylene fragments, respectively.

Owing to the lone pair and empty p orbital at boron, metal‐free borylenes of the form L_*n*_BR (L=Lewis base; *n*=1, 2) display reactivity reminiscent of low‐oxidation‐state TM complexes,[^19^, ^20^] including the coordination of CO.[^21^, ^22^, ^23^, ^24^, ^25^, ^26^, ^27^] Borylene carbonyl complexes (LB(CO)R) are generally obtained either by the direct addition of CO to a dicoordinate borylene (LBR)^[27]^ or by releasing TM‐bound borylenes through the addition of CO or other strong donors.[^25^, ^26^] Spectroscopic and theoretical studies show a B−CO bonding pattern analogous to that of TM carbonyls, with the CO ligand donating into the empty orbital at boron and the boron lone pair backdonating into the π* orbital at CO (Figure 1 c).[^24^, ^26^]

Like their TM counterparts, borylene carbonyls undergo exchange reactions with other Lewis bases upon UV irradiation,^[25]^ or photolytic intramolecular oxidative addition with C−H and C−C σ bonds.[^25^, ^26^] Lin and Xie also reported a cationic borylene carbonyl reacting with nucleophiles under reduction, migration, or complete cleavage of CO in a TM‐like manner.^[28]^ Inspired by this metallomimetic behavior, we report herein the one‐ and two‐electron reduction chemistry of a carbonyl borylene, LB(CO)R, and highlight how it differs from that of TM carbonyls.

The low‐temperature reduction of boryl radical **1**, [(CAAC)BCl(Tip)]^.^ (CAAC=1‐(2,6‐diisopropylphenyl)‐3,3,5,5‐tetramethyl‐pyrrolidin‐2‐ylidene; Tip=2,4,6‐triisopropylphenyl),^[29]^ with 1.5 equiv KC_8_ under 1.5 atm CO afforded the tricoordinate carbonyl borylene **2** as an orange solid in 65 % yield (Scheme 1 a). This synthetic route has the advantage of circumventing the need for synthesizing an intermediate TM borylene complex. The ^11^B NMR shift of **2** at −15.1 ppm is similar to that of other (CAAC)B(CO)Ar borylenes, as is the IR C−O stretching band at 1945 cm^−1^.[^21^, ^25^] The solid‐state structure of **2** (see Figure S37 in the Supporting Information) shows a trigonal planar borylene (Σ(∡B1) 359.97(10)°) with delocalized π bonding over the entire N1‐C1‐B1‐C36 framework (N1–C1 1.3575(15), C1–B1 1.5047(17), B1–C36 1.4857(18) Å), similar to other (CAAC)B(CO)R borylenes.[^21^, ^22^, ^23^, ^24^, ^25^, ^26^, ^27^]

**Scheme 1:**
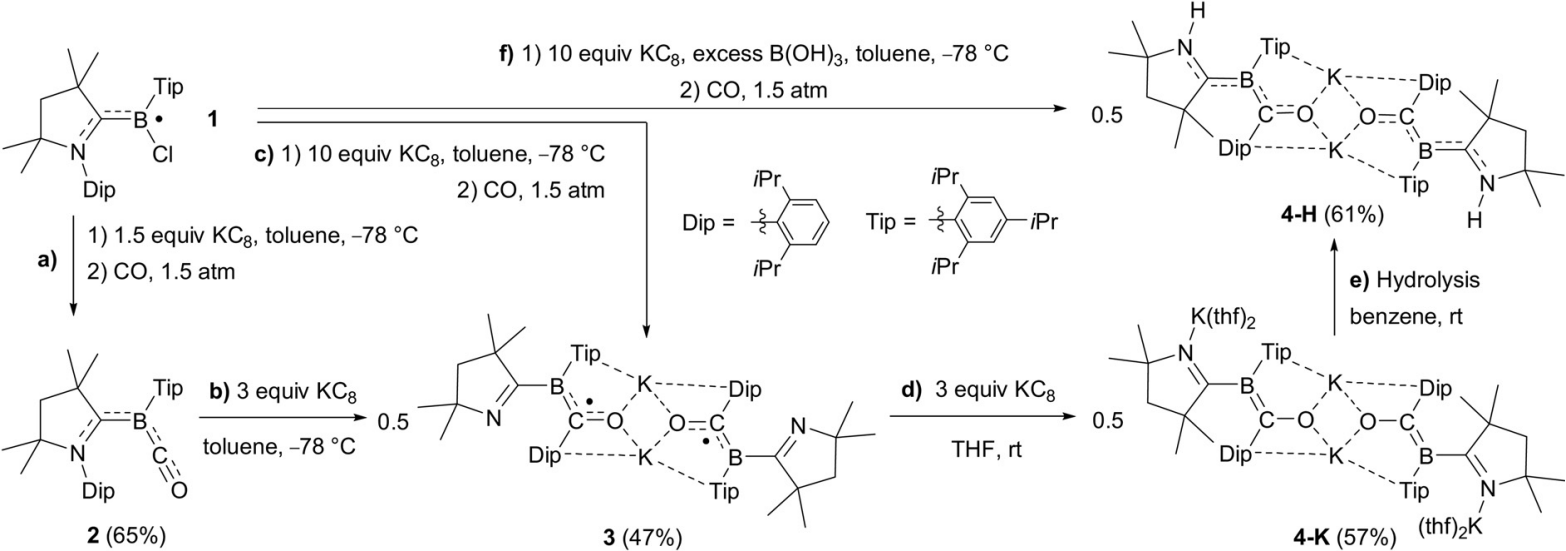
One‐ and two‐electron reduction of borylene carbonyl complex **2**.

The low‐temperature reduction of **2** with 3 equiv KC_8_ (Scheme 1 b) or that of **1** with 10 equiv KC_8_ under 1.5 atm CO (Scheme 1 c) afforded the red, NMR‐silent compound **3**, which shows no identifiable IR C−O stretching bands. X‐ray diffraction analysis of **3** revealed that the Dip substituent has migrated from the CAAC nitrogen to the former CO carbon C36 (Figure 2). The compound dimerizes via O⋅⋅⋅K⋅⋅⋅O bridges, with additional K⋅⋅⋅aryl π interactions. The [C1‐B1(C21)‐C36‐O1‐K1]_2_ framework is quasi‐planar, with delocalized π bonding over the B1‐C36‐O1 unit (B1‐C36 1.527(2); C36‐O1 1.2939(18) Å). Unlike in borylene **2**, the C_4_N rings of the former CAAC ligands are rotated ca. 33° out of the [C1‐B1(C21)‐C36‐O1‐K1]_2_ plane. The short N1–C1 (1.283(2) Å) and significantly lengthened B1–C1 distances (1.577(2) Å) indicate a localized C=N double and B−C single bond, respectively, turning the ligand into an anionic 3,4‐dihydro‐2*H*‐pyrrol‐5‐yl. These structural features, combined with the EPR‐active nature of **3** allow its identification as a dimeric borylketyl radical anion, the first of its kind.

**Figure 2:**
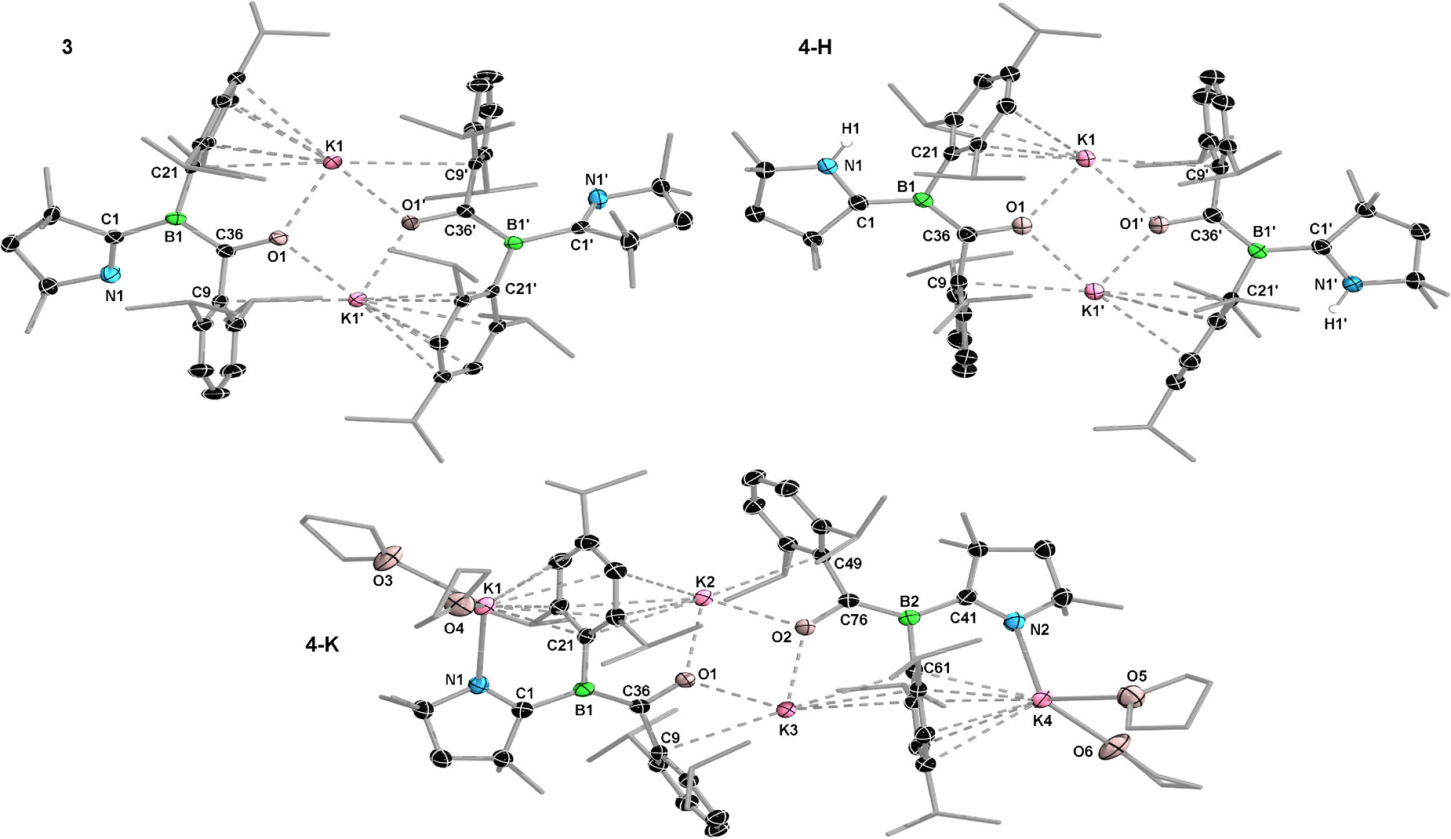
Crystallographically‐derived molecular structures of **3**, **4‐H**, and **4‐K**.^[54]^ Atomic displacement ellipsoids set at 50 % probability. Ellipsoids of ligand periphery and hydrogen atoms omitted for clarity, except for H1 in **4‐H** (detected in the difference Fourier map and freely refined). Selected bond lengths [Å] and angles [°]) for **3** (Et_2_O co‐crystal): N1–C1 1.283(2), C1–B1 1.577(2), B1–C36 1.527(2), C36–O1 1.2939(18), O1⋅⋅⋅K1 2.5963(13), O1⋅⋅⋅K1′ 2.6488(13), torsion (N1,C1,B1,C36) 33.1(2); for **4‐H**: N1–C1 1.377(2), C1–B1 1.503(2), B1–C36 1.518(2), C36–O1 1.301(2), O1⋅⋅⋅K1 2.5513(13), O1⋅⋅⋅K1′ 2.7040(13), torsion (N1,C1,B1,C21) −2.8(3); for **4‐K**: K1–N1 2.6759(18), N1–C1 1.326(3), C1–B1 1.557(3), B1–C36 1.484(3), C36–O1 1.343(2), O1⋅⋅⋅K2 2.5282(14), O1⋅⋅⋅K3 2.5767(13), torsion (N1,C1,B1,C21) −0.8(3).

The solution EPR spectrum of **3** in a toluene/THF mixture at 270 K displays a six‐line resonance (Figure 3 a), for which simulation provides the following hyperfine coupling constants: *a*(^11^B)=11.7 MHz (4.2 G) and *a*(^14^N)=9.8 MHz (3.5 G). Given the absence of exchange coupling effects we assume that **3** exists as a monomer in solution. In the solid state, **3** exhibits featureless broad EPR spectral signals (Figure 3 b). While again no direct evidence for electron–electron exchange coupling was found, fitting of the temperature‐dependent double‐integral intensity to the Bleaney–Bowers model (Figure 3 c,d) suggests a weak electron exchange interaction with 2*J*=−12 cm^−1^, that is, a small singlet–triplet gap of Δ*E*_ST_=0.14 kJ mol^−1^. This result is consistent with **3** existing as a ground‐state singlet with a thermally accessible triplet state. These findings agree well with our computations using density functional theory (DFT) and high‐level multireference approaches based on the complete active space self‐consistent field (CASSCF)^[30]^ and the N‐electron valence state second‐order perturbation theory (NEVPT2)[^31^, ^32^, ^33^] methods (Figure 3 e). At the B3LYP[^34^, ^35^, ^36^, ^37^]‐D3^[38]^(BJ)^[39]^/def2‐SVP^[40]^ level, the closed‐shell singlet state (CS) of **3** has a very small HOMO–LUMO gap of 0.52 eV, with these orbitals being, respectively, composed of +,+ and +,− combinations of fragment orbitals located mainly at the π space of the BCO moieties. Unrestricted DFT reveals an open‐shell singlet state (OS), which is 87.0 kJ mol^−1^ more stable than the CS solution. Both DFT (0.08 kJ mol^−1^) and NEVPT2 (0.13 kJ mol^−1^) show that this state is slightly more favored than the triplet state, as deduced from the EPR data. The preference for the singlet indicates that the spin centers have a small but non‐negligible interaction,^[41]^ which breaks the orbital degeneracy and leads to a small energy gap between the frontier CASSCF orbitals (see SI for more details).

**Figure 3:**
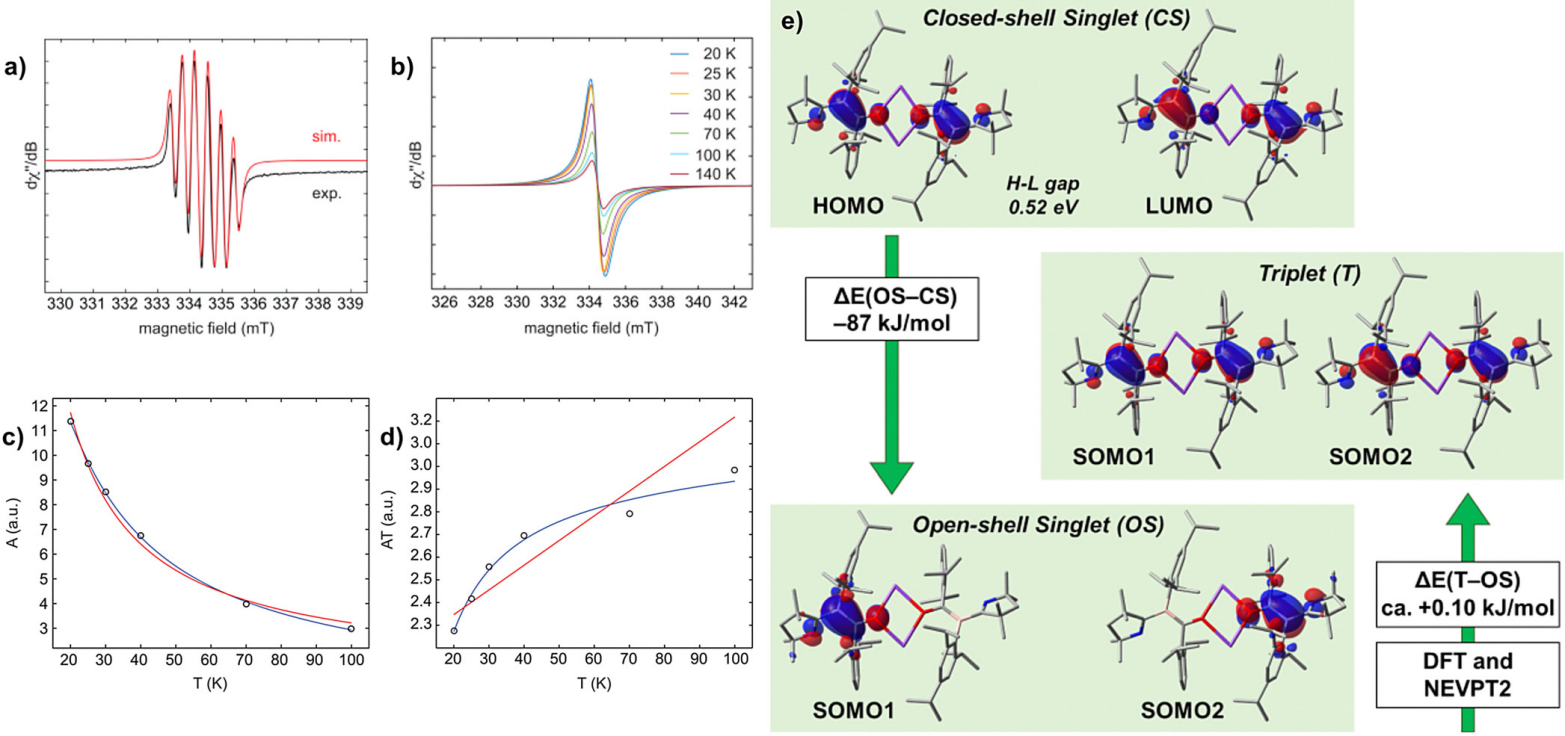
a) Experimental (black) and simulated (red) continuous‐wave X‐band EPR spectra of **3** in a toluene/thf mixture at 270 K. Simulation parameters: *g*_iso_=2.0040, *a*(^11^B)=11.7 MHz (4.2 G), and *a*(^14^N)=9.8 MHz (3.5 G). b) Variable‐temperature X‐band EPR spectra of solid **3** diluted in KBr between 20 and 140 K. The EPR spectra recorded for neat powders are essentially identical. c),d) Temperature dependence of the double integral EPR intensity (*A*) of **3**. Circles (○) represent the experimental results, the blue line the fit with the Bleaney–Bowers equation and the red line represents Curie behavior. e) Selected frontier molecular orbitals of **3** in its CS singlet, triplet, and OS singlet states, and relevant energy differences (kJ mol^−1^) calculated at DFT and NEVPT2.

The possible resonance forms of monomeric **[3]^.−^**, which help stabilize the radical and anionic charge, are shown in Figure 4, together with the Mulliken spin densities of dimeric **3** and natural bond order (NBO)^[42]^ calculations within the “different hybrids for different spins” approach.^[43]^ The spin densities are delocalized throughout the BCO moieties, with the largest contribution at boron, in agreement with the experimental EPR hyperfine coupling constants. The NBO picture of the system indicates a bonding situation resembling the mesomeric structures **B** (α system) and **D** (β system). The dominant attractive contribution to the structure comes from the O(lp)→B‐C(π*) donor–acceptor interaction (α system), as revealed by the second‐order stabilization energies (Figure 4 c). This also indicates that delocalization through BCO plays a major role for the stabilization of **3**.

**Figure 4:**
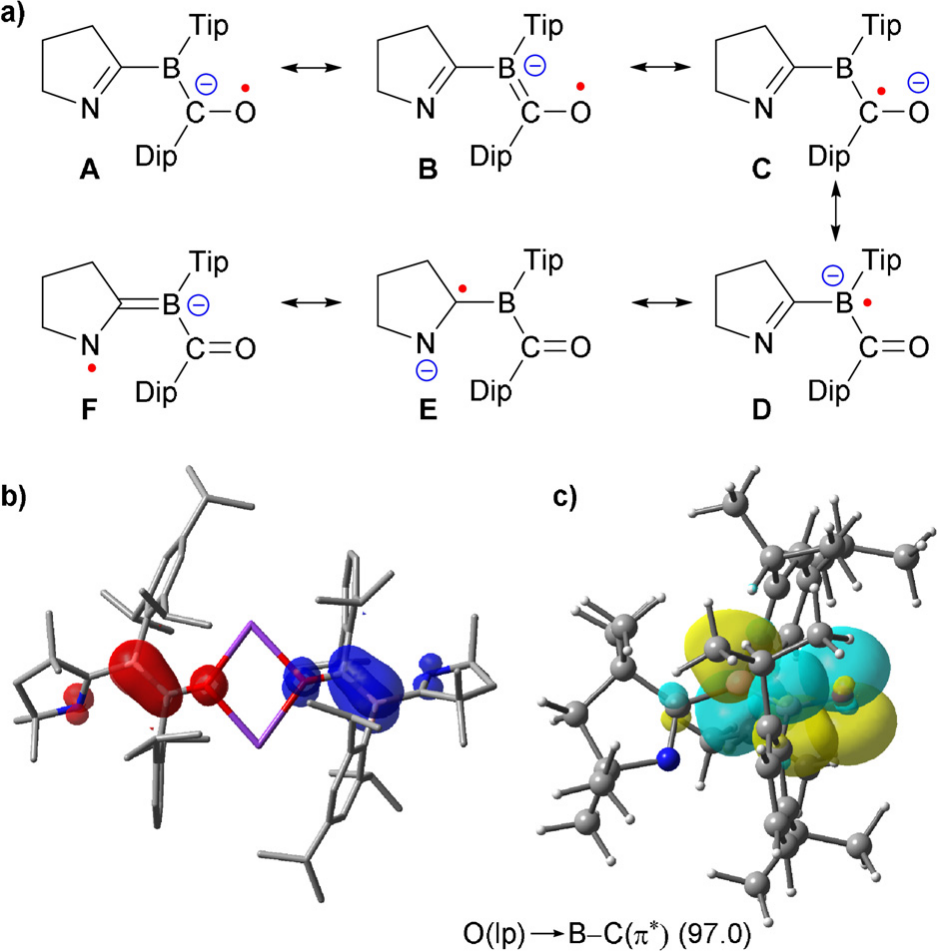
a) Mesomeric resonance forms of monomeric **[3]^.−^** (methyl groups truncated for clarity). b) Plot of Mulliken spin density of **3** (B3LYP‐D3(BJ)/def2‐SVP). Spin density distribution: N1 −0.09, C1+0.07, B1 −0.50, C36 −0.26, O1 −0.23. c) Leading NBO donor–acceptor interaction (α system) of **[3]^.−^** (O(lp)→B‐C(π*), 97.0 kJ mol^−1^).

The unexpected formation of **3** from the reduction of **2** can be rationalized by the one‐electron reduction of **2** to an intermediate borylene radical anion **[2]^.−^**, followed by radical attack of the CO carbon C36 at the *ipso*‐carbon of the Dip group, and subsequent migration of Dip to C36 to generate **[3]^.−^** (Scheme 2). DFT calculations revealed that **[2]^.−^** is indeed a stable minimum energy structure with a quasi‐linear BCO arrangement of 174.4°, a B1−C1 bond length of 1.507 Å, and spin density mainly located at C1 (0.64) and C36 (0.38, Scheme 2). Radical anions of TM carbonyls and their clusters can be generated both chemically and electrochemically.[^44^, ^45^, ^46^, ^47^] While a DFT study of group‐6 [TM(CO)_4_PPh_3_]^.−^ radical anions showed that spin density in these species is largely located at the metal center,^[48]^ [TM(CO)_*n*_]^.−^ complexes (TM=Fe, *n*=5; TM=Cr, *n*=6) also display CO‐centered radical reactivity similar to that of **[2]^.−^**, undergoing facile hydrogen atom transfer with trialkyltin hydrides to yield the formyl complexes [TM(CO)_*n*−1_(CHO)]^−^.^[49]^ To our knowledge the radical transfer of a nitrogen‐bound aryl group to CO has never been observed in TM carbonyl chemistry. In low‐valent main group chemistry, however, the cleavage of N−C_aryl_ bonds by the insertion of borylene or silylene fragments has been observed at N‐heterocyclic olefin^[50]^ or carbene ligands,^[51]^ respectively.

**Scheme 2:**
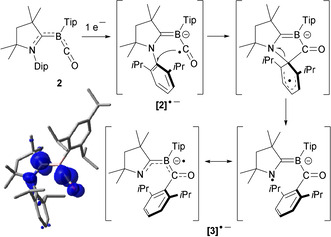
Proposed mechanism for the formation of **3** from intermediate **[2]^.−^** and plot of Mulliken spin density of **[2]^.−^** (UB3LYP‐D3(BJ)/6‐31++G**). Spin density distribution: N1+0.03, C1+0.64, B1 −0.09, C36+0.38, O1+0.10.

The cyclic voltammogram of **3** in THF showed several irreversible oxidation waves as well as a reduction wave at −2.66 V (relative to the Fc/Fc^+^ couple), suggesting the possibility of further chemical reduction (see Figure S36 in the SI). Indeed the reduction of **3** with 3 equiv KC_8_ in THF yielded a dark red solution of an extremely air‐sensitive NMR‐active species, compound **4‐K** (Scheme 1 d), with an ^11^B NMR shift at 14.5 ppm. Upon hydrolysis of **4‐K** the N‐protonated dimer **4‐H** was isolated (Scheme 1 e), with a similar ^11^B NMR shift at 15.0 ppm and a characteristic ^1^H NMR N*H* singlet at 3.82 ppm. Alternatively, **4‐H** could be accessed directly from **1** by reduction with 10 equiv KC_8_ under CO in the presence of B(OH)_3_ as the proton source (Scheme 1 f).

At first sight the solid‐state structure of **4‐H** is very similar to that of **3** (Figure 2), displaying a dimeric structure with central O⋅⋅⋅K⋅⋅⋅O bridges and additional K⋅⋅⋅aryl π interactions. Upon closer inspection, however, there are significant differences. The C_4_N ring of the former CAAC ligand, which has flipped around the B1−C1 bond and now bears a nitrogen‐bound hydrogen atom (located in the difference Fourier map), is now aligned with the boron plane. This results in extensive π delocalization over the entire quasi‐planar [N1‐C1‐B1(C21)‐C36‐O1‐K1]_2_ framework (N1‐C1 1.377(2), C1‐B1 1.503(2), B1‐C36 1.518(2), C36‐O1 1.301(2) Å), indicating that the protonated C_4_N ring now acts as a π‐accepting CAAC ligand again. **4‐H** may therefore be described as a CAAC‐stabilized aryl(benzoyl)boryl anion or an aryl(boraneylidene)methanolate. The solid‐state structure of **4‐K** (Figure 2) shows two additional potassium cations bound to N1 and N2 and stabilized by two THF molecules each, as well as η^6^‐K⋅⋅⋅Tip π interactions. Compared to the corresponding bonds of **4‐H** the N1/2‐C1/41 and B1/2‐C36/76 bonds are shortened by 0.03–0.05 Å, while the C1/41‐B1/2 and C36/76‐O1/2 bonds are lengthened by 0.04–0.05 Å. Accordingly, the calculated Mayer bond orders[^52^, ^53^] of N1/2‐C1/41 and B1/2‐C36/76 are increased, respectively by 28 % and 16 %, while those of the C1/41‐B1/2 and C36/76‐O1/2 bonds are decreased by ca. 12 %, upon going from **4‐H** to **4‐K**. These results indicate that **4‐K** is better described as an aryl(boraneylidene)methanolate, with its π electron density essentially localized on the N1−C1 and B1−C36 bonds.

The reactions of **4‐H** with various electrophiles, including B(OH)_3_, MeOTf (Tf=triflate) and Me_3_SiCl, resulted in exclusive functionalization of the oxygen atom (Scheme 3), thereby confirming that **4‐H** behaves more like a methanolate species than a boryl anion. The resulting products, **5‐H**, **5‐Me**, and **5‐TMS**, show ^11^B NMR resonances in the 15 to 18 ppm region. In the solid state (see Figures S38–S40 in the SI), all three compounds show the N‐protonated CAAC ligand acting as a pure σ donor (C1–B1 ca. 1.55 Å) and the B1=C36 bond displays double bond character (ca. 1.48 Å).

**Scheme 3:**
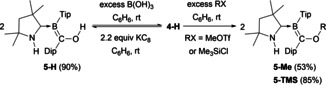
Reactions of **4‐H** with electrophiles.

To conclude, despite analogies in their bonding patterns the reduction of a (CAAC)B(CO)Ar borylene carbonyl complex proceeds quite differently from that of TM carbonyls. While the latter may undergo a one‐electron reduction to a metal‐centered radical anion, the one‐electron reduction of (CAAC)B(CO)Ar results in an unprecedented aryl migration from the CAAC nitrogen to the former carbonyl carbon atom, yielding a novel ketyl boron radical anion. Calculations show that this reaction likely proceeds via an intermediate [(CAAC)B(CO)Ar]^.−^ radical anion, with a significant amount of spin density localized at the carbonyl carbon rather than the boron atom. This work highlights once more that borylenes display a unique reactivity quite distinct from TM analogues.

## Conflict of interest

The authors declare no conflict of interest.

## Supporting information

As a service to our authors and readers, this journal provides supporting information supplied by the authors. Such materials are peer reviewed and may be re‐organized for online delivery, but are not copy‐edited or typeset. Technical support issues arising from supporting information (other than missing files) should be addressed to the authors.

Supplementary
