# Supplementary material for: Reduction and Rearrangement of a Boron(I) Carbonyl Complex
Source: Angew Chem Int Ed Engl. 2020 Dec 11;60(6):2963–8. doi: 10.1002/anie.202014167 (PMC7898892; doi:10.1002/anie.202014167)
Supplement: Supplementary file 1 — Supplementary [file ANIE-60-2963-s001.pdf]

Angewandte Chemie
Eine Zeitschrift der Gesellschaft Deutscher Chemiker

Supporting Information

Reduction and Rearrangement of a Boron(I) Carbonyl Complex

*Maximilian Rang, Felipe Fantuzzi, Merle Arrowsmith, Ivo Krummenacher, Eva Beck, Robert Witte, Alexander Matler, Anna Rempel, Tobias Bischof, Krzysztof Radacki, Bernd Engels, and Holger Braunschweig**

anie_202014167_sm_miscellaneous_information.pdf

Supporting Information

Table of Contents

Methods and materials 2

Synthetic procedures 3

NMR spectra of isolated compounds 10

EPR-spectroscopic study of **3** 28

UV-vis spectra 30

IR spectra 34

Cyclic voltammetry 41

X-ray crystallographic data 42

Computational details 48

Cartesian coordinates 62

References 112

Methods and materials

All manipulations were performed either under an atmosphere of dry argon or *in vacuo* using standard Schlenk line or glovebox techniques. Deuterated solvents were dried over molecular sieves and degassed by three freeze-pump-thaw cycles prior to use. All other solvents were distilled and degassed from appropiate drying agents. Solvents were stored under argon over activated 4 Å molecular sieves. NMR spectra were acquired on a Bruker Avance 400 or 500 NMR spectrometer at 298 K, unless indicated otherwise. Chemical shifts (*δ*) are provided in ppm and internally referenced to the carbon nuclei ($^{13}C\{^{1}H\}$) or residual protons (^{1}H) of the solvent. Heteronuclei NMR spectra are referenced to external standards (^{11}B: $BF_3{\cdot}OEt_2$). UV-vis spectra were acquired on a JASCO-V660 UV-vis spectrometer. Solid-state IR spectra were recorded on a Jasco FT/IR-6200 spectrometer inside a glovebox. High-resolution mass spectrometry data were obtained from a Thermo Scientific Exactive Plus spectrometer in LIFDI or ASAP mode.

Reagents were purchased from Sigma Aldrich or Alfa Aesar and used as received. The boryl radical **1**[1] was prepared following a literature procedure. Potassium graphite (KC_8) was prepared by heating graphite (20.36 g, 1.695 mol; first dried in a 100 °C oven overnight) and freshly-cut potassium (8.35 g, 214 mmol) at 250 °C under vigorous stirring and a flow of argon for 2 hours.

Synthetic procedures

Synthesis of compound 2

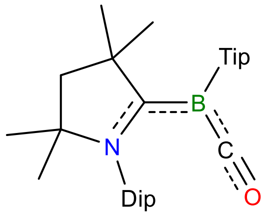

A mixture of **1** (400 mg, 748 µmol) and KC_8 (152 mg, 1.12 mmol) was cooled to −78 °C and toluene (30 mL) was added slowly. The reaction mixture was frozen using liquid nitrogen, degassed, then allowed to warm to the point of thawing, whereupon CO (1.5 atm) was introduced into the flask. The reaction mixture was stirred vigorously for 2 h at room temperature, filtered and the filtrate dried *in vacuo*. The resulting orange solid was recrystallized from hexanes (2 mL) at −30 °C to afford **2** (250 mg, 483 µmol, 65% yield). Suitable crystals for X-ray diffraction analysis were obtained by slow evaporation of a saturated pentane solution at −30 °C. ^{1}H NMR (500.1 MHz, C_6D_6): δ = 7.28 (dd, ^3J = 8.1 Hz, ^3J = 7.3 Hz, 1H, *p*-CH^{Dip}), 7.24 (s, 2H, *m*-CH^{Tip}), 7.18 (dd, ^3J = 7.5 Hz, ^4J = 0.5 Hz, 2H, *m*-CH^{Dip}), 3.80 (sept, ^3J = 6.9 Hz, 2H, C$H(CH_3)_2^{Tip}$), 3.09 (sept, ^3J = 6.7 Hz, 2H, C$H(CH_3)_2^{Dip}$), 2.92 (sept, ^3J = 6.9 Hz, 1H, C$H(CH_3)_2^{Tip}$), 1.63 (s, 2H, CH_2^{cAAC}), 1.51 (d, ^3J = 6.7 Hz, 6H, CH(C$H_3)_2^{Dip}$), 1.51 (d, ^3J = 6.9 Hz, 6H, CH(C$H_3)_2^{Tip}$), 1.33 (d, ^3J = 6.8 Hz, 6H, CH(C$H_3)_2^{Tip}$), 1.22 (d, ^3J = 6.8 Hz, 6H, CH(C$H_3)_2^{Dip}$), 1.18 (d, 6H, CH(C$H_3)_2^{Tip}$), 1.17 (s, 6H, CH_3^{cAAC}), 1.05 (s, 6H, CH_3^{cAAC}) ppm. ^{13}C{^{1}H} NMR (125.8 MHz, C_6D_6): δ = 205.0 ($C_{Carbene}$, identified by HMBC), 154.6 (*o*-C_q^{Tip}), 149.6 (*o*-C_q^{Dip}), 148.6 (*p*-C_q^{Tip}), 134.7 (*i*-C_q^{Dip}), 133.0 (*i*-C_q^{Tip}, identified by HMBC), 130.3 (*p*-CH^{Dip}), 126.0 (*m*-CH^{Dip}), 120.5 (*m*-CH^{Tip}), 68.6 (C_q^{cAAC}), 55.2 (CH_2^{cAAC}), 48.6 (C_q^{cAAC}), 35.4 (CH(CH$_3)_2^{Tip}$), 34.9 (CH(CH$_3)_2^{Tip}$), 30.4 (CH_3^{cAAC}), 30.2 (CH_3^{cAAC}), 28.8 (CH(CH$_3)_2^{Dip}$), 28.0 (CH(CH$_3)_2^{Tip}$), 26.9 (CH(CH$_3)_2^{Dip}$), 25.3 (CH(CH$_3)_2^{Dip}$), 24.5 (CH(CH$_3)_2^{Tip}$), 22.7 (CH(CH$_3)_2^{Tip}$) ppm. *Note: The resonance of the carbonyl carbon nucleus was not detected due to quadrupolar line broadening; the coupling constant of the doublet at 1.18 ppm cannot be determined due to overlapping with the singlet at 1.17.* ^{11}B NMR (160.5 MHz): δ = −15.1 (s) ppm. UV-vis (THF): λ_{max} = 388 nm. IR (solid state): ṽ(CO) = 1945 cm^{-1}. HRMS (LIFDI) calculated for [$C_{36}H_{54}BNO$] = [M]: 527.4293; found: 527.4282.

Synthesis of compound 3

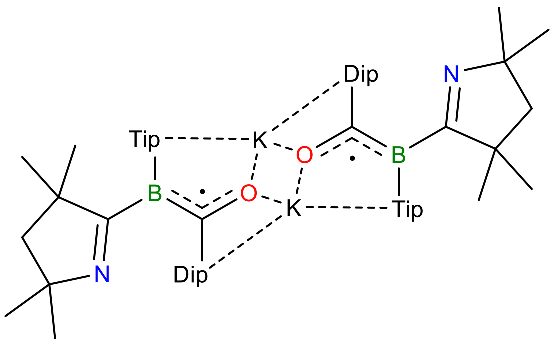

A mixture of **1** (300 mg, 561 μmol) and KC_8 (758 mg, 5.61 mmol) was cooled to −78 °C and toluene (30 mL) was added slowly. The reaction mixture was frozen using liquid nitrogen, degassed, then allowed to warm to the point of thawing, whereupon CO (1.5 atm) was introduced into the flask. The reaction mixture was stirred vigorously for 4 h at room temperature, filtered and the filtrate dried *in vacuo*. The crude yield of compound **3** amounted to 71% (225 mg, 199 μmol). Further purification was achieved through crystallization by slow evaporation of a benzene solution at room temperature, washing with diethyl ether and recrystallization of the ether solution at −30 °C (150 mg, 132 μmol, 47%). Suitable crystals for X-ray diffraction analysis were obtained by slow evaporation of a saturated diethyl ether solution at −30 °C. UV-vis (THF): λ_{max} = 443 nm. HRMS (LIFDI) calculated for [$C_{36}H_{56}BNO$] = [M – K + 2H]: 529.4449; found: 529.4442.

Synthesis of compound 4-K

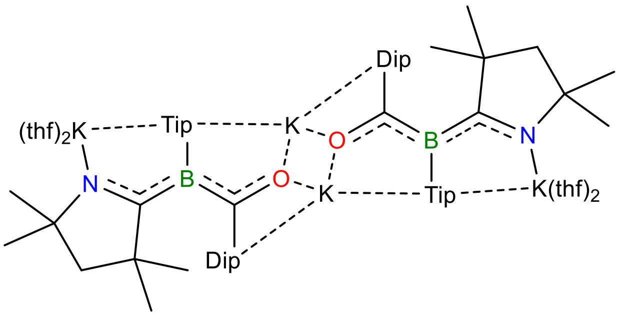

To a mixture of **3** (20.0 mg, 17.6 μmol) and KC_8 (7.20 mg, 52.9 μmol) 0.8 mL of THF were added. The suspension was stirred vigorously until it turned dark, filtered and subsequently layered with hexanes (1.5 mL). After storing the solution at −30 °C for two days compound **4-K** was isolated as dark red crystals suitable for X-ray diffraction analysis. After decanting the mother liquor, the vial containing the crystals was left standing open in the freezer to allow the

remaining solvent to slowly evaporate, yielding **4-K** in 57% yield (15.0 mg, 10.0 µmol). Vacuum drying of **4-K** only resulted in hydrolysis to **4-H**. ^{1}H NMR (500.1 MHz, d$_8$-thf): δ = 6.80 – 6.76 (m, 10H, *m*-CH^{Dip}, *p*-CH^{Dip}, *m*-CH^{Tip}), 4.40 (sept, ^{3}J = 6.7 Hz, 4H, CH(CH$_3$)$_2^{Dip}$), 4.34 (sept, ^{3}J = 6.7 Hz, 4H, CH(CH$_3$)$_2^{Tip}$), 2.80 (sept, ^{3}J = 6.8 Hz, 2H, CH(CH$_3$)$_2^{Tip}$), 1.25 (s, 2H, CH_2^{cAAC}), 1.25 (d, ^{3}J = 6.7 Hz, 12H, CH(CH_3)$_2^{Tip}$), 1.12 (d, ^{3}J = 6.9 Hz, 12H, CH(CH_3)$_2^{Tip}$), 1.09 (d, ^{3}J = 6.8 Hz, 12H, CH(CH_3)$_2^{Dip}$), 1.00 (d, ^{3}J = 6.8 Hz, 12H, CH(CH_3)$_2^{Tip}$), 0.97 (d, ^{3}J = 6.7 Hz, 12H, CH(CH_3)$_2^{Dip}$), 0.91 (s, 12H, CH_3^{cAAC}) , 0.88 (s, 12H, CH_3^{cAAC}) ppm. *Note: The resonances of the CH$_2^{cAAC}$ and iPr^{Tipp} groups overlap.* ^{13}C{^{1}H} NMR (125.8 MHz, d$_8$-thf): δ = 200.6 ($C_{Carbene}$, identified by HMBC), 168.9 ($C_q^{Alkylidene}$), 158.8 (*i*-C_q^{Dip}), 154.9 (*o*-C_q^{Tip}), 146.0 (*o*-C_q^{Dip}), 143.5 (*p*-C_q^{Tip}), 123.3 (*p*-CH^{Dip}), 121.9 (*m*-CH^{Dip}), 118.1 (*m*-CH^{Tip}), 67.7 (C_q^{cAAC}), 58.0 (CH$_2^{cAAC}$), 53.8 (C_q^{cAAC}), 34.8 (CH(CH$_3$)$_2^{Tip}$), 33.2 (CH$_3^{cAAC}$), 33.0 (CH(CH$_3$)$_2^{Tip}$), 31.8 (CH$_3^{cAAC}$), 30.1 (CH(CH$_3$)$_2^{Dip}$), 29.1 (CH(CH$_3$)$_2^{Dip}$), 25.3 (CH(CH$_3$)$_2^{Tip}$), 25.2 (CH(CH$_3$)$_2^{Tip}$), 25.1 (CH(CH$_3$)$_2^{Tip}$), 23.0 (CH(CH$_3$)$_2^{Dip}$) ppm. *Note: The resonance of the i-C^{Tip} carbon nucleus was not detected due to quadrupolar line broadening.* ^{11}B NMR (160.5 MHz, d$_8$-thf): δ = 14.5 (s) ppm. UV-vis (THF): λ_{max} = 438 nm. IR (solid state): $\tilde{\nu}$(CO) = 2237 cm^{-1}, $\tilde{\nu}$(CO) = 2087 cm^{-1}. *Note: **4-K** proved too sensitive for HRMS or Elemental analysis.*

Synthesis of compound 4-H

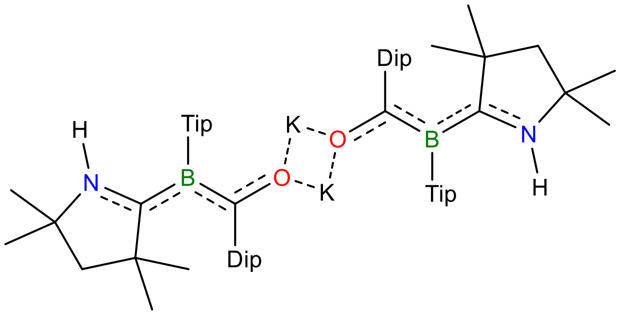

A mixture of **1** (200 mg, 374 µmol), KC$_8$ (505 mg, 3.74 mmol) and B(OH)$_3$ (93.0 mg, 1.50 mmol) was cooled to −78 °C and toluene (20 mL) was added slowly. The reaction mixture was frozen using liquid nitrogen, degassed, then allowed to warm to the point of thawing, whereupon CO (1.5 atm) was introduced into the flask. The reaction mixture was stirred vigorously for 2 h at room temperature, filtered and the filtrate dried *in vacuo*. The resulting orange solid was washed with hexanes (2 mL) and recrystallized at −30 °C to afford a first crop of **4-H** (52.0 mg, 45.8 µmol, 25% yield). The remaining solid was dissolved in benzene (3 mL)

and freeze-dried to obtain an additional crop of **4-H** (58.0 mg, 51.1 μmol, 27% yield). Suitable crystals for X-ray diffraction analysis were obtained by slow evaporation of a saturated pentane solution at −30 °C. *Note: alternatively,* ***4-H*** *can be obtained by a) reducing* ***2*** *with 4 equiv.* KC_8 *and 4 equiv.* $B(OH)_3$ *in toluene at rt (58% yield), b) by treating* ***4-K*** *with 2 equiv.* $B(OH)_3$ *in benzene at rt (NMR shows quantitative conversion), or c) reducing* ***5-H*** *with 2 equiv.* KC_8 *in toluene at rt (61% yield).* ^{1}H NMR (400.1 MHz, C_6D_6): δ = 6.96 (s, 4H, *m*-CH^{Tip}), 4.05 (sept, ^3J = 6.7 Hz, 4H, C$H(CH_3)_2^{Tip}$), 3.82 (s, 2H, NH^{cAAC}), 3.73 (sept, ^3J = 6.9 Hz, 4H, C$H(CH_3)_2^{Dip}$), 2.64 (sept, ^3J = 6.9 Hz, 2H, C$H(CH_3)_2^{Tip}$), 1.45 (s, 4H, CH_2^{cAAC}), 1.43 (d, ^3J = 6.9 Hz, 6H, CH(C$H_3)_2^{Tip}$), 1.41 (d, ^3J = 6.9 Hz, 12H, CH(C$H_3)_2^{Dip}$), 1.17 (m, 12H, CH(C$H_3)_2^{Tip}$), 1.16 (s, 12H, CH_3^{cAAC}), 1.13 (d, ^3J = 6.9 Hz, 12H, CH(C$H_3)_2^{Dip}$), 1.05 (d, ^3J = 6.8 Hz, 12H, CH(C$H_3)_2^{Tip}$), 0.96 (s, 12H, CH_3^{cAAC}) ppm. *Note: The resonances of the aryl-CH protons of the Dip moiety overlap with the resonance of the deuterated solvent at 7.16 ppm.* ^{13}C{^{1}H} NMR (125.8 MHz, C_6D_6): δ = 177.2 ($C_{Carbene}$, identified by HMBC), 154.0 (*o*-C_q^{Tip}), 152.8 (*i*-C_q^{Tip}, identified by HMBC), 151.4 (*i*-C_q^{Dip}), 145.7 (*p*-C_q^{Tip}), 144.4 (*o*-C_q^{Dip}), 126.5 (*p*-CH^{Dip}), 123.7 (*m*-CH^{Dip}), 118.8 (*m*-CH^{Tip}), 58.2 (C_q^{cAAC}), 54.5 (CH_2^{cAAC}), 47.9 (C_q^{cAAC}), 34.2 ($CH(CH_3)_2^{Tip}$), 34.0 ($CH(CH_3)_2^{Tip}$), 31.3 (CH_3^{cAAC}), 30.8 (CH_3^{cAAC}), 28.8 ($CH(CH_3)_2^{Dip}$), 27.2 (CH($CH_3)_2^{Tip}$), 25.4 (CH($CH_3)_2^{Dip}$), 24.4 (CH($CH_3)_2^{Dip}$), 24.3 (CH($CH_3)_2^{Tip}$), 22.8 (CH($CH_3)_2^{Tip}$) ppm. *Note: The resonance of the carbonyl carbon nucleus was not detected due to quadrupolar line broadening.* ^{11}B NMR (160.5 MHz): δ = 15.0 (s) ppm. UV-vis (THF): λ_{max} = 441 nm. IR (solid state): ṽ(NH) = 3399 cm^{-1}, ṽ(CO) = 1975 cm^{-1}. HRMS (LIFDI) calculated for [$C_{36}H_{56}BNO$] = [M – K + H]: 529.4449; found: 529.4443.

Synthesis of compound 5-H

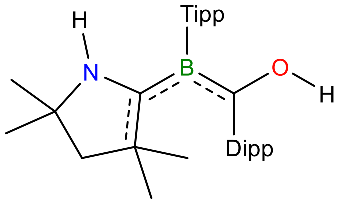

Compound **4-H** (45 mg, 39.6 μmol) was dissolved in benzene (10 mL) and stirred over an excess of $B(OH)_3$ at rt for 2 h, resulting in a color change from intense orange to bright yellow. The solution was then filtered and freeze-dried to afford **5-H** as a bright yellow powder in 90% yield (38.0 mg, 71.7 μmol). Suitable crystals for X-ray diffraction analysis were obtained by slow evaporation of a saturated pentane solution at −30 °C. ^{1}H NMR (500.1 MHz, C_6D_6):

δ = 7.36 (s, 2H, *m*-C*H*^{Tip}), 7.29 (dd, ^3J = 8.6 Hz, ^3J = 6.3 Hz, 1H, *p*-C*H*^{Dip}), 7.24 (dd, ^3J = 7.6 Hz, ^4J = 1.7 Hz, 2H, *m*-C*H*^{Dip}), 5.84 (s, 1H, O*H*), 5.56 (s, 1H, N*H*), 3.93 (sept, ^3J = 6.9 Hz, 2H, C*H*$(CH_3)_2^{Dip}$), 3.66 (sept, ^3J = 6.8 Hz, 2H, C*H*$(CH_3)_2^{Tip}$), 2.99 (sept, ^3J = 6.9 Hz, 1H, C*H*$(CH_3)_2^{Tip}$), 1.51 (d, ^3J = 6.8 Hz, 12H, $CH(CH_3)_2^{Tip}$), 1.38 (d, ^3J = 6.9 Hz, 6H, $CH(CH_3)_2^{Tip}$), 1.31 (d, ^3J = 6.8 Hz, 6H, $CH(CH_3)_2^{Dip}$), 1.28 (d, ^3J = 6.9 Hz, 6H, $CH(CH_3)_2^{Dip}$), 1.14 (s, 2H, CH_2^{cAAC}), 0.99 (s, 6H, CH_3^{cAAC}), 0.65 (s, 6H, CH_3^{cAAC}) ppm. $^{13}C\{^1H\}$ NMR (125.8 MHz, C_6D_6): δ = 199.3 ($C_{carbene}$, identified by HMBC), 154.5 (*o*-C_q^{Tip}), 148.4 (*o*-C_q^{Dip}), 148.2 (*p*-C_q^{Tip}), 142.4 (*i*-C_q^{Dip}), 128.6 (*p*-CH^{Dip}), 124.2 (*m*-CH^{Dip}), 121.0 (*m*-CH^{Tip}), 62.2 (C_q^{cAAC}), 51.9 (CH_2^{cAAC}), 51.5 (C_q^{cAAC}), 35.1 ($CH(CH_3)_2^{Tip}$), 34.9 ($CH(CH_3)_2^{Tip}$), 30.4 ($CH(CH_3)_2^{Dip}$), 30.1 (CH_3^{cAAC}), 29.4 (CH_3^{cAAC}), 28.3 ($CH(CH_3)_2^{Tip}$), 25.3 ($CH(CH_3)_2^{Dip}$), 24.6 ($CH(CH_3)_2^{Tip}$), 24.4 ($CH(CH_3)_2^{Dip}$), 23.5 ($CH(CH_3)_2^{Tip}$) ppm. *Note: The resonances of the alkylideneborane and i-C^{Tip} carbon nuclei were not detected due to quadrupolar line broadening.* ^{11}B NMR (160.5 MHz): δ = 15.3 (s) ppm. UV-vis (THF): λ_{max} = 430 nm. IR (solid state): ṽ(OH) = 3477 cm^{-1}, ṽ(NH) = 3329 cm^{-1}. HRMS (LIFDI) calculated for [$C_{36}H_{56}BNO$] = [M]: 529.4449; found: 529.4445.

Synthesis of compound 5-Me

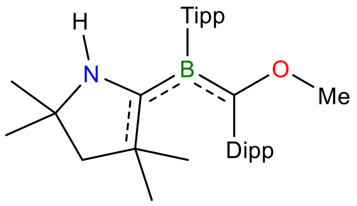

To a solution of **4-H** (28.0 mg, 26.4 μmol) in benzene (1 mL), methyltriflate (15.6 mg, 105 μmol) was slowly added, resulting in an immediate color change from orange to bright yellow concomitant with precipitation of a solid. After stirring for one hour, the reaction mixture was filtered, the solid residue extracted with benzene (0.5 mL) and the filtrate dried *in vacuo*. The resulting yellow powder was recrystallized from pentane (1 mL) at −30 °C to afford **5-Me** as yellow crystals in 52% yield (15.0 mg, 27.6 μmol). Suitable crystals for X-ray diffraction analysis were obtained by slow evaporation of a saturated pentane solution at −30 °C. ^1H NMR (500.1 MHz, C_6D_6): δ = 7.33 (s, 2H, *m*-C*H*^{Tip}), 7.23 (dd, ^3J = 8.6 Hz, ^3J = 6.7 Hz, 1H, *p*-C*H*^{Dip}), 7.18 (dd, 2H, *m*-C*H*^{Dip}), 5.35 (s, 1H, N*H*^{cAAC}), 3.77 (sept, ^3J = 6.9 Hz, 2H, C*H*$(CH_3)_2^{Dip}$), 3.64 (sept, ^3J = 6.8 Hz, 2H, C*H*$(CH_3)_2^{Tip}$), 3.09 (s, 3H, OCH_3), 2.99 (sept, ^3J = 6.9 Hz, 1H, C*H*$(CH_3)_2^{Tip}$), 1.61 (d, ^3J = 6.7 Hz, 6H, $CH(CH_3)_2^{Tip}$), 1.59 (d, ^3J = 6.9 Hz,

6H, $CH(CH_3)_2^{Tip}$), 1.36 (d, ^3J = 6.9 Hz, 6H, $CH(CH_3)_2^{Tip}$), 1.24 (d, ^3J = 6.6 Hz, 6H, $CH(CH_3)_2^{Dip}$), 1.23 (d, ^3J = 6.7 Hz, 6H, $CH(CH_3)_2^{Dip}$), 1.12 (s, 2H, CH_2^{cAAC}), 1.00 (s, 6H, CH_3^{cAAC}), 0.64 (s, 6H, CH_3^{cAAC}) ppm. *Note: the resonance of the m-CH^{Dip} protons partially overlaps with the deuterated solvent resonance at 7.16 ppm, thus the coupling constant cannot be determined.* $^{13}C\{^1H\}$ NMR (125.8 MHz, C_6D_6): δ = 200.8 ($C_{carbene}$, identified by HMBC), 175.5 (BC_qO, identified by HMBC), 152.3 (o-C_q^{Tip}), 148.4 (o-C_q^{Dip}), 146.3 (p-C_q^{Tip}), 143.0 (i-C_q^{Tip}, identified by HMBC), 140.8 (i-C_q^{Dip}), 128.8 (p-CH^{Dip}), 124.5 (m-CH^{Dip}), 119.6 (m-CH^{Tip}), 61.8 (C_q^{cAAC}), , 54.6 (OCH_3), 52.3 (CH_2^{cAAC}), 52.1 (C_q^{cAAC}), 35.3 ($CH(CH_3)_2^{Tip}$), 34.9 ($CH(CH_3)_2^{Tip}$), 30.6 ($CH(CH_3)_2^{Dip}$), 30.2 (CH_3^{cAAC}), 29.3 (CH_3^{cAAC}), 27.8 ($CH(CH_3)_2^{Tip}$), 25.0 ($CH(CH_3)_2^{Dip}$), 24.7 ($CH(CH_3)_2^{Tip}$), 23.8 ($CH(CH_3)_2^{Dip}$), 23.0 ($CH(CH_3)_2^{Tip}$) ppm. ^{11}B NMR (160.5 MHz): δ = 16.7 (s) ppm. UV-vis (THF): λ_{max} = 444 nm. IR (solid state): ṽ(NH) = 3354 cm^{-1}. HRMS (LIFDI) calculated for [$C_{37}H_{58}BNO$] = [M]: 543.4606; found: 543.4601.

Synthesis of compound 5-TMS

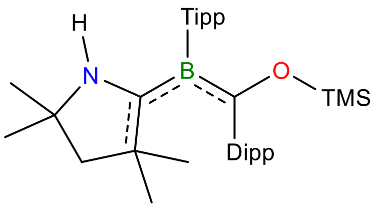

To a solution of **4-H** (10.0 mg, 8.81 μmol) in benzene (1 mL), trimethylchlorosilane (3.83 mg, 35.2 μmol) was slowly added, resulting in an immediate color change from orange to bright yellow concomitant with precipitation of a solid. After stirring for one hour all volatiles were removed *in vacuo*, the solid extracted with benzene and subsequently freeze dried to afford **5-TMS** as a yellow powder. The solid was then dissolved and recrystallized from pentane (0.5 mL) at −30 °C to afford **5-TMS** as yellow crystals in 85% yield (9.00 mg, 15.0 μmol). Suitable crystals for X-ray diffraction analysis were obtained by slow evaporation of a saturated pentane solution at −30 °C. ^1H NMR (500.1 MHz, C_6D_6): δ = 7.30 (s, 2H, m-CH^{Tip}), 7.22 (dd, ^3J = 8.8 Hz, ^3J = 6.2 Hz, 1H, p-CH^{Dip}), 7.17 (dd, ^4J = 0.6 Hz, 2H, m-CH^{Dip}), 5.41 (s, 1H, NH^{cAAC}), 3.77 (sept, ^3J = 6.9 Hz, 2H, $CH(CH_3)_2^{Dip}$), 3.57 (sept, ^3J = 6.8 Hz, 2H, $CH(CH_3)_2^{Tip}$), 3.00 (sept, ^3J = 6.9 Hz, 1H, $CH(CH_3)_2^{Tip}$), 1.61 (d, ^3J = 6.8 Hz, 6H, $CH(CH_3)_2^{Tip}$), 1.56 (d, ^3J = 6.8 Hz, 6H, $CH(CH_3)_2^{Tip}$), 1.38 (d, ^3J = 6.9 Hz, 6H, $CH(CH_3)_2^{Tip}$), 1.36 (d, ^3J = 6.9 Hz, 6H, $CH(CH_3)_2^{Dip}$), 1.25 (d, ^3J = 6.8 Hz, 6H, $CH(CH_3)_2^{Dip}$), 1.10 (s, 2H, CH_2^{cAAC}), 0.98 (s, 6H,

CH_3^{cAAC}), 0.64 (s, 6H, CH_3^{cAAC}), −0.10 (s, 9H, CH_3^{TMS}) ppm. *Note: the resonance of the m-CH^{Dip} protons partially overlaps with the deuterated solvent resonance at 7.16 ppm*. $^{13}C\{^1H\}$ NMR (125.8 MHz, C_6D_6): δ = 203.0 ($C_{carbene}$, identified by HMBC) 152.4 (*o*-C_q^{Tip}), 147.6 (*o*-C_q^{Dip}), 146.4 (*i*-C_q^{Dip}), 145.2 (*p*-C_q^{Tip}), 143.0 (*i*-C_q^{Tip}, identified by HMBC), 128.4 (*p*-CH^{Dip}), 124.9 (*m*-CH^{Dip}), 119.5 (*m*-CH^{Tip}), 62.2 (C_q^{cAAC}), 52.5 (C_q^{cAAC}), 52.2 (CH_2^{cAAC}), 35.2 ($CH(CH_3)_2^{Tip}$), 34.9 ($CH(CH_3)_2^{Tip}$), 30.2 (CH_3^{cAAC}), 30.1 ($CH(CH_3)_2^{Dip}$), 29.3 (CH_3^{cAAC}), 28.2 ($CH(CH_3)_2^{Tip}$), 26.1 ($CH(CH_3)_2^{Dip}$), 24.7 ($CH(CH_3)_2^{Tip}$), 23.9 ($CH(CH_3)_2^{Dip}$), 23.3 ($CH(CH_3)_2^{Tip}$), 2.6 (CH_3^{TMS}) ppm. *Note: The resonance of the alkylideneborane carbon nucleus was not detected due to quadrupolar line broadening; the ^3J coupling constant of the doublet at 7.17 ppm cannot be determined due to overlapping with the resonance of the deuterated solvent.* ^{11}B NMR (160.5 MHz): δ = 18.1 (s) ppm. ^{29}Si NMR (99.4 MHz): δ = 8.7 (s) ppm. UV-vis (THF): λ_{max} = 440 nm. IR (solid state): ṽ(NH) = 3336 cm^{-1}. HRMS (LIFDI) calculated for [$C_{39}H_{64}BNOSi$] = [M]: 601.4845; found: 601.4837.

NMR spectra of isolated compounds

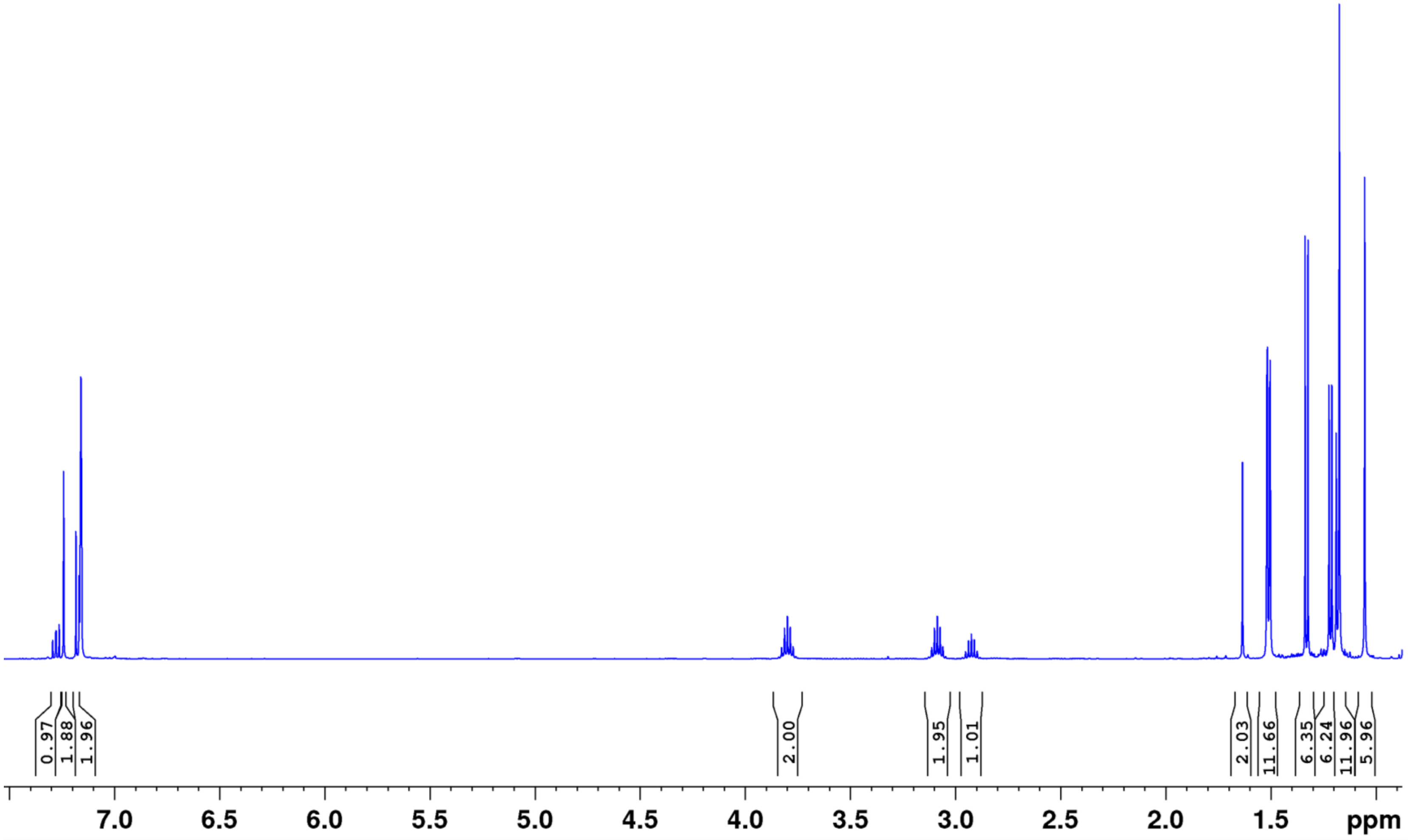

Figure S1. ^{1}H NMR spectrum of **2** in C_6D_6.

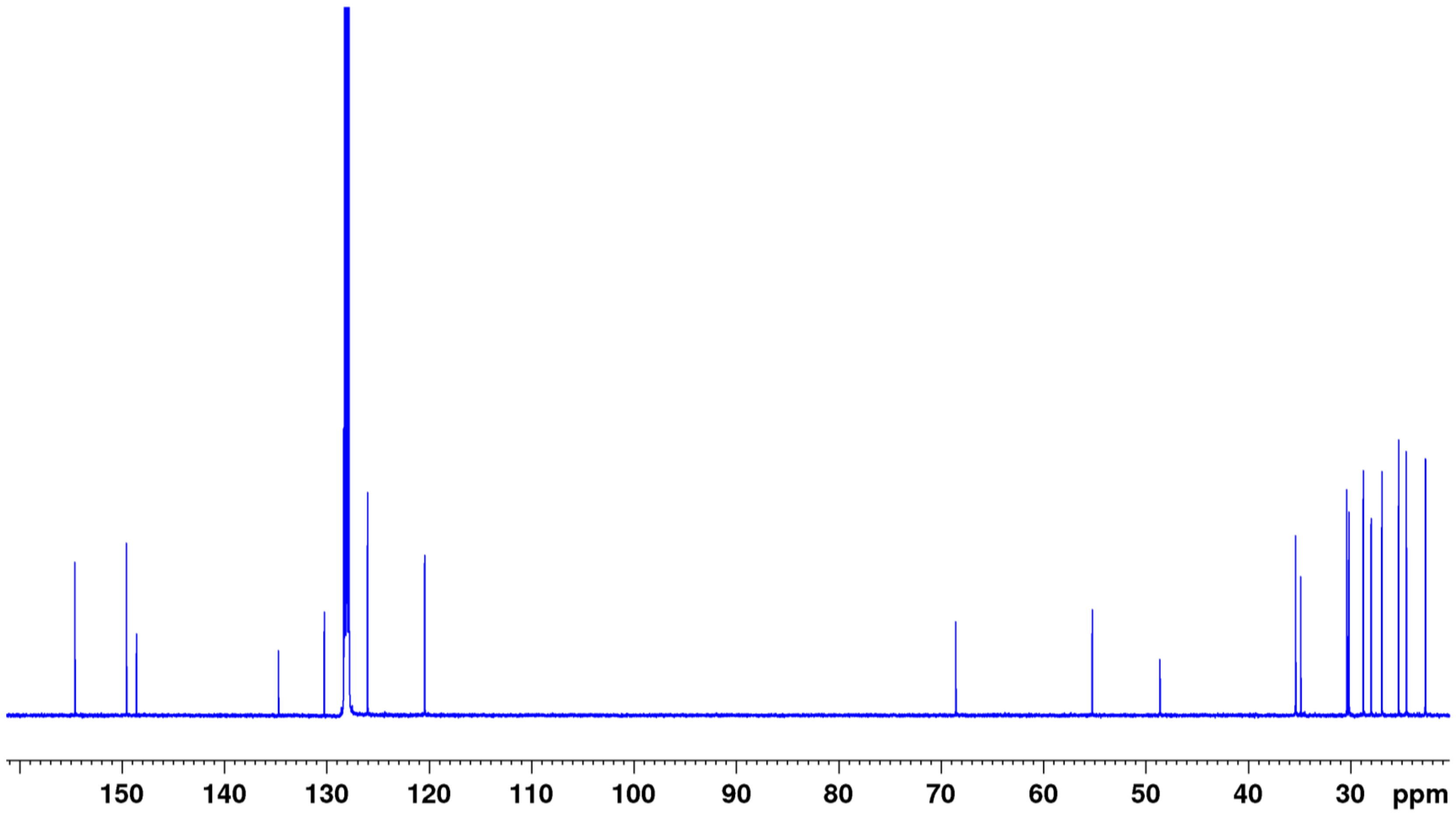

Figure S2. $^{13}C\{^{1}H\}$ NMR spectrum of **2** in C_6D_6.

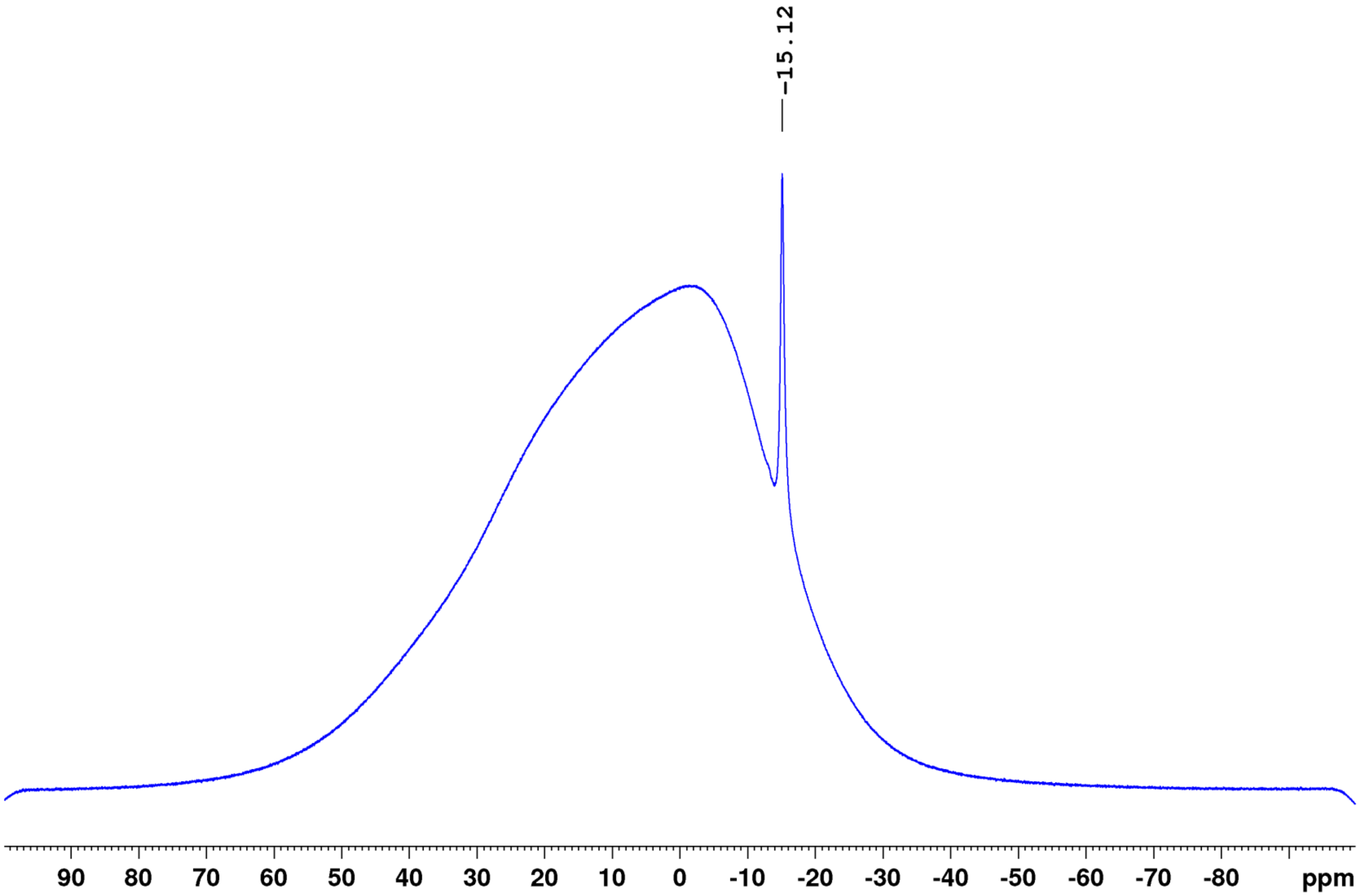

Figure S3. ^{11}B NMR spectrum of **2** in C_6D_6.

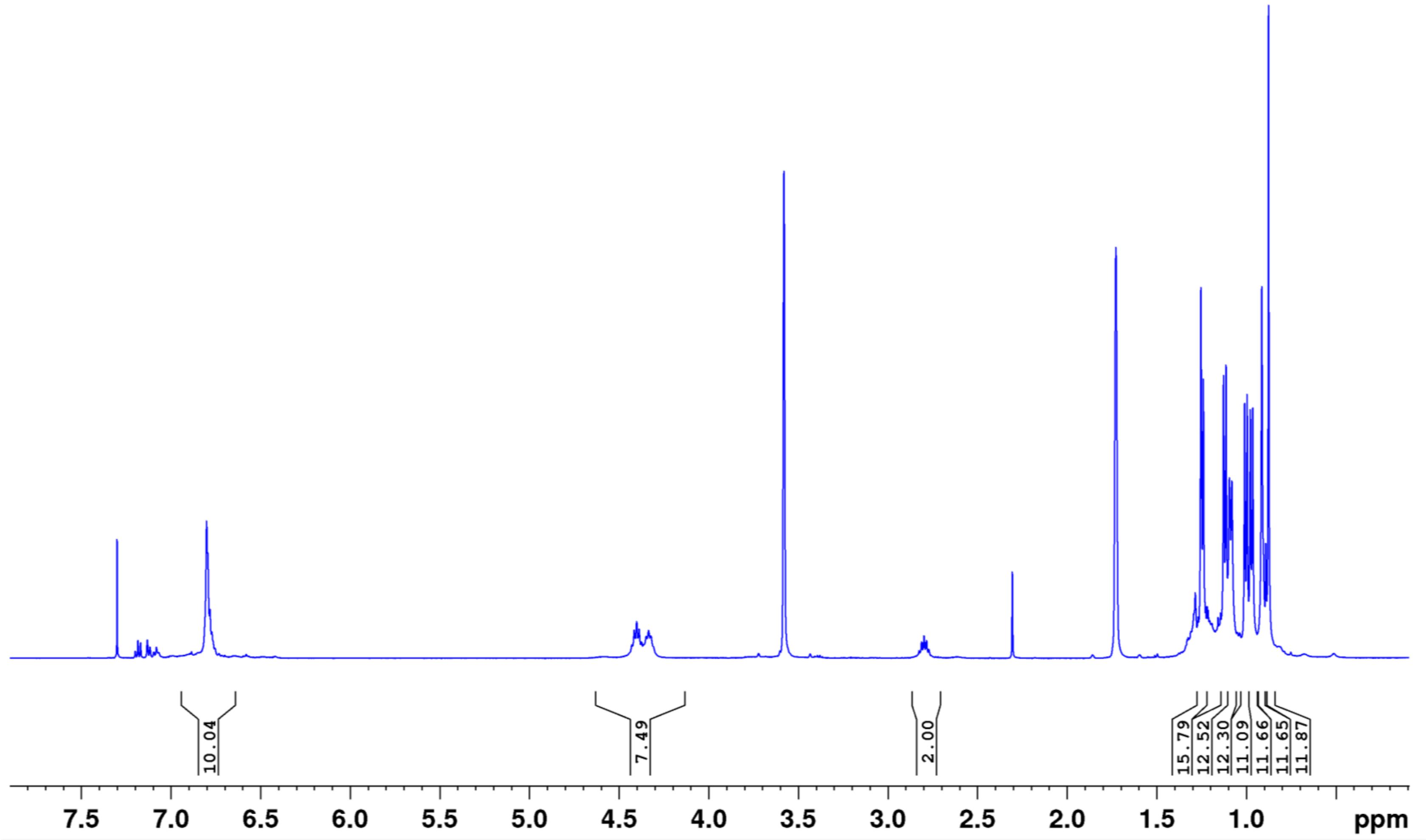

Figure S4. ^{1}H NMR spectrum of **4-K** in d_8-thf.

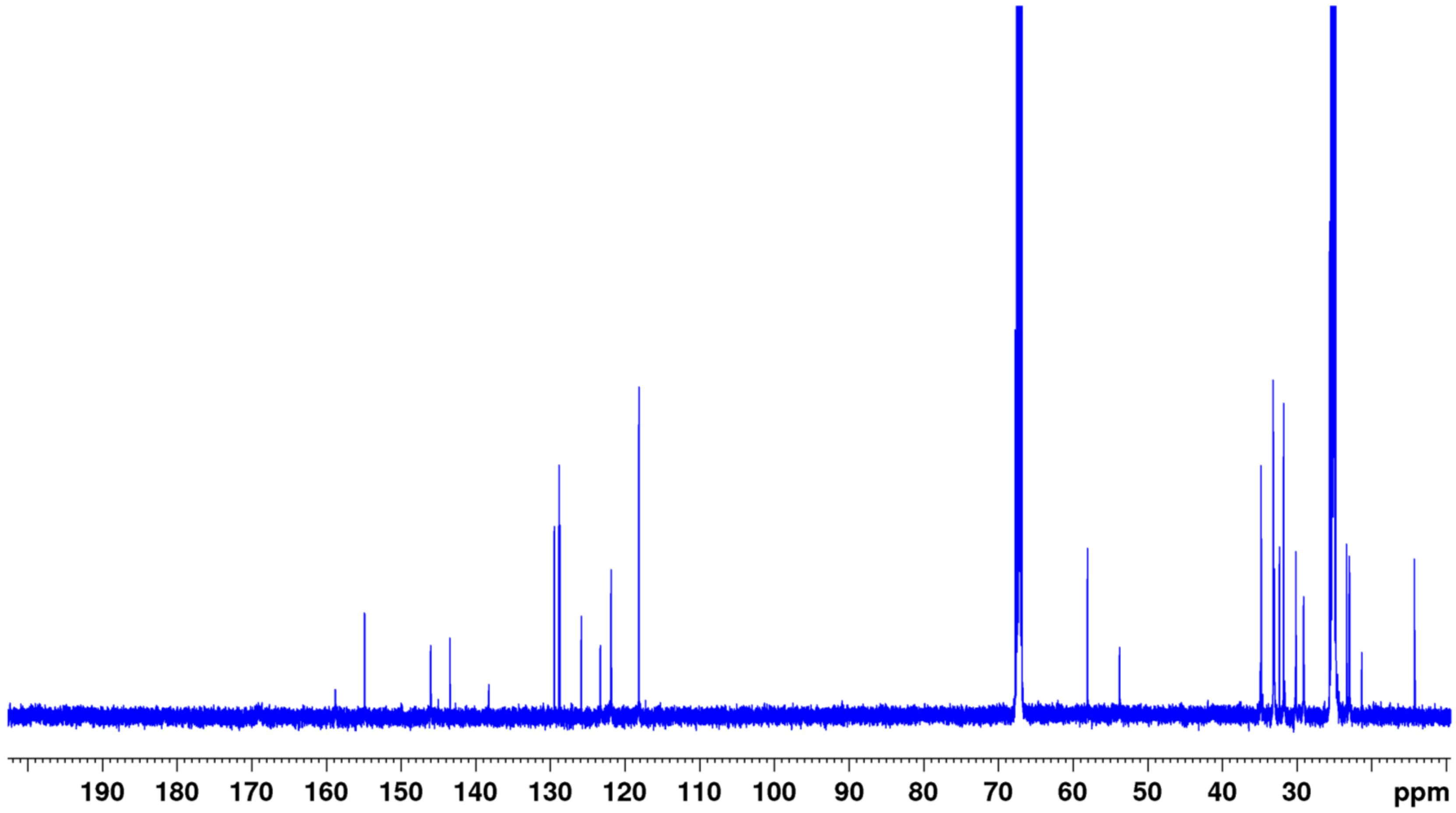

Figure S5. $^{13}C\{^1H\}$ NMR spectrum of **4-K** in d_8-thf.

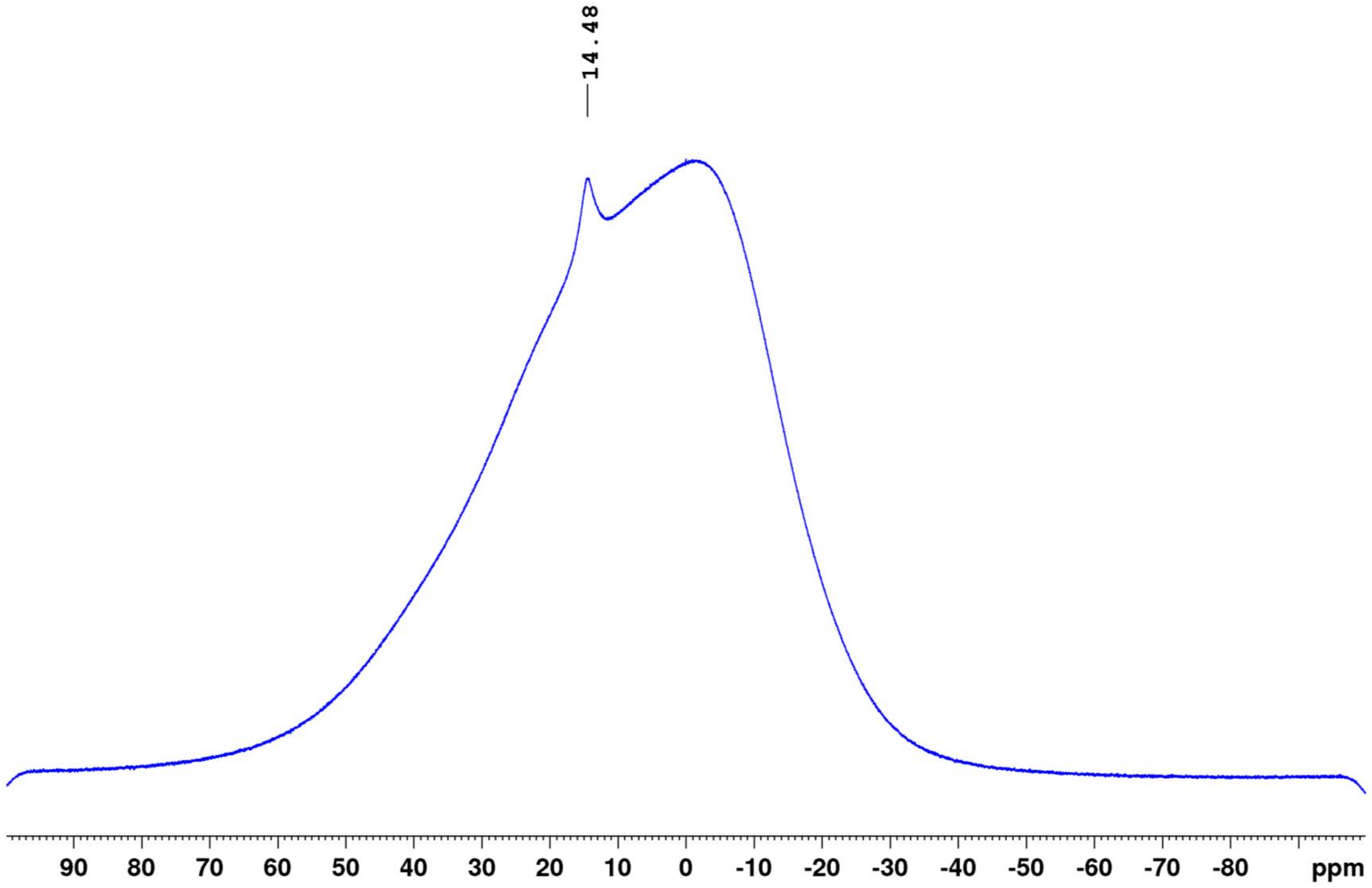

Figure S6. ^{11}B NMR spectrum of **4-K** in d_8-thf.

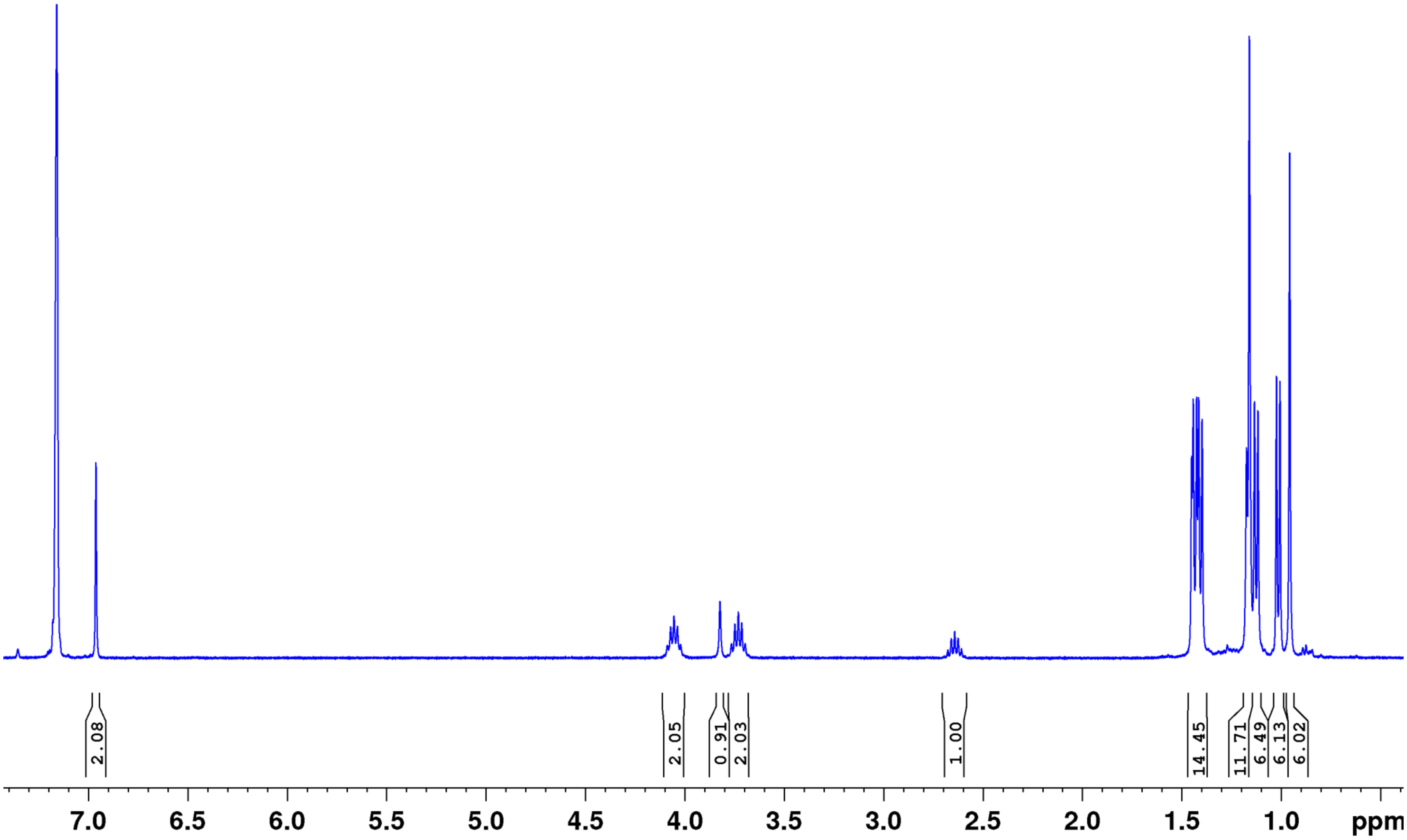

Figure S7. ^{1}H NMR spectrum of **4-H** in C_6D_6.

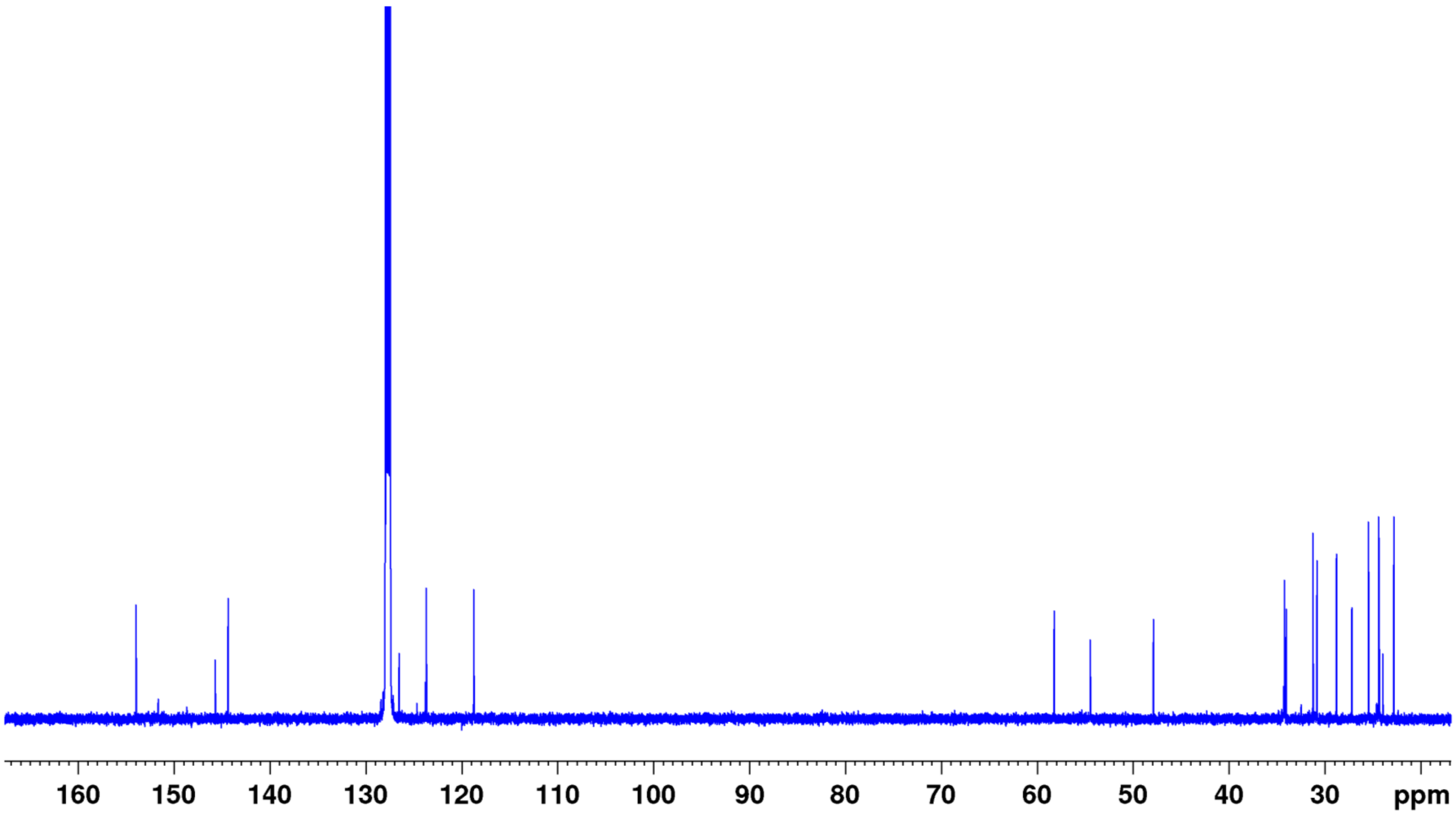

Figure S8. $^{13}C\{^1H\}$ NMR spectrum of **4-H** in C_6D_6.

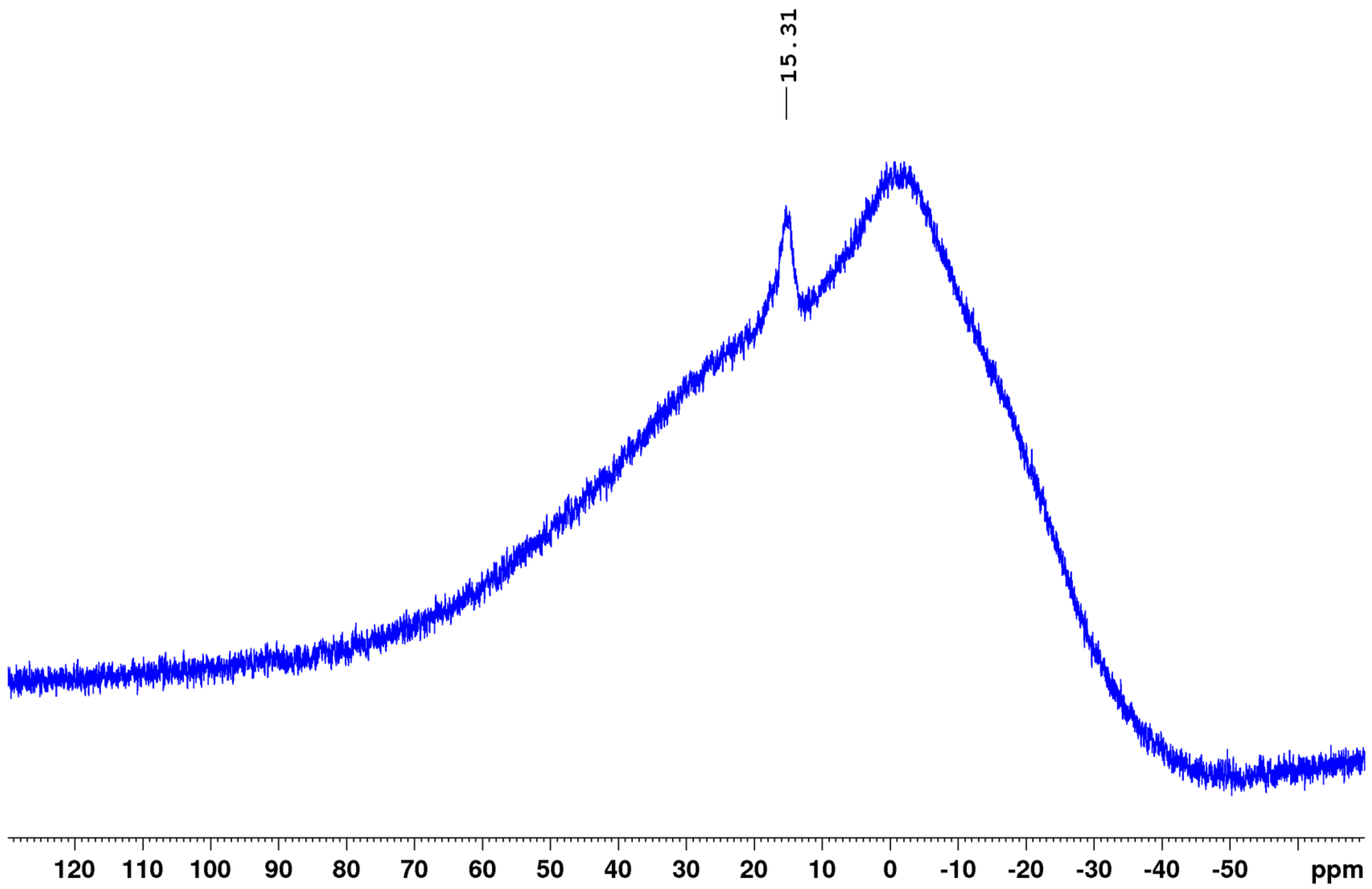

Figure S9. ^{11}B NMR spectrum of **4-H** in C_6D_6.

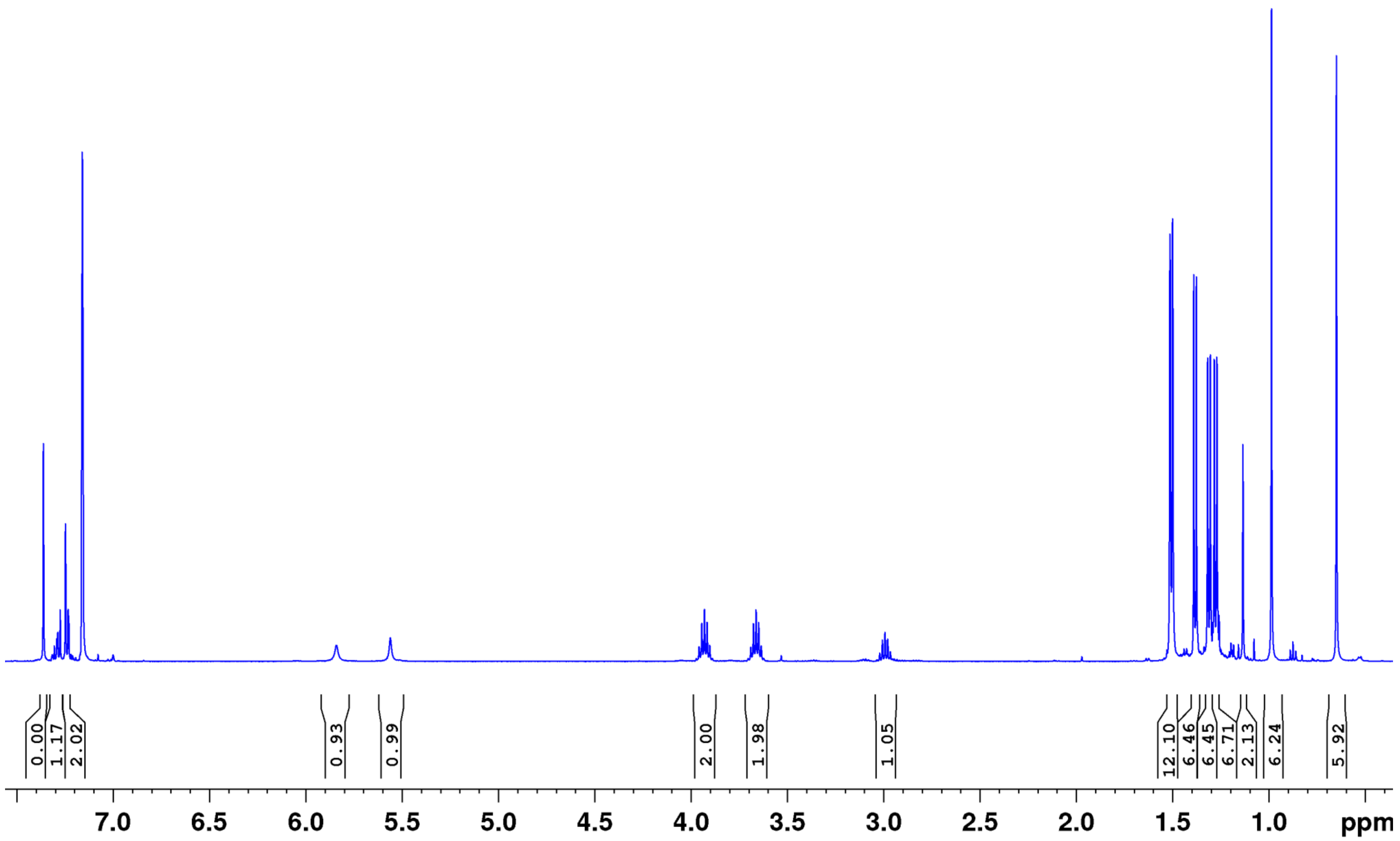

Figure S10. ^{1}H NMR spectrum of **5-H** in C_6D_6.

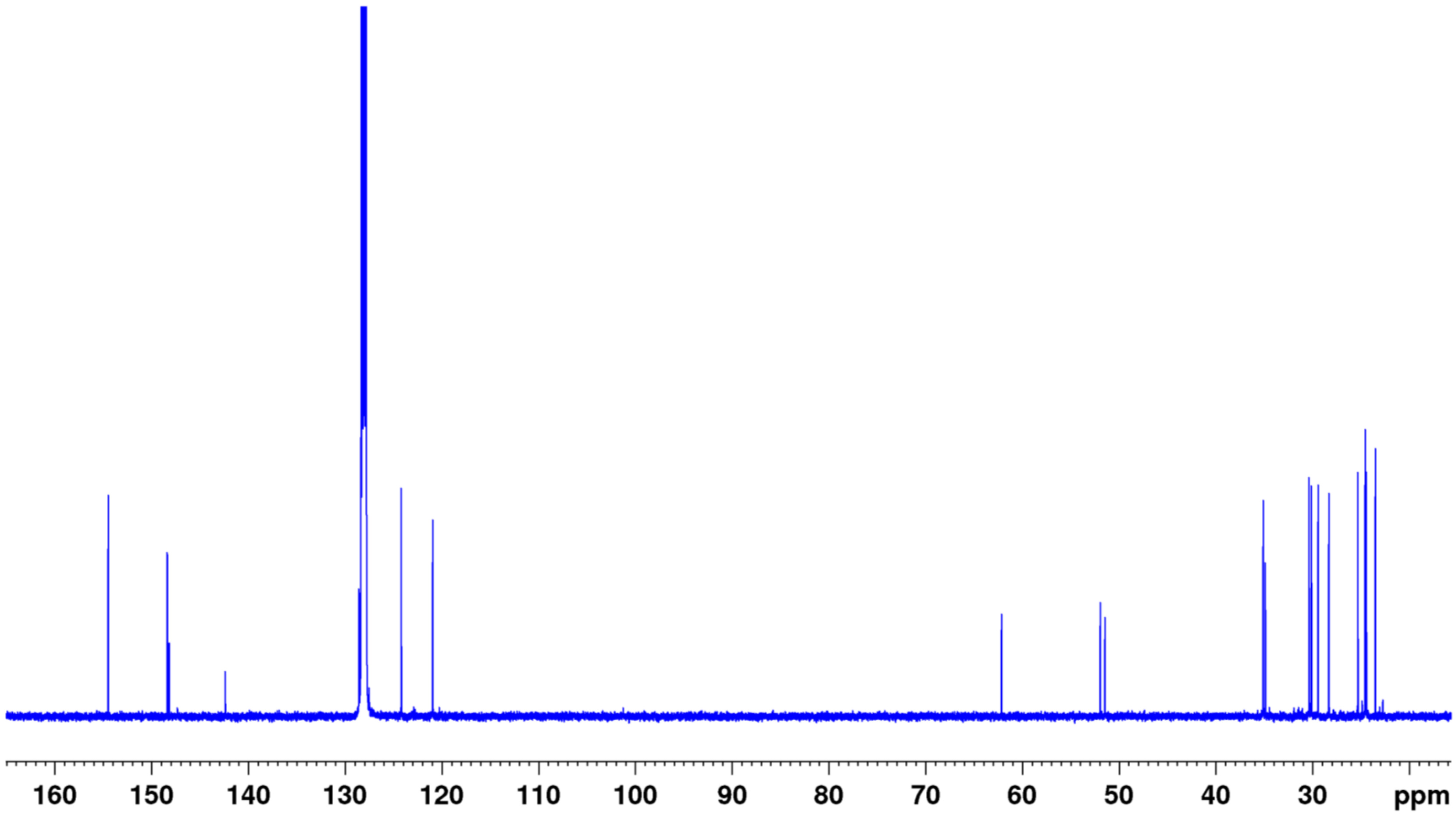

Figure S11. $^{13}C\{^1H\}$ NMR spectrum of **5-H** in C_6D_6.

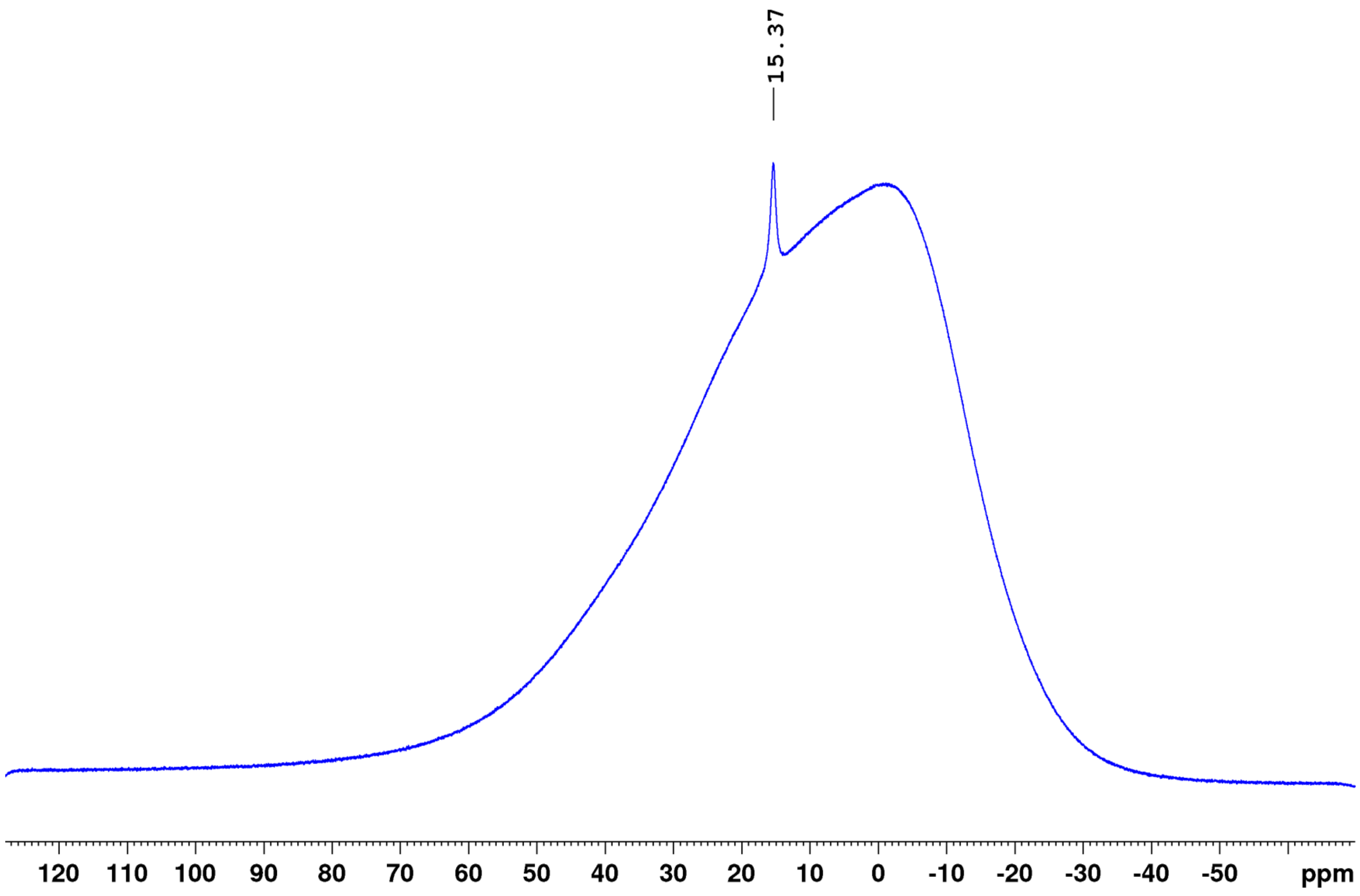

Figure S12. ^{11}B NMR spectrum of **5-H** in C_6D_6.

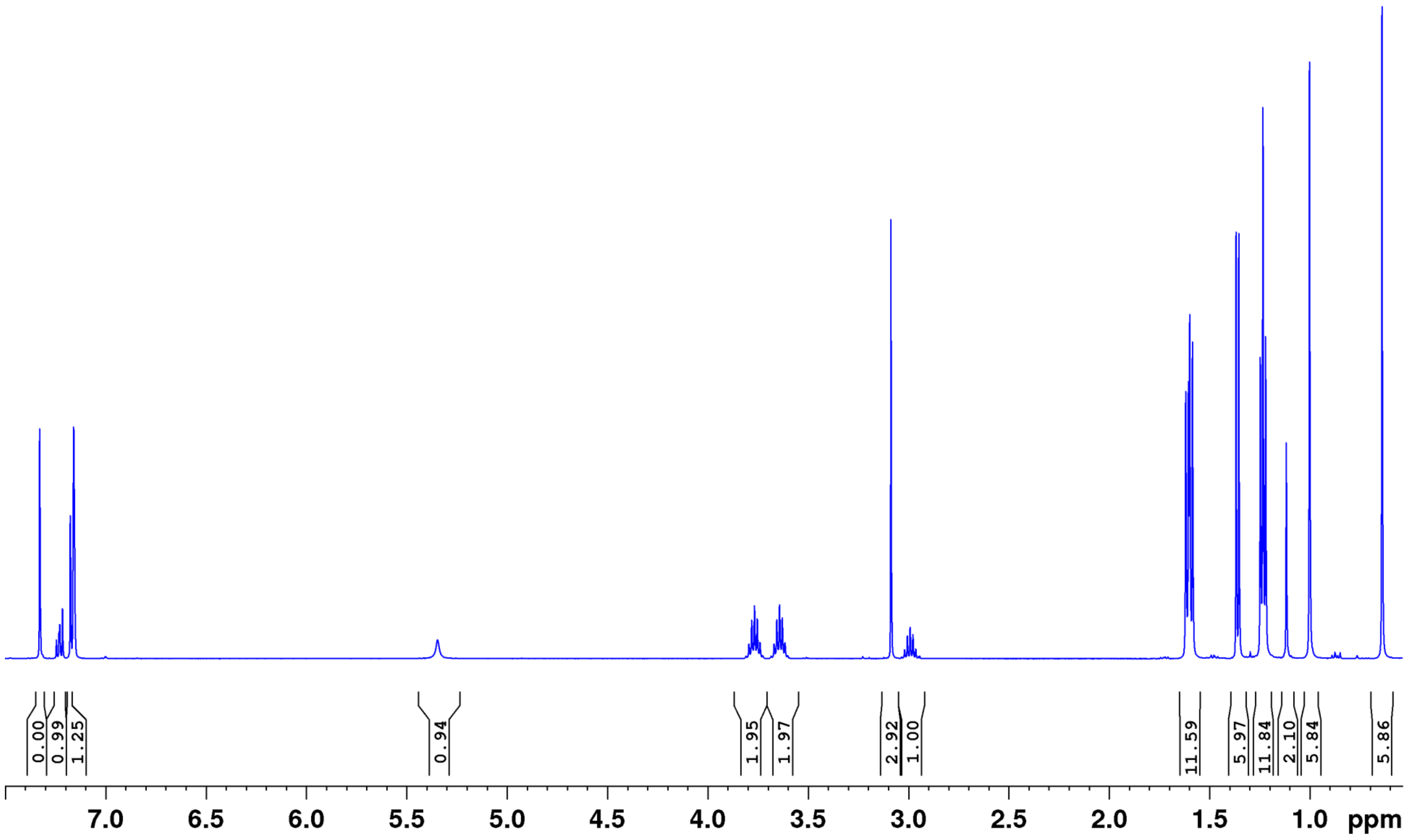

Figure S13. ^{1}H NMR spectrum of **5-Me** in C_6D_6.

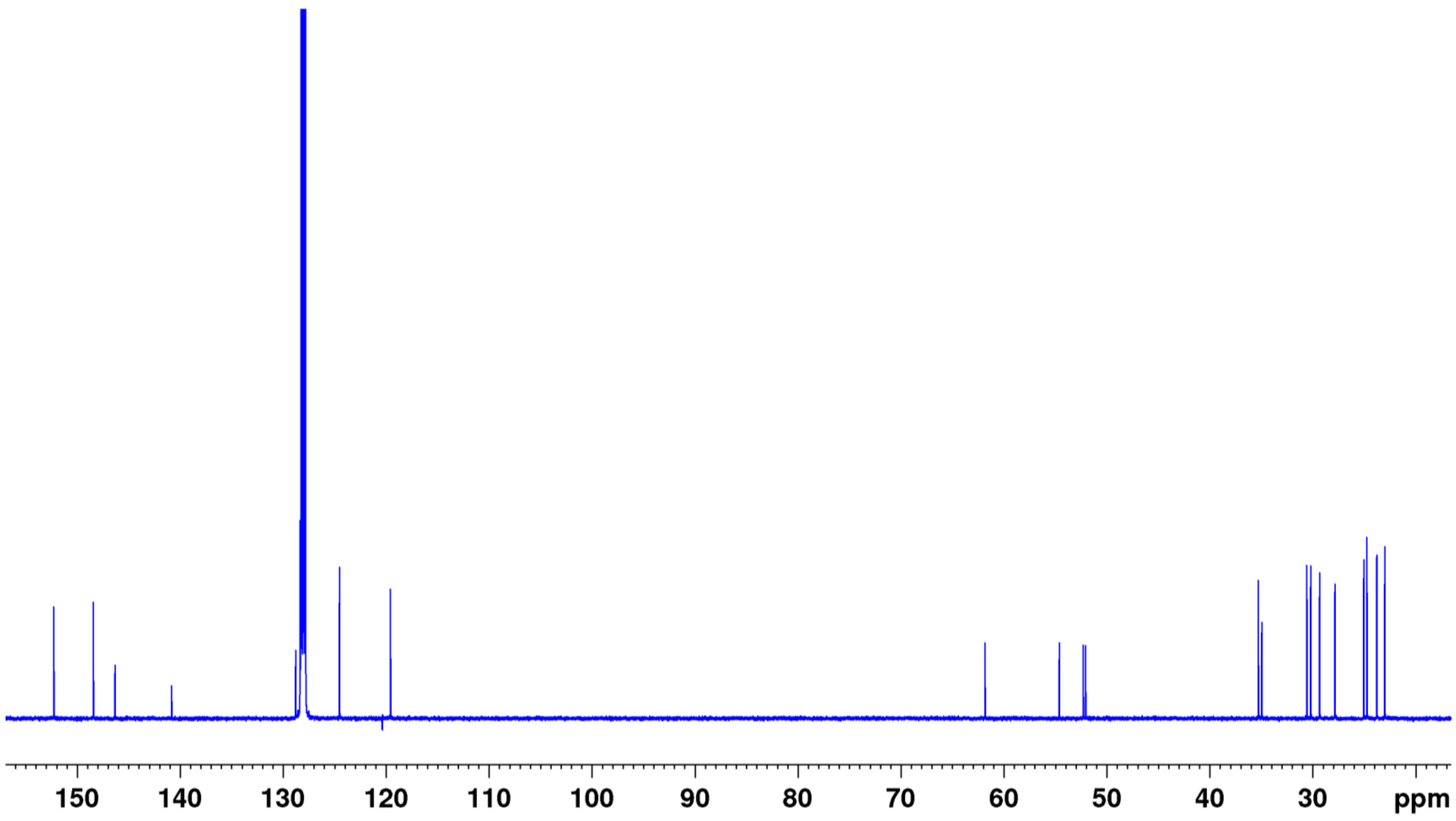

Figure S14. $^{13}C\{^1H\}$ NMR spectrum of **5-Me** in C_6D_6.

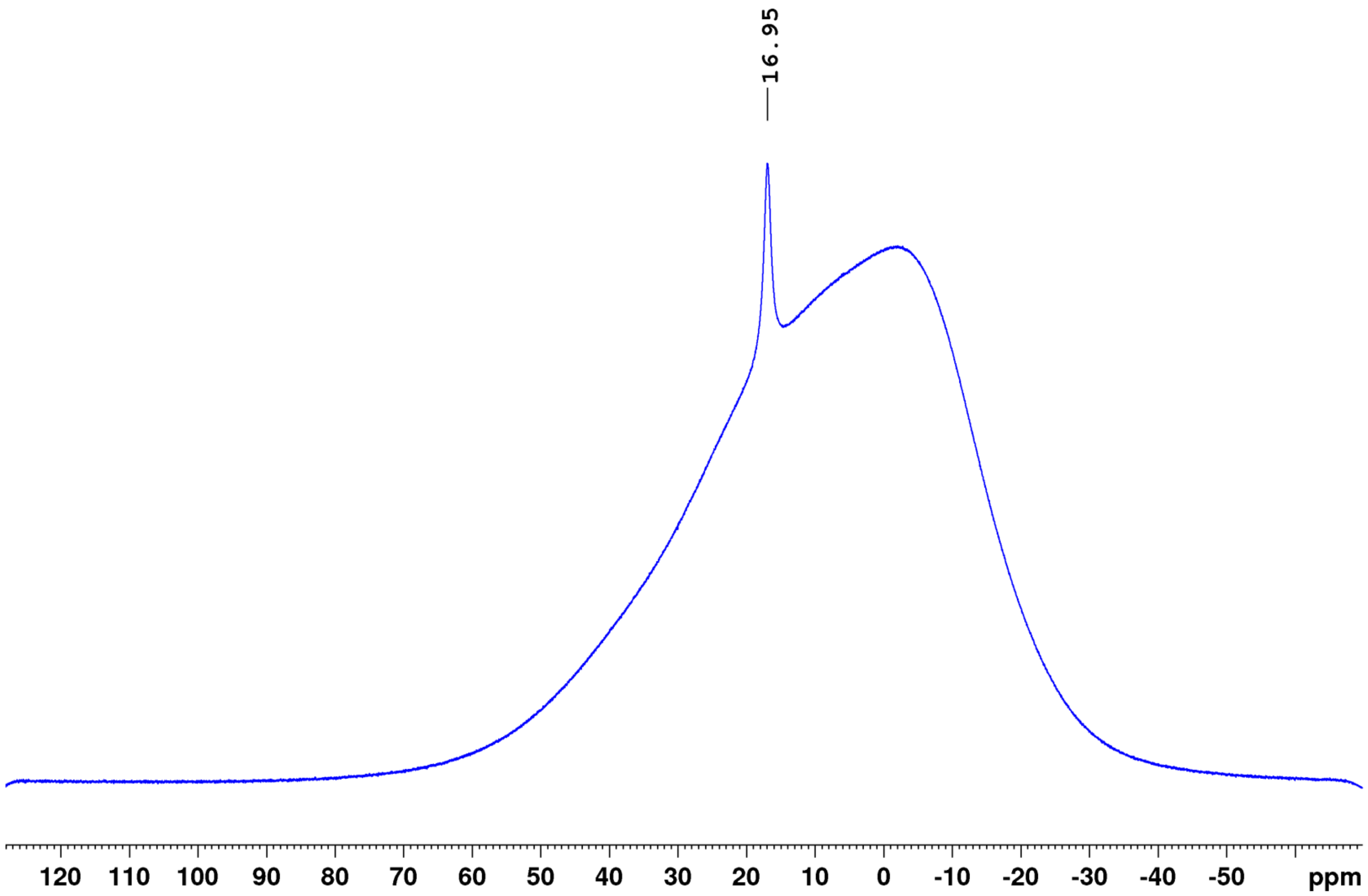

Figure S15. ^{11}B NMR spectrum of **5-Me** in C_6D_6.

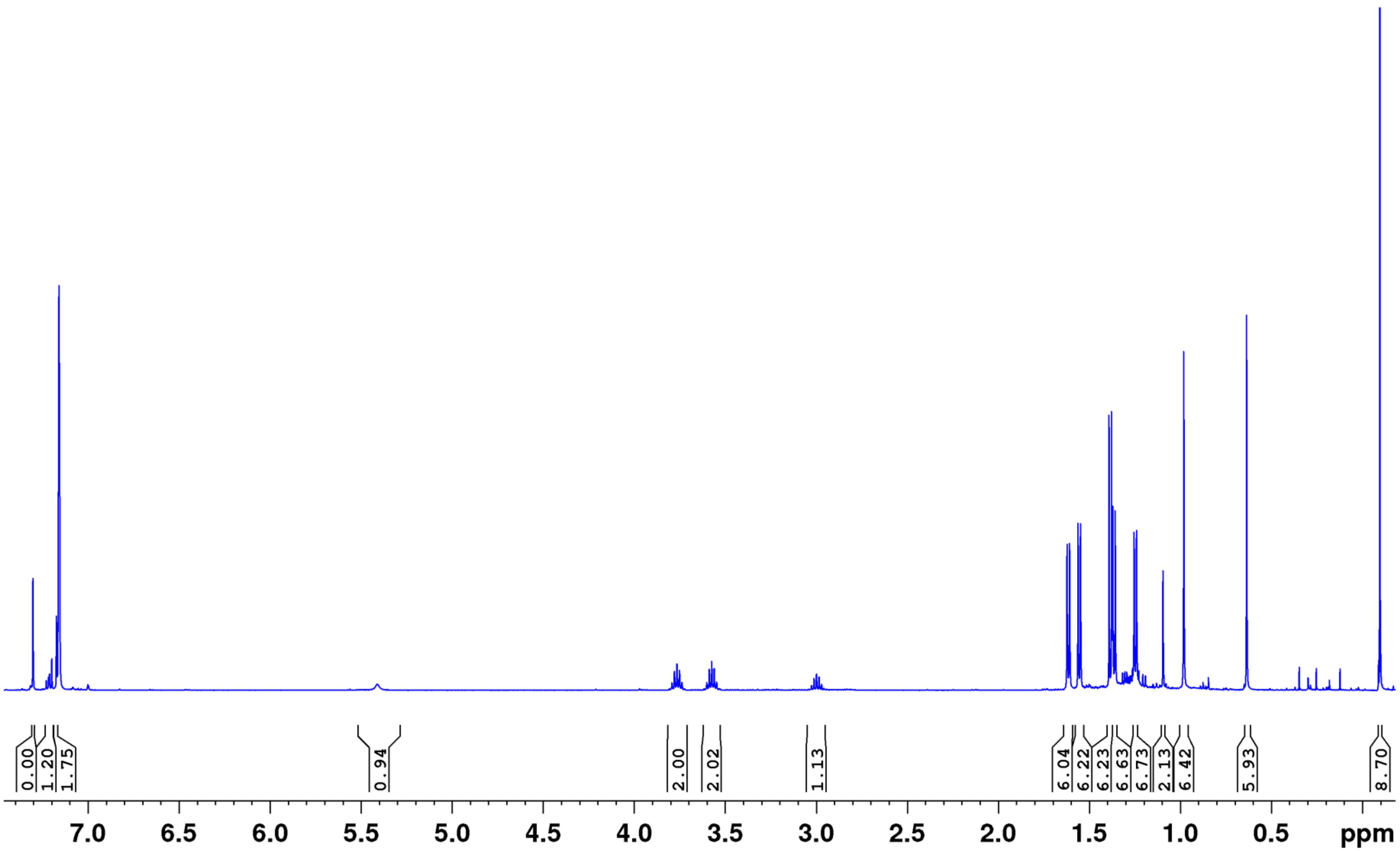

Figure S16. ^{1}H NMR spectrum of **5-TMS** in C_6D_6.

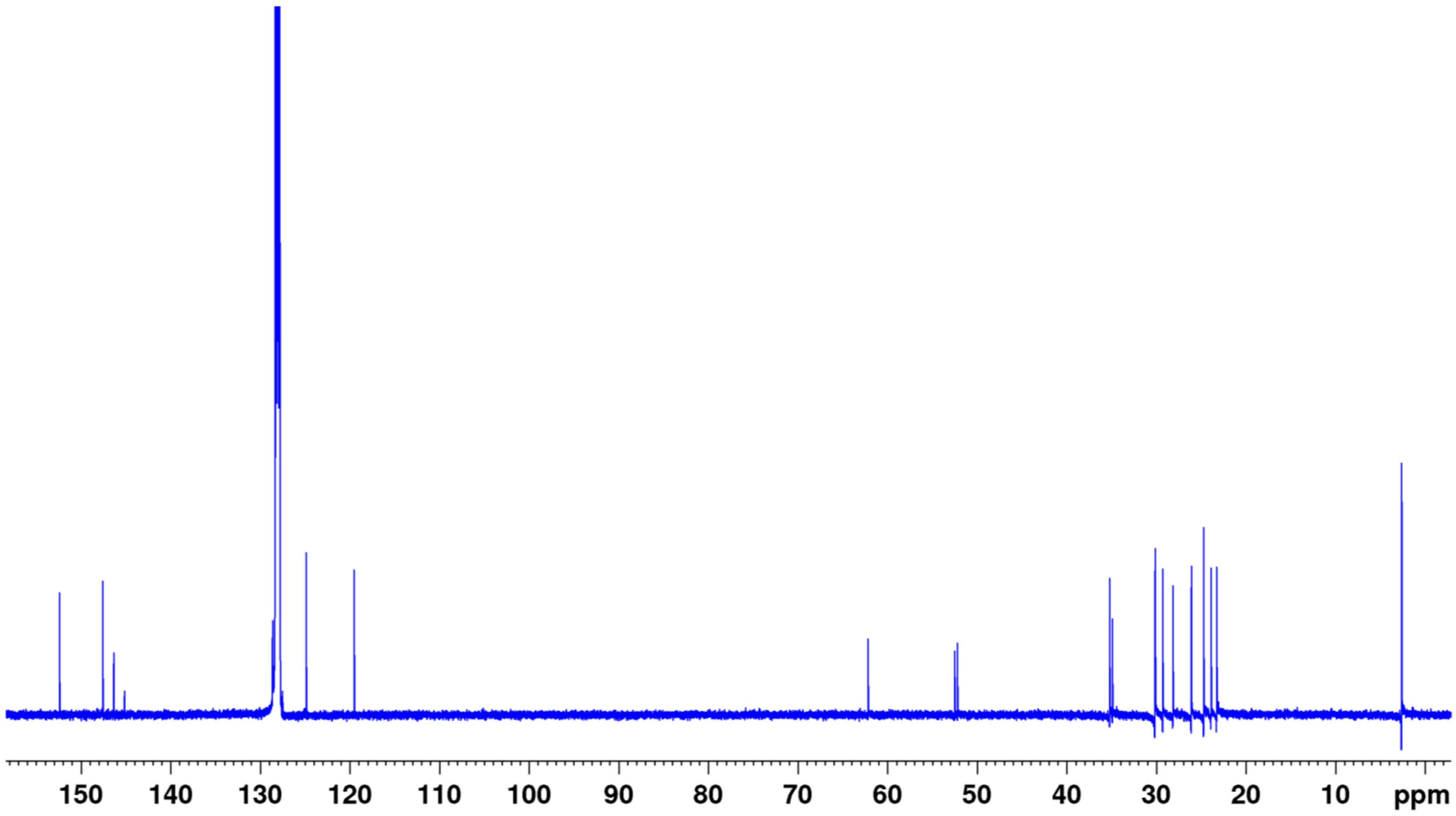

Figure S17. $^{13}C\{^1H\}$ NMR spectrum of **5-TMS** in C_6D_6.

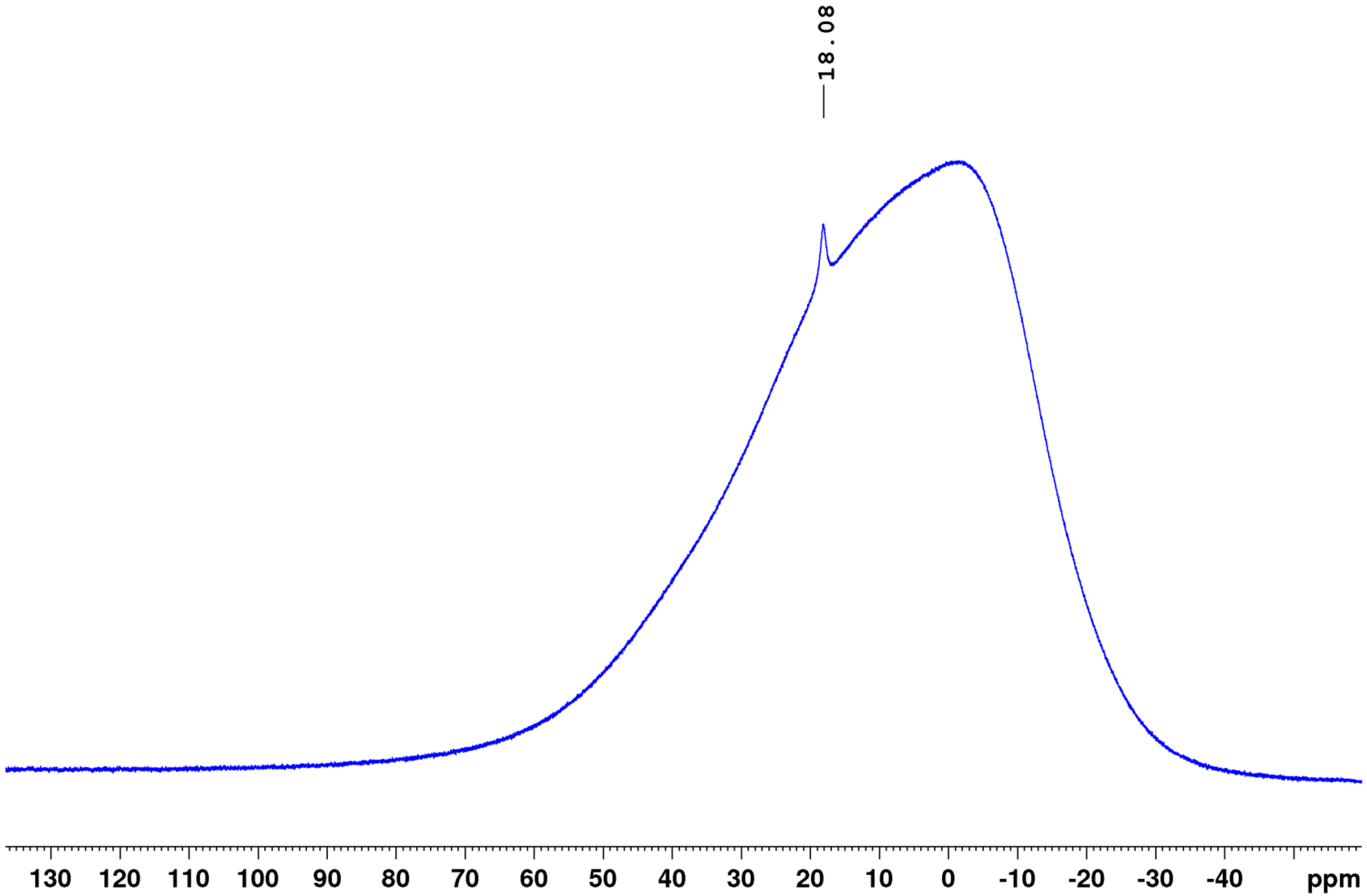

Figure S18. ^{11}B NMR spectrum of **5-TMS** in C_6D_6.

EPR-spectroscopic study of 3

EPR measurements at X-band (9.38 GHz) were carried out using a Bruker ELEXSYS E580 CW EPR spectrometer equipped with an Oxford Instruments helium cryostat (ESR900) and a MercuryiTC temperature controller. The spectral analysis was performed using MATLAB 8.6.0.267246 (R2015b) and the Optimization toolbox. Spectral simulations were performed using MATLAB 8.6 (R2015b) and the EasySpin 5.2.25 toolbox.[2]

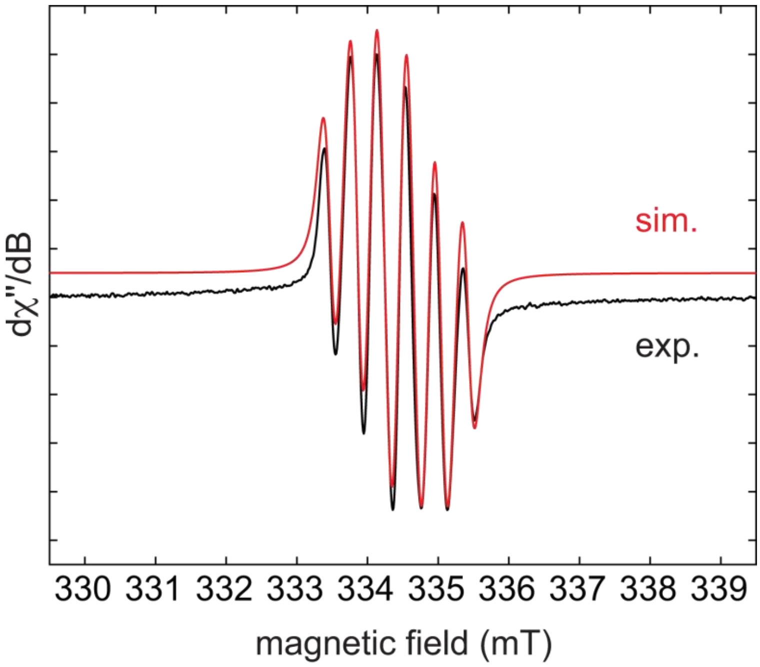

Figure S19. Experimental (black) and simulated (red) continuous-wave X-band EPR spectra of **3** in a toluene/thf mixture at 270 K. Simulation parameters: g_{iso} = 2.0040, $a(^{11}B)$ = 11.7 MHz (4.2 G) and $a(^{14}N)$ = 9.8 MHz (3.5 G).

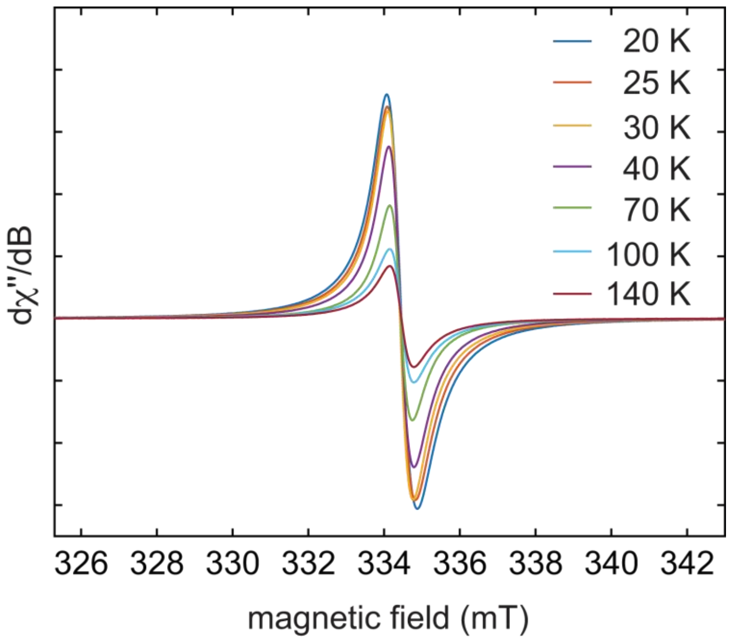

Figure S20. Variable temperature X-band EPR spectra of solid **3** diluted in KBr between 20 and 140 K. The EPR spectra recorded for neat powders are essentially identical.

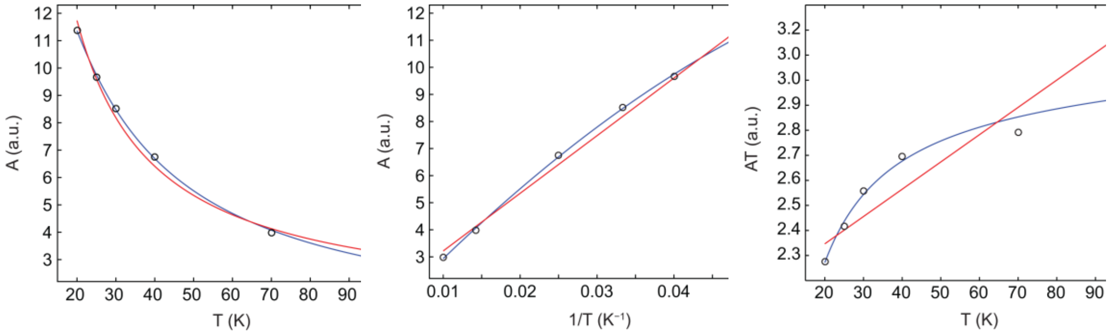

Figure S21. Three different representations of the temperature dependence of the double integral EPR intensity (*A*) of **3**. Circles (∘) represent the experimental results and the red line corresponds to the fit with the Bleaney-Bowers equation and the red line represents Curie behavior.

UV-vis spectra

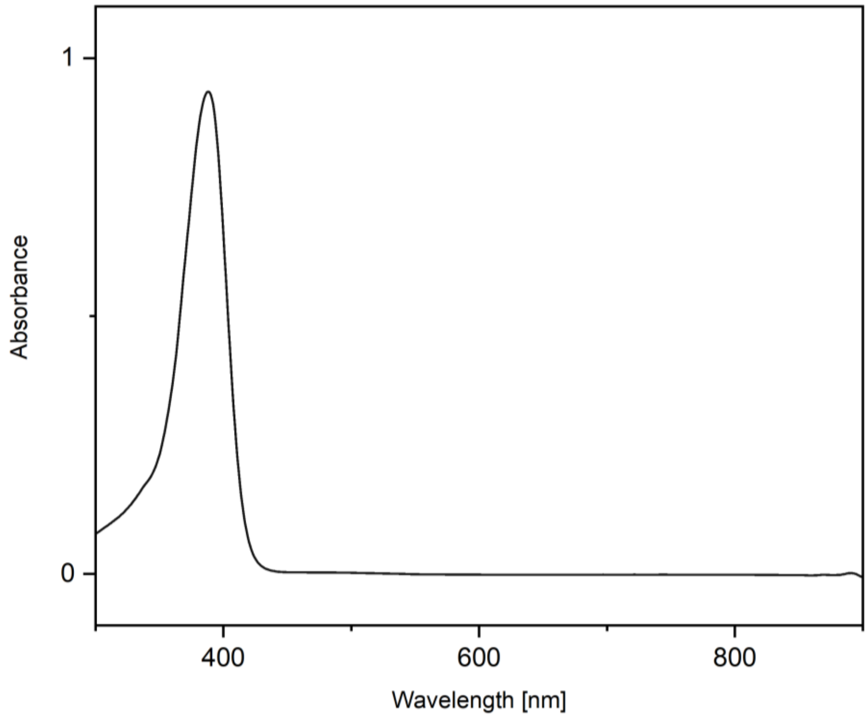

Figure S22. UV-vis spectrum of **2** in THF. λ_{max} = 388 nm.

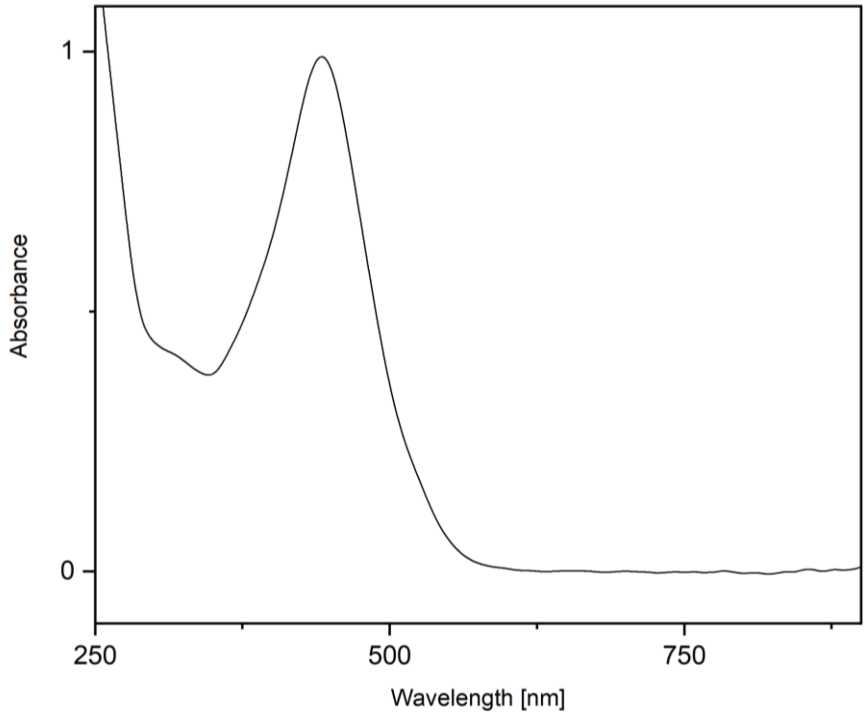

Figure S23. UV-vis spectrum of **3** in THF. λ_{max} = 443 nm.

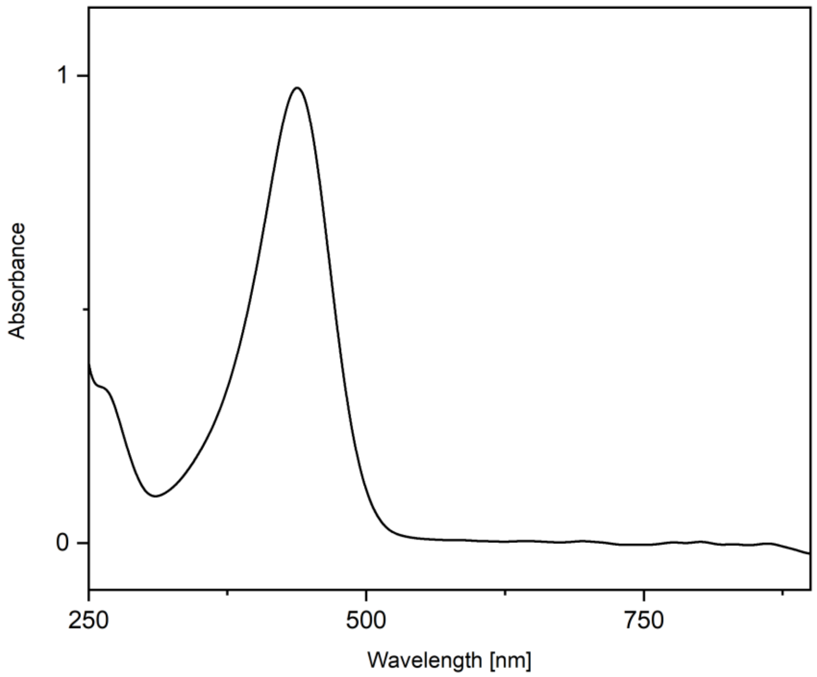

Figure S24. UV-vis spectrum of **4-K** in THF. λ_{max} = 438 nm.

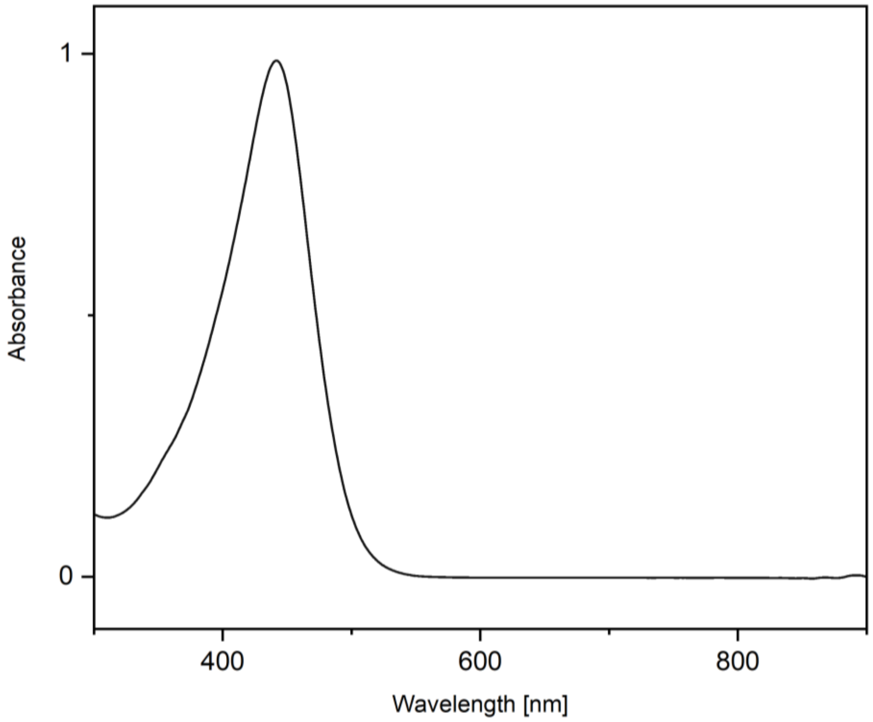

Figure S25. UV-vis spectrum of **4-H** in THF. λ_{max} = 441 nm.

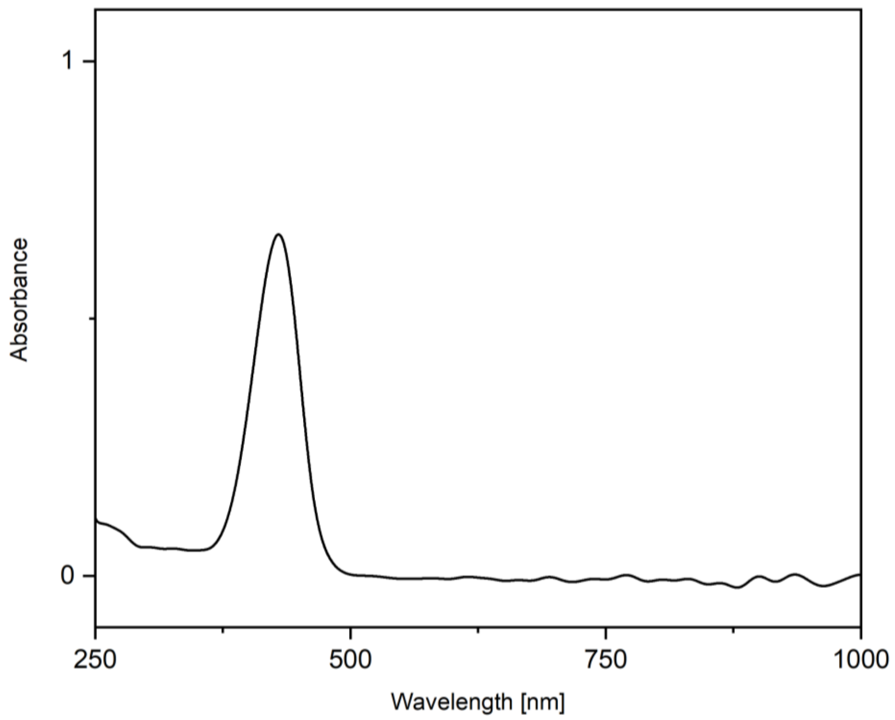

Figure S26. UV-vis spectrum of **5-H** in THF. λ_{max} = 430 nm.

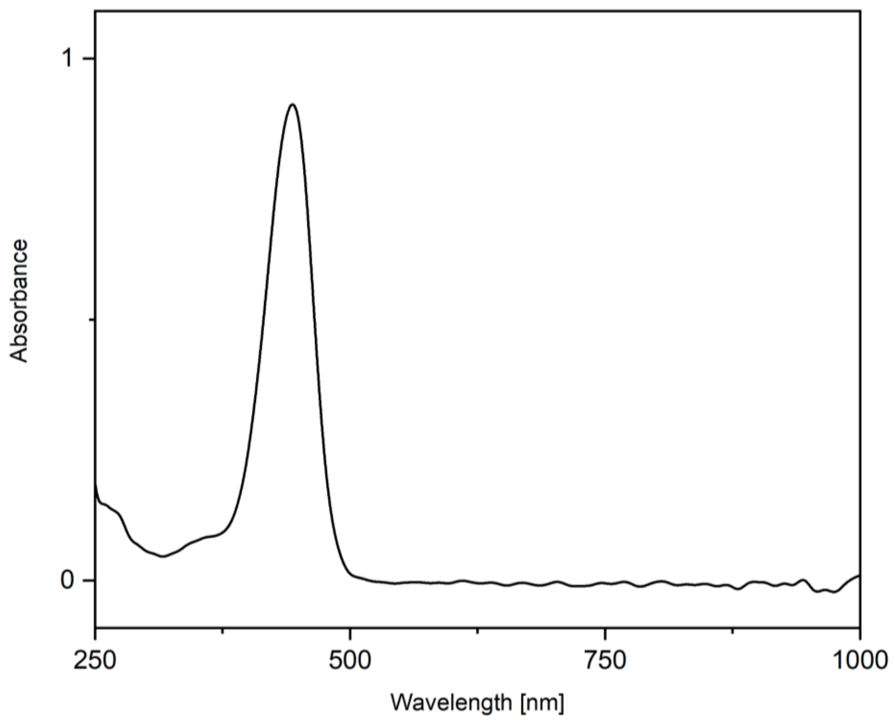

Figure S27. UV-vis spectrum of **5-Me** in THF. λ_{max} = 444 nm.

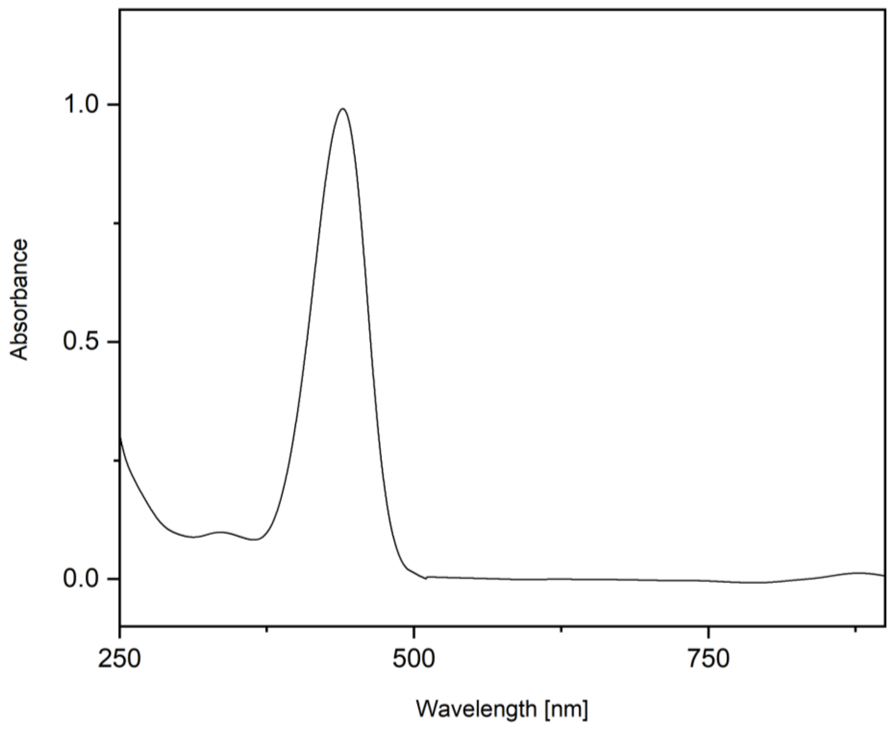

Figure S28. UV-vis spectrum of **5-TMS** in THF. λ_{max} = 440 nm.

IR spectra

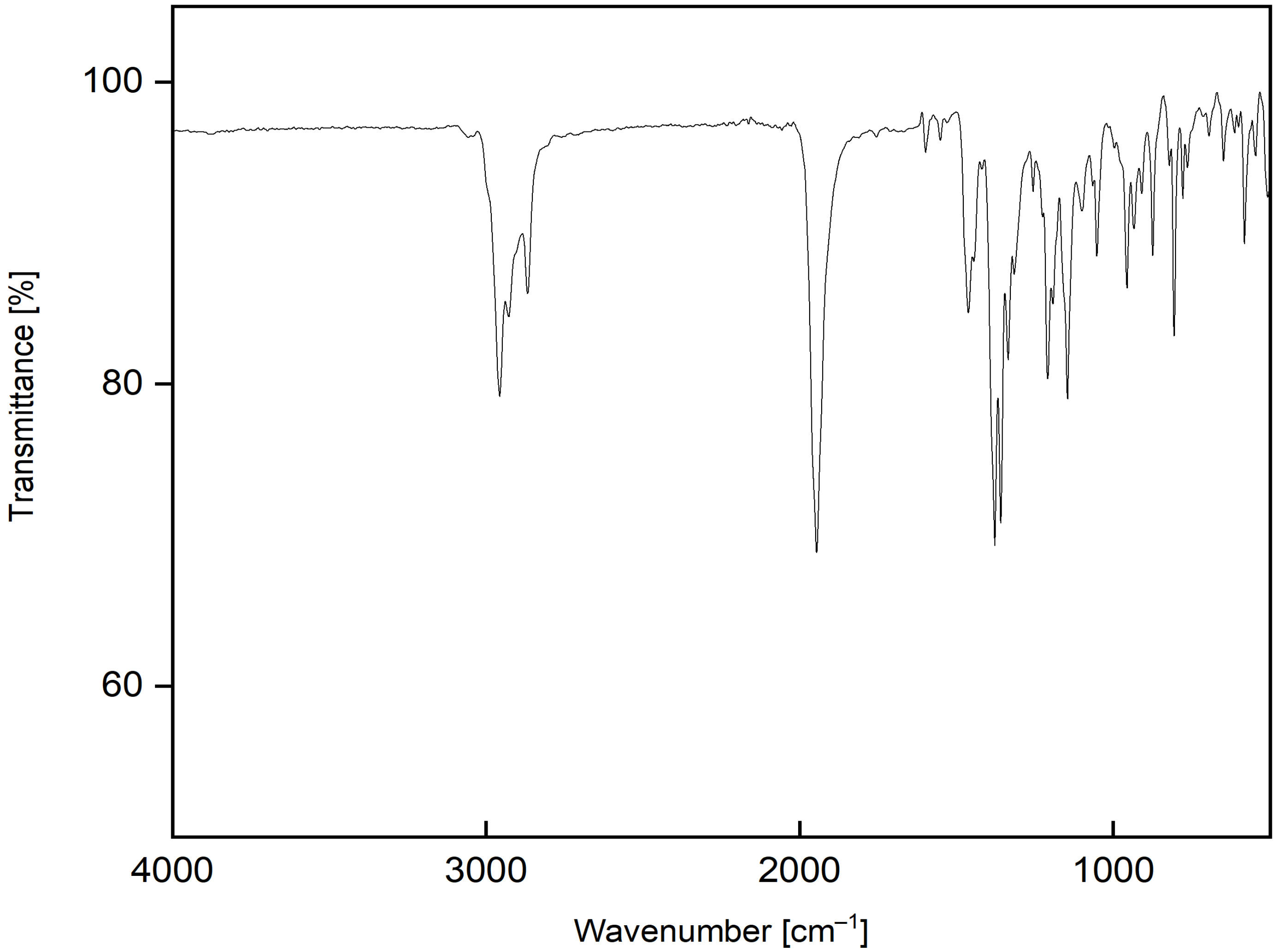

Figure S29. Solid-state IR spectrum of **2**.

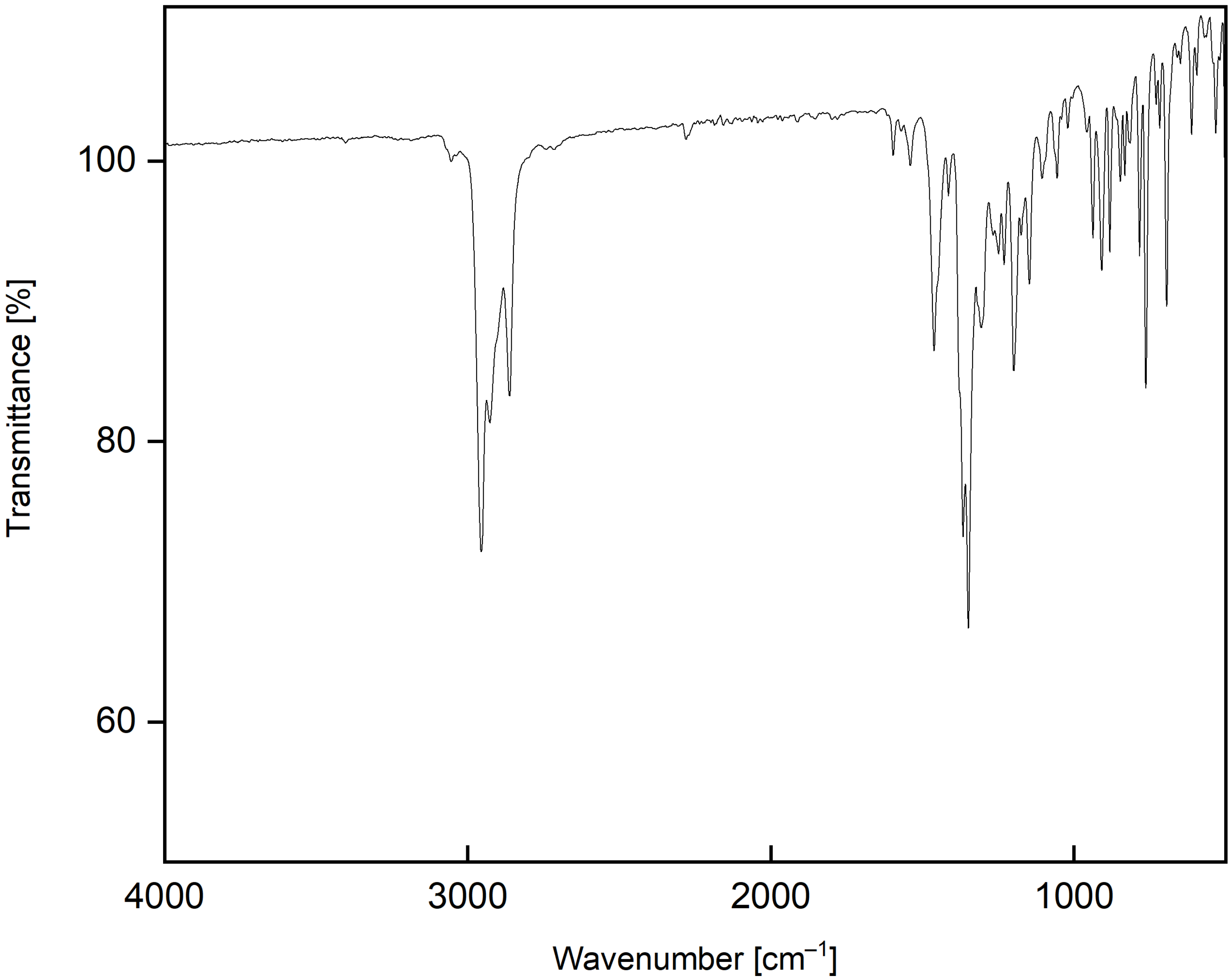

Figure S30. Solid-state IR spectrum of **3**.

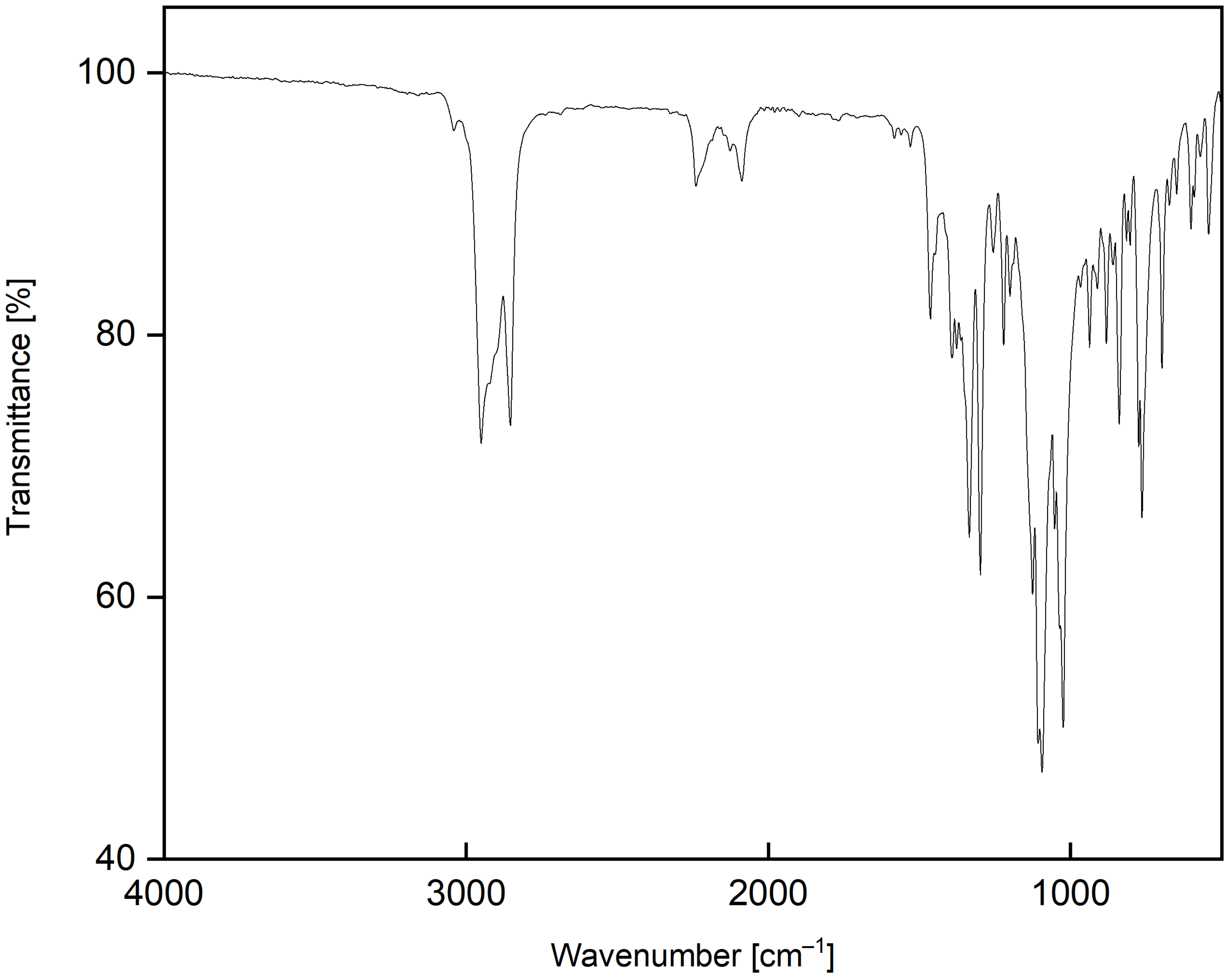

Figure S31. Solid-state IR spectrum of **4-K**.

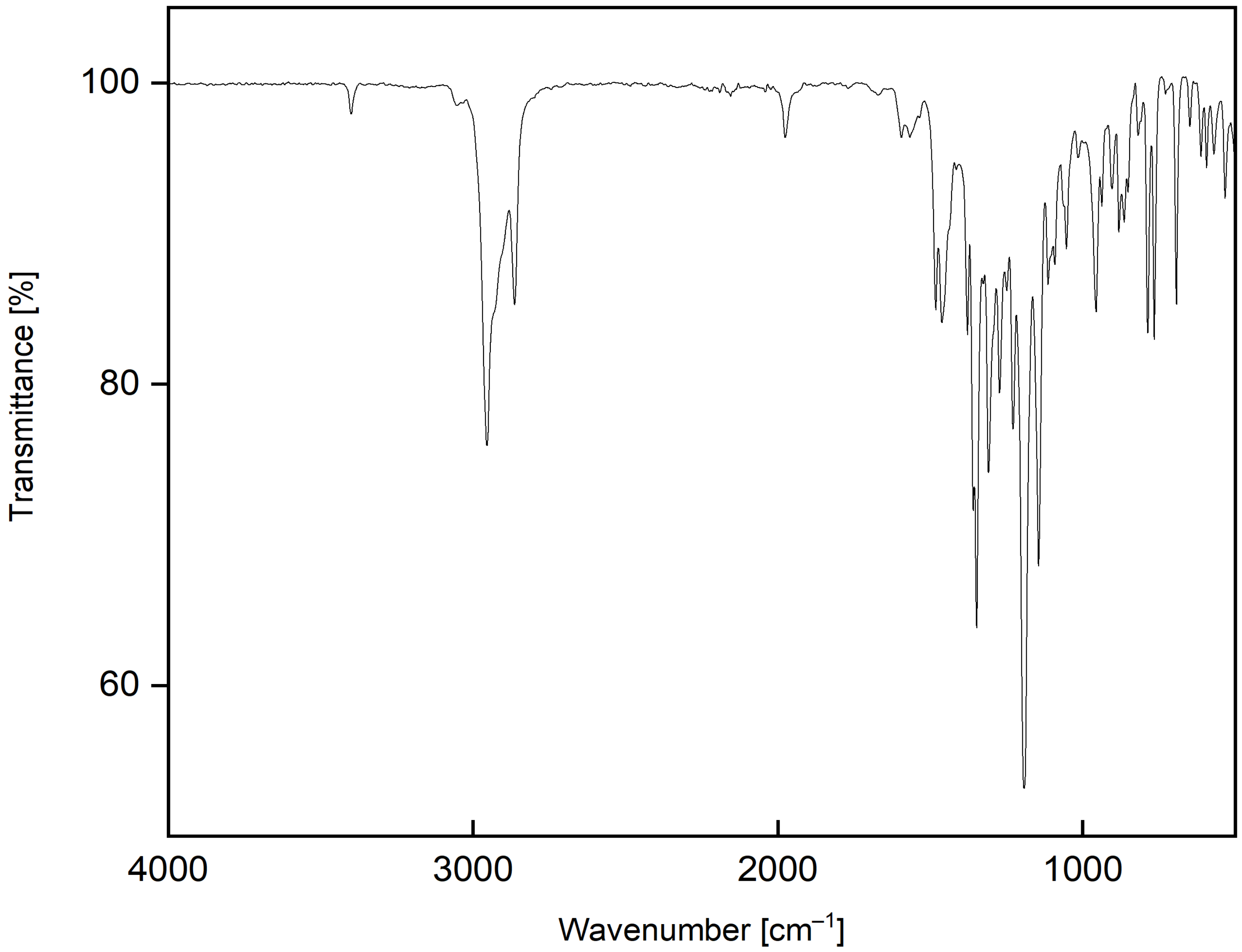

Figure S32. Solid-state IR spectrum of **4-H**.

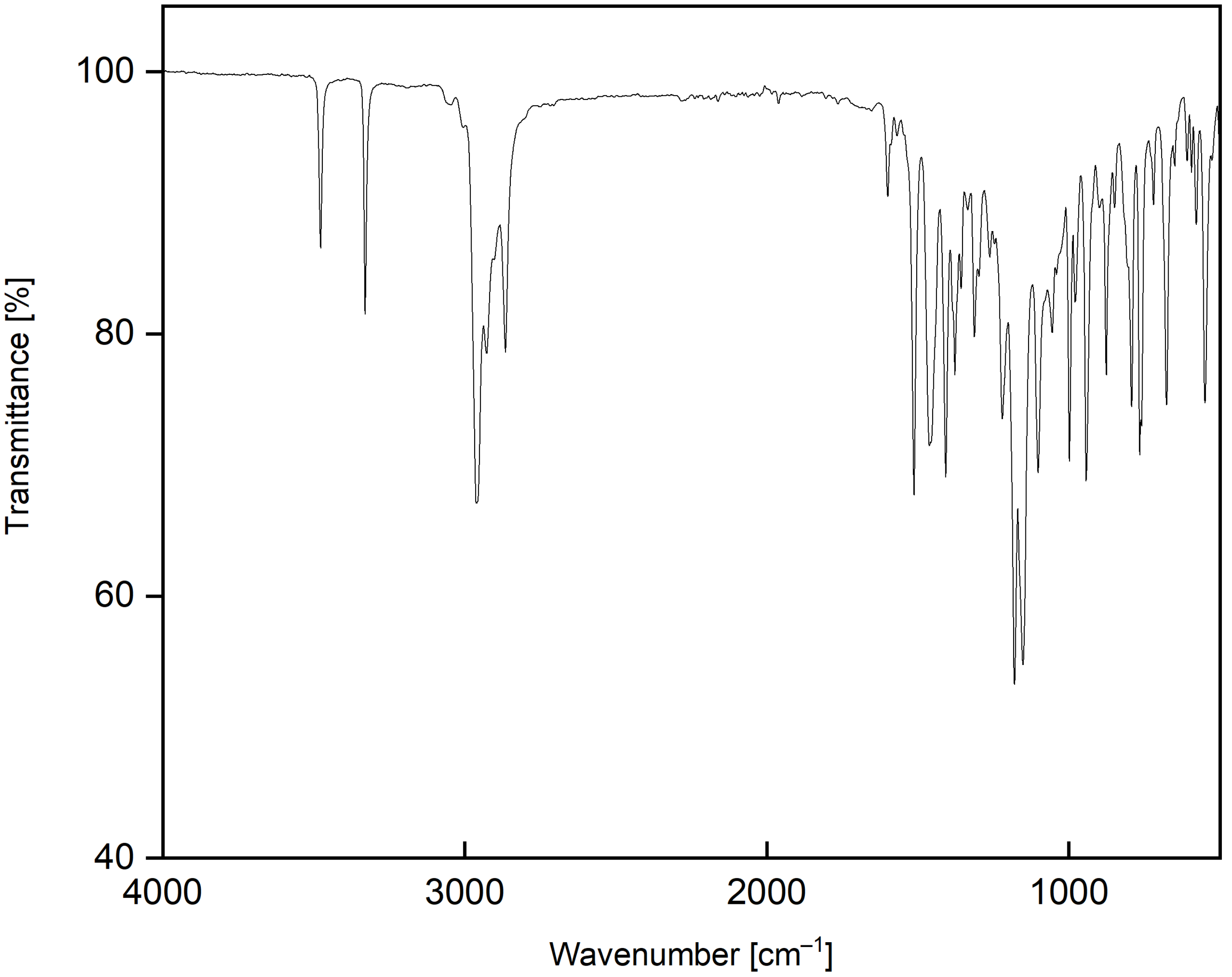

Figure S33. Solid-state IR spectrum of **5-H**.

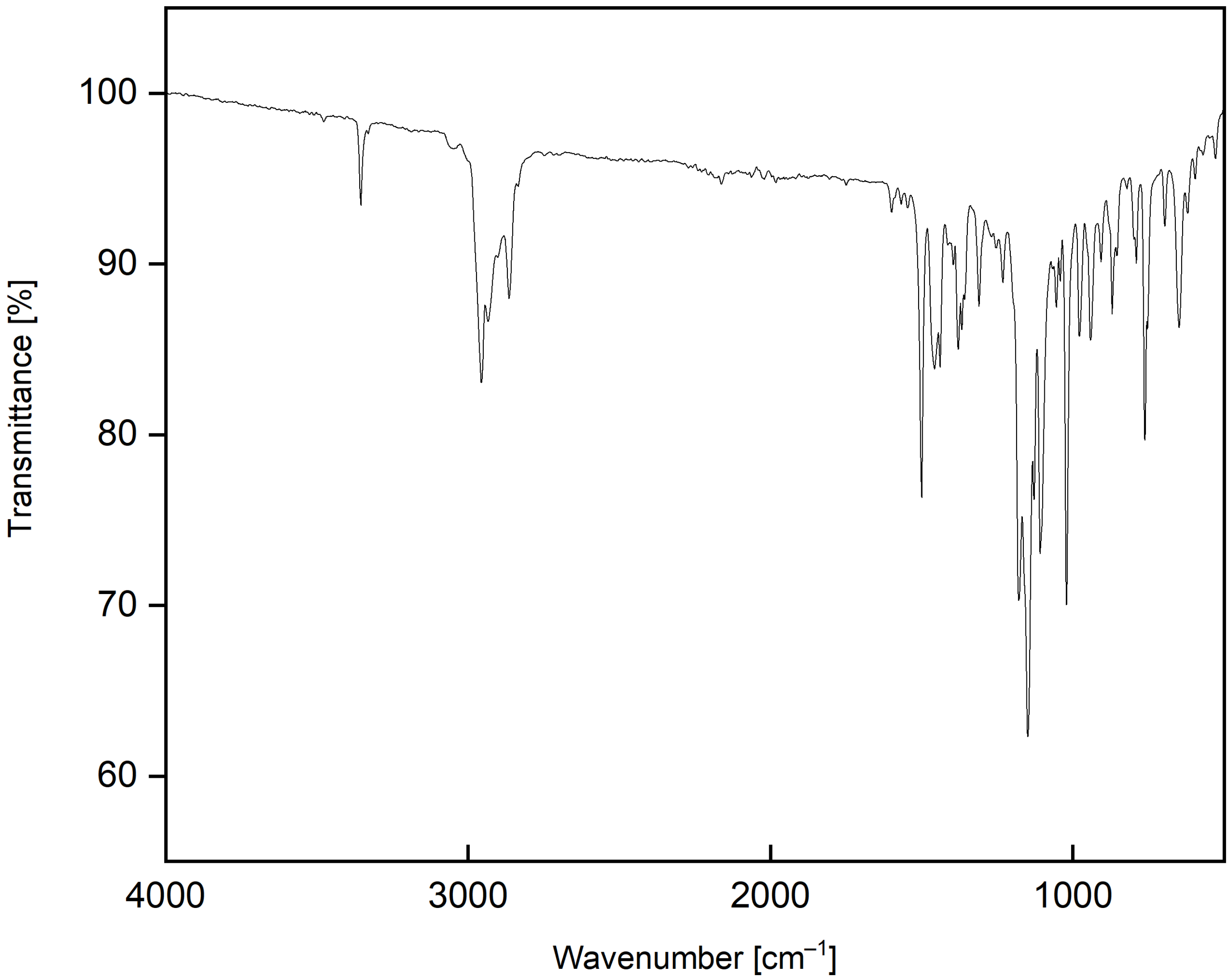

Figure S34. Solid-state IR spectrum of **5-Me**.

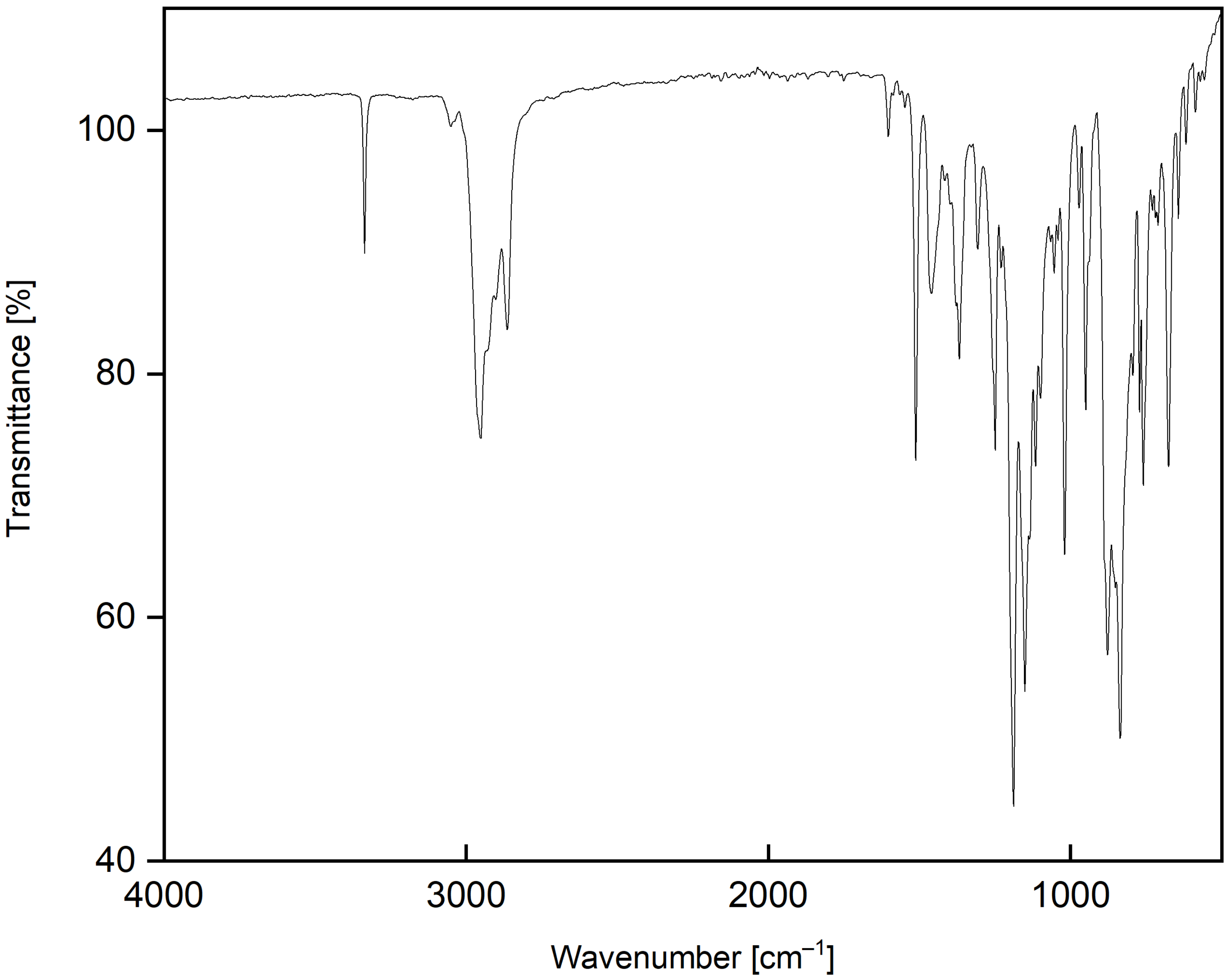

Figure S35. Solid-state IR spectrum of **5-TMS**.

Cyclic voltammetry

Cyclic voltammetry experiments were performed using a Gamry Instruments Reference 600 potentiostat. A standard three-electrode cell configuration was employed using a platinum disk working electrode, a platinum wire counter electrode, and a silver wire, separated by a *Vycor* tip, serving as the reference electrode. Formal redox potentials are referenced to the ferrocene/ferrocenium ($[Cp_2Fe]^{+/0}$) redox couple by using decamethylferrocene as an internal standard. Tetra-*n*-butylammonium hexafluorophosphate ($[n\text{Bu}_4\text{N}][\text{PF}_6]$) was employed as the supporting electrolyte. Compensation for resistive losses (*iR* drop) was employed for all measurements.

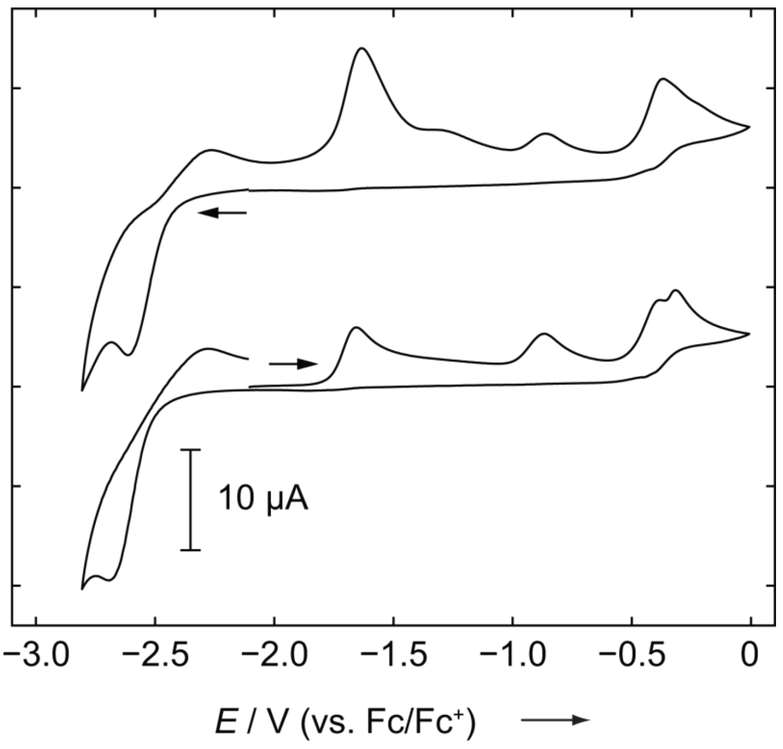

Figure S36. Cyclic voltammograms of **3** in THF/0.1 M $[n\text{Bu}_4\text{N}][\text{PF}_6]$ measured at 250 mV s^{-1}. Potential scans were initiated at the rest potential and are shown in the positive and negative direction. Formal potentials: $E_{pa1} = -1.67$ V, $E_{pa2} = -0.87$ V, $E_{pa3} = -0.40$ V, $E_{pa4} = -0.32$ V (oxidations), $E_{pc} = -2.66$ V (reduction, relative to the Fc/Fc$^+$ couple).

X-ray crystallographic data

The crystal data of **3·C_6H_6** were collected on a Bruker X8-APEX II diffractometer with a CCD area detector and multi-layer mirror monochromated $Mo_{K\alpha}$ radiation. The crystal data of **3·Et_2O**, **5-H** and **5-Me** were collected on a Bruker D8 Quest diffractometer with a CMOS area detector and multi-layer mirror monochromated $Mo_{K\alpha}$ radiation. The crystal data of **2**, **4-H**, **4-K** and **5-TMS** were collected on a XtaLAB Synergy, Dualflex diffractometer with a HyPix area detector and multi-layer mirror monochromated $Cu_{K\alpha}$ radiation. The structures were solved using the intrinsic phasing method,[3] refined with the ShelXL program[4] and expanded using Fourier techniques. All non-hydrogen atoms were refined anisotropically. Hydrogen atoms were included in structure factor calculations. All hydrogen atoms were assigned to idealized positions.

Crystallographic data have been deposited with the Cambridge Crystallographic Data Center as supplementary publication numbers CCDC 2036988–2036995 (**3·C_6H_6** 2036988; **4-H** 2036989; **3·Et_2O** 2036990; **5-Me** 2036991; **4-K** 2036992; **5-H** 2036993; **2** 2036994; **5-TMS** 2036995) These data can be obtained free of charge from The Cambridge Crystallographic Data Centre *via* www.ccdc.cam.ac.uk/data_request/cif.

Crystal data for 2: $C_{36}H_{54}BNO\cdot(C_5H_{12})_{0.5}$, M_r = 563.68, orange block, 0.200×0.145×0.104 mm^3, monolinic space group $P\ 2_1/n$, a = 48.8856(5) Å, b = 9.71906(6) Å, c = 16.77861(17) Å, β = 118.9826(13)°, V = 6973.55(14) Å^3, Z = 8, ρ_{calcd} = 1.074 g·cm^{-3}, μ = 0.461 mm^{-1}, F(000) = 2488, T = 100(2) K, R_1 = 0.0439, wR^2 = 0.1166, 13688 independent reflections [$2\theta \leq 72.128°$] and 778 parameters.

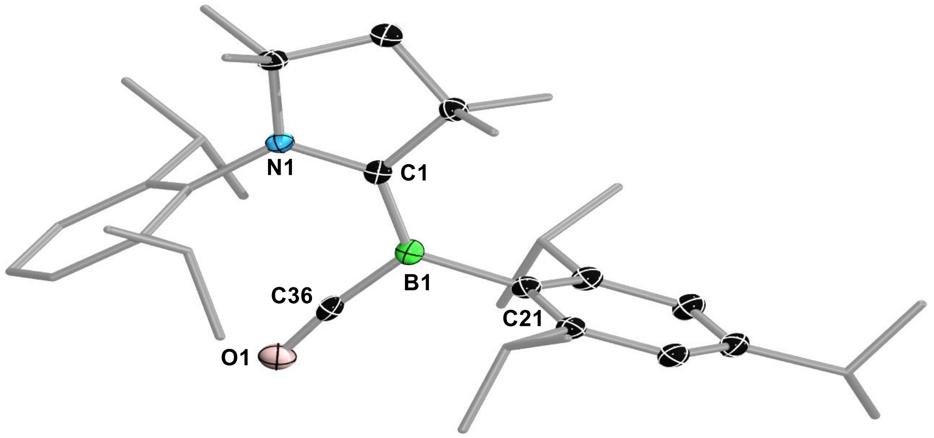

Figure S37. Crystallographically-derived molecular structure of **2** (one of the two molecules present in the asymmetric unit). Thermal ellipsoids set at 50% probability. Thermal ellipsoids of ligand periphery and hydrogen atoms omitted for clarity. Selected bond lengths (Å) and angles (°): N1–C1 1.3575(15), C1–B1 1.5047(17), B1–C36 1.4857(18), C36–O1 1.1461(15), C1-B1-C21 127.43(11), C21-B1-C36 112.28(10), C36-B1-C1 120.26(10), torsion angle (N1,C1,B1,C36) 2.93(19).

Refinement details for 3·Et₂O: The asymmetric unit contains half an Et_2O molecule positioned in the vicinity of an inversion center, and presenting an additional twofold flip-disorder in a 9:1 ratio. The 1,2 and 1,3 distances in these two molecules were equalized using SAME and ADPs were restrained with SIMU 0.01. The CAAC backbone was modelled as twofold disordered in C2 > C8 in a 4:6 ratio, with ADP restraints SIMU 0.005. The *i*Pr group C18 > C20 of the Dip substituent was modelled as twofold disordered in a 4:6 ratio, with ADP restraints SIMU 0.005.

Crystal data for 3·Et₂O: $C_{72}H_{108}B_2K_2N_2O_2\cdot C_4H_{10}O$. M_r = 598.73, red block, 0.325×0.285×0.245 mm^3, monoclinic space group $C2/c$, a = 19.583(10) Å, b = 18.863(3) Å, c = 20.396(6) Å, β = 94.926(15)°, V = 7506(4) Å^3, Z = 8, ρ_{calcd} = 1.069 g·cm^{-3}, μ = 0.170 mm^{-}

^{1}, $F(000) = 2640$, $T = 100(2)$ K, $R_1 = 0.0515$, $wR^2 = 0.1068$, 7389 independent reflections [$2\theta \leq 52.044°$] and 564 parameters.

Refinement details for 3·C$_6$H$_6$: The CAAC backbone was modelled as twofold disordered in C3 > C8 in a 1:3 ratio, with ADP restraints SIMU 0.008. The *i*Pr groups of the Dip substituent, C15 > C17 and C18 > C20 were modelled as twofold disordered in a 86:14 and 46:54 ratio, respectively, with ADP restraints SIMU 0.01 and SIMU 0.005, respectively.

Crystal data for 3·C$_6$H$_6$: $C_{72}H_{108}B_2K_2N_2O_2 \cdot C_6H_6$. $M_r = 1211.53$, red block, 0.327×0.30×0.214 mm^3, monoclinic space group $C2/c$, $a = 19.554(9)$ Å, $b = 18.666(7)$ Å, $c = 20.416(9)$ Å, $\beta = 95.249(13)°$, $V = 7421(5)$ Å^3, $Z = 4$, $\rho_{calcd} = 1.084$ g·cm^{-3}, $\mu = 0.172$ mm^{-1}, $F(000) = 2640$, $T = 100(2)$ K, $R_1 = 0.0578$, $wR^2 = 0.1093$, 6798 independent reflections [$2\theta \leq 51.026°$] and 521 parameters.

Refinement details for 4-H: All hydrogen atoms except H1 were assigned to idealized positions. The coordinate of H1 was refined freely. Highly disordered residual solvent in the asymmetric unit was removed using the Platon program Squeeze,[5] which allows for the mathematical compensation of the electron contribution of disordered solvent contained in the voids to the calculated diffraction intensities. A total of 168 electrons per unit cell were squeezed, i.e. ca. 4 molecules of the crystallization solvent pentane.

Crystal data for 4-H: $C_{72}H_{110}B_2K_2N_2O_2 \cdot$(squeezed solvent), $M_r = 1135.44$, orange block, 0.177×0.158×0.108 mm^3, trigonal space group $P\ 3_2\ 2\ 1$, $a = 21.1161(1)$ Å, $b = 21.1161(1)$ Å, $c = 14.8243(1)$ Å, $V = 5724.43(7)$ Å^3, $Z = 3$, $\rho_{calcd} = 0.988$ g·cm^{-3}, $\mu = 1.383$ mm^{-1}, $F(000) = 1860$, $T = 100(2)$ K, $R_1 = 0.0324$, $wR^2 = 0.0883$, 8081 independent reflections [$2\theta \leq 77.588°$] and 379 parameters.

Refinement details for 4-K: There were two large voids (each around 300 Å^3) in the cell. We could localize disordered THF molecules with multiple disorders, which could not be modelled satisfyingly. Highly disordered residual solvent in the asymmetric unit was removed using the Platon program Squeeze.[5] A total of 157 electrons were thus squeezed from the unit cell, i.e. 4 THF molecules. The *para*-*i*Pr groups of the Tip ligands were modelled as twofold disordered

in a 88:12 (RESI 71 and 72, C33 > C35) and 86:14 (RESI 73 and 74, C70 > C72) ratio, respectively. ADPs within these disorders were restrained using SIMU 0.005.

Crystal data for 4-K: $C_{88}H_{140}B_2K_4N_2O_2$·(squeezed solvent), M_r = 1500.05, red plate, 0.262× 0.074× 0.033 mm^3, triclinic space group $P\bar{1}$, a = 10.5087(1) Å, b = 21.0103(2) Å, c = 23.9386(3) Å, α = 67.840(1)°, β = 88.773(1)°, γ = 88.588(1)°, V = 4893.11(10) Å^3, Z = 2, ρ_{calcd} = 1.018 g·cm^{-3}, μ = 1.956 mm^{-1}, F(000) = 1632, T = 100(2) K, R_1 = 0.0480, wR^2 = 0.1187, 17843 independent reflections [$2\theta \leq 68.247°$] and 1007 parameters.

Refinement details for 5-H: All hydrogen atoms except H1 were assigned to idealized positions. The coordinates of H1 were refined freely. The molecule is situated on a mirror plane, with the planar BC_2O core occupying, and the aryl groups perpendicular to, the mirror plane. The ADPs of the disordered *i*Pr group C30 > C32 were restrained using SIMU.

Crystal data for 5-H: $C_{36}H_{56}BNO$, M_r = 529.62, yellow block, 0.343×0.265×0.215 mm^3, orthorhombic space group $Cmc2_1$, a = 16.342(3) Å, b = 9.9713(18) Å, c = 19.790(4) Å, V = 3224.9(10) Å^3, Z = 4, ρ_{calcd} = 1.091 g·cm^{-3}, μ = 0.063 mm^{-1}, F(000) = 1168, T = 100(2) K, R_1 = 0.0377, wR^2 = 0.0987, 3276 independent reflections [$2\theta \leq 52.036°$] and 232 parameters.

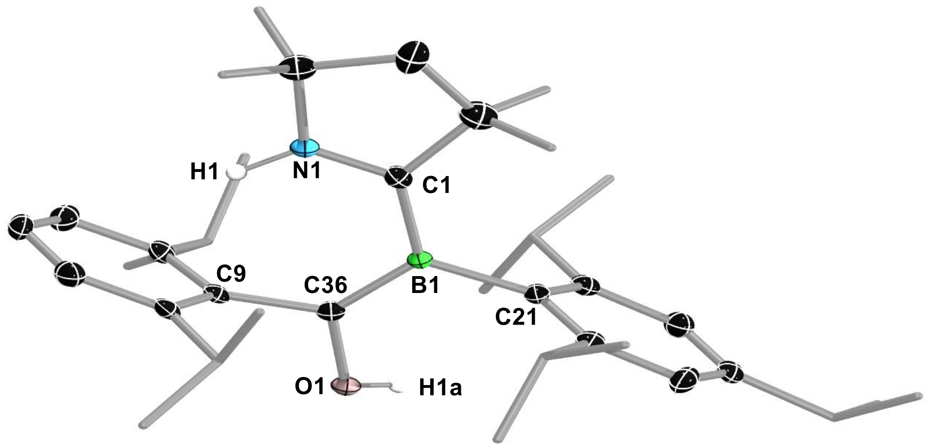

Figure S38. Crystallographically-derived molecular structure of **5-H**. Thermal ellipsoids set at 50% probability. Thermal ellipsoids of ligand periphery and hydrogen atoms omitted for clarity, except for the N- and O-bound hydrogen atoms. Selected bond lengths (Å) and angles (°): N1–C1 1.325(4), C1–B1 1.552(4), B1–C36 1.471(4), C36–O1 1.392(3), Σ(∠B1) 360.0(3), torsion angle (N1,C1,B1,C36) 0.000(2).

Refinement details for 5-Me: All hydrogen atoms except H1_1 and H1_2 were assigned to idealized positions. The coordinates of H1_1 and H1_2 were refined freely. The asymmetric unit contains two molecules of the compounds, one of which (RESI 2 MOL) presents a twofold disorder in the CAAC ligand backbone (RESI 31 and 32 CAAC: C2 > C8), refined to a 18:82 ratio, and in the iPr group C30 > C32 (RESI 41 and 42 iPr), refined to a 14:86 ratio. ADPs within these disorders were restrained using SIMU 0.01.

Crystal data for 5-Me: $C_{37}H_{58}BNO$, M_r = 543.65, yellow block, 0.293×0.211×0.144 mm^3, triclinic space group $P\bar{1}$, a = 14.448(4) Å, b = 14.453(3) Å, c = 16.639(3) Å, α = 88.629(12)°, β = 88.869(8)°, γ = 89.508(8)°, V = 3472.6(13) Å^3, Z = 4, ρ_{calcd} = 1.040 g·cm^{-3}, μ = 0.060 mm^{-1}, F(000) = 1200, T = 100(2) K, R_1 = 0.0638, wR^2 = 0.1400, 13666 independent reflections [$2\theta \leq 52.044°$] and 853 parameters.

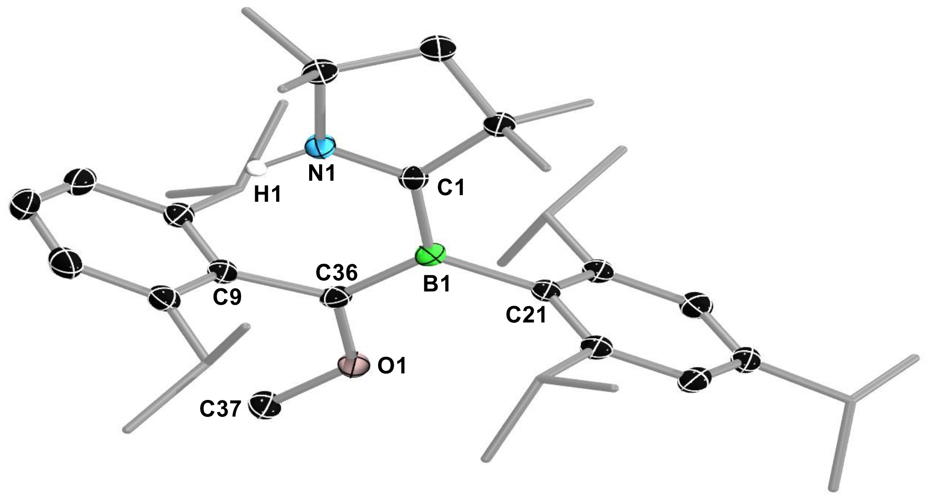

Figure S39. Crystallographically-derived molecular structure of **5-Me** (one of the two molecules present in the asymmetric unit). Thermal ellipsoids set at 50% probability. Thermal ellipsoids of ligand periphery and hydrogen atoms omitted for clarity, except for the N-bound hydrogen atom. Selected bond lengths (Å) and angles (°): N1–C1 1.337(2), C1–B1 1.546(2), B1–C36 1.484(2), C36–O1 1.3858(18), Σ(∠B1) 359.98(14), torsion angle (N1,C1,B1,C36) 3.7(2).

Refinement details for 5-TMS: Three reflections affected by the beamstop were omitted.

Crystal data for 5-TMS: $C_{39}H_{64}BNOSi$, M_r = 601.81, yellow block, 0.146×0.109×0.079 mm^3, orthorhombic space group $P\,c\,a\,2_1$, a = 20.47380(10) Å, b = 9.84080(10) Å, c = 18.83360(10)Å, V = 3793.87(3) Å^3, Z = 4, ρ_{calcd} = 1.054 g·cm^{-3},

μ = 0.742 mm^{-1}, F(000) = 1328, T = 100(2) K, R_1 = 0.0298, wR^2 = 0.0707, 7455 independent reflections [2θ≤72.130°] and 409 parameters.

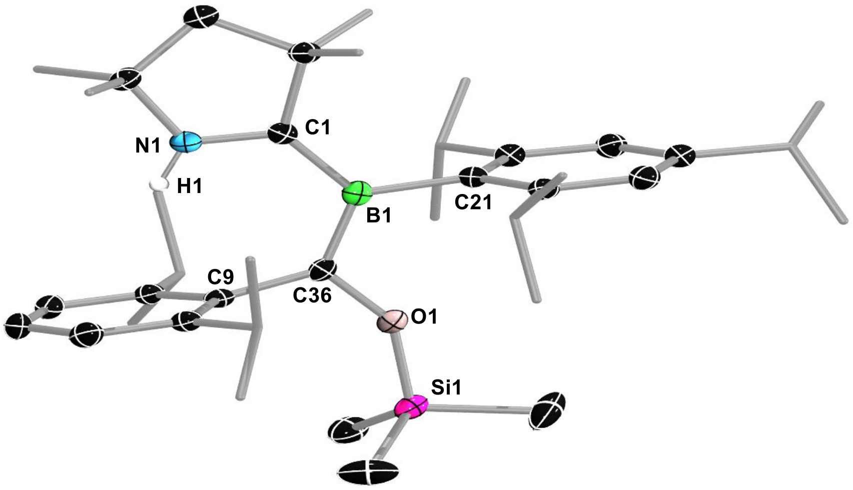

Figure S40. Crystallographically-derived molecular structure of **5-TMS**. Thermal ellipsoids set at 50% probability. Thermal ellipsoids of ligand periphery and hydrogen atoms omitted for clarity, except for the N-bound hydrogen atom. Selected bond lengths (Å) and angles (°): N1–C1 1.325(2), C1–B1 1.550(2), B1–C36 1.481(3), C36–O1 1.380(2), Σ(∠B1) 360.00(15), torsion angle (N1,C1,B1,C36) –1.7(3).

Computational details

Geometry optimizations and Hessian calculations were performed in the gas phase for **2**, **3** and **4-H** at the B3LYP[6-9]-D3[10](BJ)[11]/def2-SVP[12] level of theory considering restricted and unrestricted wavefunctions and singlet and triplet multiplicities, and [**2**]$^{\bullet-}$ at the B3LYP-D3(BJ)/6-31++G**[13-17] (doublet multiplicity). All optimized structures were characterized as minimum energy geometries as only positive eigenvalues were obtained in the vibrational frequency calculations. Additional geometry optimization calculations considering solvent effects using the SMD[18] model (solvent = THF, ε = 7.4257) were done for **4-K**. Stability tests[19,20] on the restricted and unrestricted wavefunctions were also performed, and contributed to the open-shell singlet attribution for **3**. Spin density plots and atomic charges were obtained within the Mulliken Population Analysis (MPA).[21] The atomic polar tensor (ATP)[22] charge analysis was also used, and the results were similar to those obtained with MPA. Mayer bond orders (MBOs)[23,24] were also calculated for selected systems. Additional high-level, multireference calculations at the CASSCF[25] and NEVPT2[26-28] levels of theory were performed for truncated models of **3** (namely **3a** and **3b**, see Fig. S37) considering singlet and triplet multiplicities, in order to confirm the energetic trends obtained at the DFT level. For the CASSCF calculations, we considered active spaces of two electrons and two orbitals, CASSCF(2,2), and four electrons and four orbitals, CASSCF(4,4), with calculations performed using the def2-SVP basis set. The resolution of identity approximation for Coulomb integrals (RI-J)[29] in combination with the numerical chain-of-sphere integration for the Hartree-Fock exchange integrals (COSX)[30] was used in the multireference calculations. The biradical character (y_0) of **3** was obtained using the Yamaguchi formula:[31-33]

$$y_i = 1 - \frac{2T_i}{1 + {T_i}^2} \qquad (S1)$$

where T_i is calculated from the occupation numbers (ON) of the $HOMO - i$ and $LUMO + i$ natural orbitals of a CASSCF calculation from the following expression:

$$T_i = \frac{ON_{HOMO-i} - ON_{LUMO+i}}{2} \qquad (S2)$$

For y_0, eq. S1 is reduced to:

$$y_0 = 1 - \frac{2T_0}{1 + {T_0}^2} \qquad (S3)$$

where T_0 is given by:

$$T_0 = \frac{ON_{HOMO} - ON_{LUMO}}{2} \qquad (S4)$$

Finally, natural bond order (NBO)[34] calculations in terms of the "different hybrids for different spins" approach[35] were performed for the ketyl radical anion $[\mathbf{3}]^{\bullet-}$ at the UB3LYP-D3(BJ)/6-31++G**[13-17] level of theory. The NBO calculations were performed using the NBO 7.0 program.[36] Multireference calculations were performed in Orca 4.1.1.[37] MBOs were obtained using Multiwfn 3.7.[38] All other calculations were performed using Gaussian 16, Revision B.01.[39]

Table S1. Zero-point-energy-corrected electronic energies at B3LYP-D3(BJ)/def2-SVP of **3** and **4-H** considering closed-shell singlet, open-shell singlet and triplet multiplicities. For **4-H**, CS = OS.

	**3**	**4-H**
E(CS) (hartree)	–4314.892517	–4316.118728
E(OS) (hartree)	–4314.925644	-
E(TS) (hartree)	–4314.925612	–4316.051674
HL-gap(CS) (eV)	0.52	3.05
ΔE(OS-CS) (kJ/mol)	–87	-
ΔE(TS-OS) (kJ/mol)	0.08	176

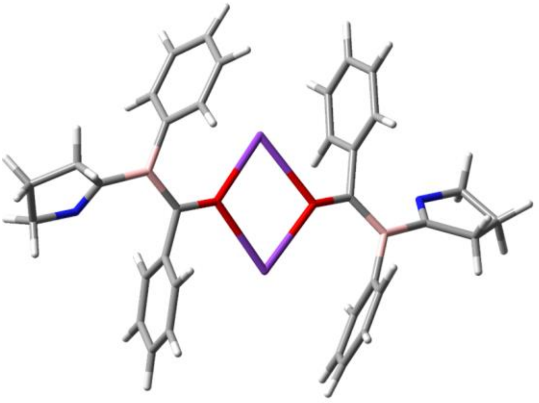

3a

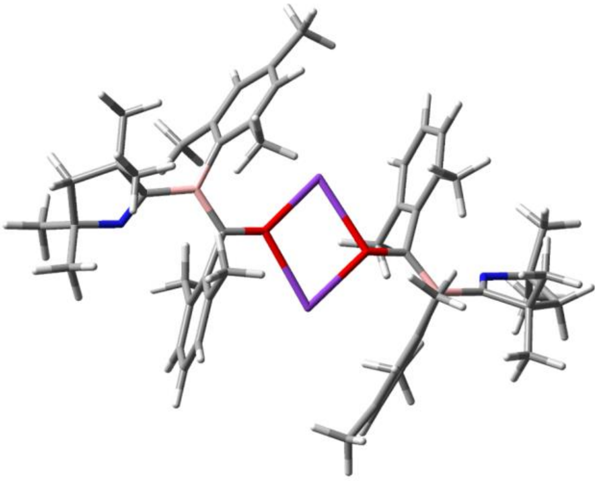

3b

Figure S41. Truncated models of **3** for the CASSCF/NEVPT2 calculations.

Tables S2-S4 give a characterization of the CASSCF wave functions (energies, weights of the most important configurations, occupation numbers, orbital energies, etc). The weights are the sum of the squares of the CI coefficients for all configuration state functions (CSFs) that belong to a particular configuration. For example, the configuration 2 0 of **3a** (Table S2) has a weight of 0.51172, which means that the singlet ground state is composed of 51.2% of this configuration, while the configuration 0 2 of **3a** has a weight of 0.48828 (48.8%).

**Table S2.** NEVPT2/CASSCF(2,2) results for **3a** using the def2-SVP basis set.	
**CASSCF(2,2)/def2-SVP**	
Electronic energy (singlet), E_h	-2811.204947
Electronic energy (triplet), E_h	-2811.204929
Singlet-triplet gap ($\Delta_{T-S} = E_{triplet} - E_{singlet}$, kJ/mol)	0.05
Weight of the 2 0 configuration (singlet)	0.51172
Weight of the 0 2 configuration (singlet)	0.48828
ON_{HOMO}, singlet (SOMO1)	1.0234
ON_{LUMO}, singlet (SOMO2)	0.9766
H-L gap (eV)	0.14
T_0, singlet	0.0234
y_0, singlet	0.953 (95.3%)
**NEVPT2/CASSCF(2,2)/def2-SVP**	
Electronic energy (singlet), E_h	-2816.749084
Electronic energy (triplet), E_h	-2816.7490475
Singlet-triplet gap ($\Delta_{T-S} = E_{triplet} - E_{singlet}$, kJ/mol)	0.10

**Table S3.** NEVPT2/CASSCF(4,4) results for **3a** using the def2-SVP basis set.	
**CASSCF(4,4)/def2-SVP**	
Electronic energy (singlet), E_h	-2811.207142
Electronic energy (triplet), E_h	-2811.207124
Singlet-triplet gap ($\Delta_{T-S} = E_{triplet} - E_{singlet}$, kJ/mol)	0.05
Weight of the 2 2 0 0 configuration (singlet)	0.51020
Weight of the 2 0 2 0 configuration (singlet)	0.48679
ON_{HOMO}, singlet (SOMO1)	1.0235
ON_{LUMO}, singlet (SOMO2)	0.9765
H-L gap (eV)	0.14
T_0, singlet	0.0235
y_0, singlet	0.953 (95.3%)
**NEVPT2/CASSCF(4,4)/def2-SVP**	
Electronic energy (singlet), E_h	-2816.751591
Electronic energy (triplet), E_h	-2816.751454
Singlet-triplet gap ($\Delta_{T-S} = E_{triplet} - E_{singlet}$, kJ/mol)	0.36

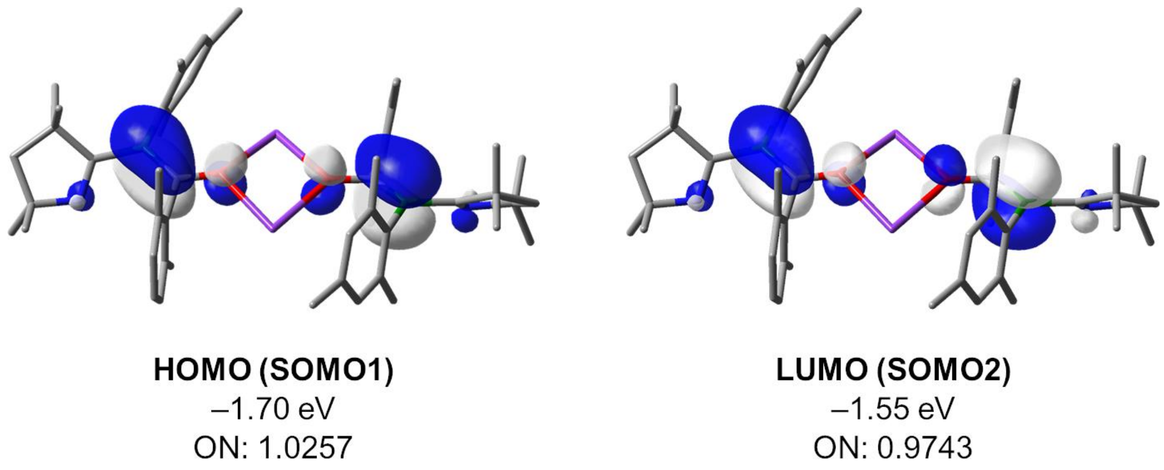

Figure S42. Active space orbitals of **3b** at the CASSCF(2,2)/def2-SVP level of theory, their energies (eV) and occupation numbers (ON).

**Table S4.** NEVPT2/CASSCF(2,2) results for **3b** using the def2-SVP basis set.	
**CASSCF(2,2)/def2-SVP**	
Electronic energy (singlet), E_h	-3513.313736
Electronic energy (triplet), E_h	-3513.313712
Singlet-triplet gap ($\Delta_{T-S} = E_{triplet} - E_{singlet}$, kJ/mol)	0.06
Weight of the 2 0 configuration (singlet)	0.51283
Weight of the 0 2 configuration (singlet)	0.48717
ON_{HOMO}, singlet (SOMO1)	1.0257
ON_{LUMO}, singlet (SOMO2)	0.9743
H-L gap (eV)	0.15
T_0, singlet	0.0257
y_0, singlet	0.949 (94.9%)
**NEVPT2/CASSCF(2,2)/def2-SVP**	
Electronic energy (singlet), E_h	-3521.624000
Electronic energy (triplet), E_h	-3521.623952
Singlet-triplet gap ($\Delta_{T-S} = E_{triplet} - E_{singlet}$, kJ/mol)	0.13

For a system where two electrons are distributed in two molecular orbitals ϕ_1 and ϕ_2, six determinants can be generated – three of them form the triplet manifold ($^3|\phi_1\phi_2\rangle$) and the remaining ones are combined to give rise to three distinct singlet states (which, according to their spatial forms, can be abbreviated as $^1|\phi_1\phi_2\rangle$, $^1|\phi_1^2 - \phi_2^2\rangle$ and $^1|\phi_1^2 + \phi_2^2\rangle$). The order of these states depends on the shape of the orbitals: if they are localized in the distinct spin centers, $^1|\phi_1\phi_2\rangle$ lies below $^1|\phi_1^2 - \phi_2^2\rangle$, while the latter state is lower in energy for a set of delocalized orbitals (the $^1|\phi_1^2 + \phi_2^2\rangle$ state is the highest lying for both cases; for more details, see the reviews of Bonačić-Koutecký, Koutecký and Michl[40,41]). If the ϕ_1 and ϕ_2 orbitals are degenerate and the singlet states do not interact, the triplet state is expected to be the lowest energy state. However, if ϕ_1 becomes more stable than ϕ_2, the states $^1|\phi_1^2 - \phi_2^2\rangle$ and $^1|\phi_1^2 + \phi_2^2\rangle$ start to mix. For strong couplings we obtain the closed-shell situation, in which the lowest lying state is $^1|\phi_1^2\rangle$. Since ϕ_1 is considerably lower in energy than ϕ_2 for strong interactions, this state is lower in energy than $^3|\phi_1\phi_2\rangle$.

For smaller mixings the wave functions can be described as $^1|c_1\phi_1^2 - c_2\phi_2^2\rangle$ and $^1|c_1\phi_1^2 + c_2\phi_2^2\rangle$, with $c_1 \neq c_2$. With increasing interaction, the difference between c_1 and c_2

increases. Whether the singlet $^1|c_1\phi_1^2 - c_2\phi_2^2\rangle$ becomes lower than the triplet $^3|\phi_1\phi_2\rangle$ or not will depend on the interaction. Dynamic correlation effects will also stabilize the singlet state with respect to the triplet state because two electrons with the same spin (triplet state) already avoid each other due to the antisymmetrization of the wave function (Pauli principle). Consequently, dynamic correlation effects for the triplet are expected to be smaller.

By inspecting Tables S2–S4 and Figure S42, it becomes obvious that the weights of the ground and excited configurations are not completely degenerate. This is a direct consequence of the small interaction between both spin centers. Due to this interaction, the HOMO and LUMO (or ϕ_1 and ϕ_2) of the CASSCF calculations are not degenerate, and thus their occupation numbers are not identical. Tables S2-S4 also indicate that dynamic correlation effects, which are present in NEVPT2 but neglected in CASSCF(2,2) or CASSCF(4,4), increases the S-T gap, i. e. lowers the singlet state slightly more than the triplet state. As the absence of interaction between the spin centers would lead to a triplet ground state, the CASSCF calculations evidence that the spin centers have a small but non-negligible interaction, which ultimately leads to a preferred open-shell singlet state.

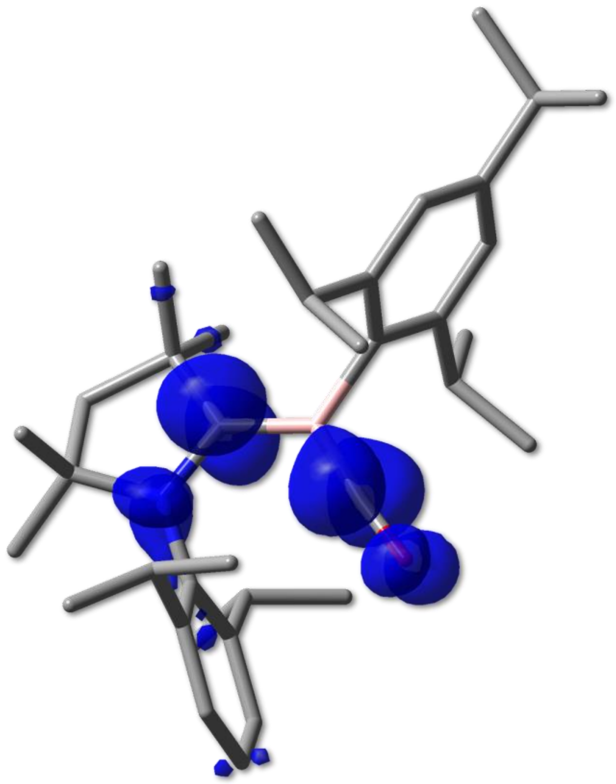

Figure S41. Plot of Mulliken spin density of the intermediate borylene radical anion **[2]**$^{\bullet-}$ (UB3LYP-D3(BJ)/6-31++G**). Spin density distribution: N1 +0.03, C1 +0.64, B1 –0.09, C36 +0.38, O1 +0.10.

Table S5. Second order perturbation theory analysis of [**3**]$^{•-}$ (α system). Only terms above 1.5 kcal/mol are shown.

Donor NBO	Acceptor NBO	E(2)	
		kcal/mol	kJ/mol
40. LP (1) O 1	148. BD*(1) C 2- B 3	2.5	10.5
40. LP (1) O 1	264. RY (1) C 2	4.1	17.0
41. LP (2) O 1	148. BD*(1) C 2- B 3	3.7	15.3
41. LP (2) O 1	150. BD*(1) C 2- C 4	7.8	32.5
42. LP (3) O 1	149. BD*(2) C 2- B 3	23.2	97.0
43. LP (1) N 72	228. BD*(1) C 71- C 76	4.6	19.1
43. LP (1) N 72	233. BD*(1) C 73- C 77	2.7	11.4
43. LP (1) N 72	853. RY (1) C 71	2.6	10.9
46. BD (2) C 2- B 3	149. BD*(2) C 2- B 3	4.3	18.2
46. BD (2) C 2- B 3	153. BD*(1) C 4- C 5	1.7	7.2
46. BD (2) C 2- B 3	155. BD*(1) C 4- C 12	2.0	8.3
46. BD (2) C 2- B 3	167. BD*(1) C 13- C 14	2.4	9.9
46. BD (2) C 2- B 3	169. BD*(1) C 13- C 20	1.9	7.9
46. BD (2) C 2- B 3	227. BD*(2) C 71- N 72	7.6	31.6
46. BD (2) C 2- B 3	250. RY (1) O 1	1.7	7.1
47. BD (1) C 2- C 4	164. BD*(1) C 10- C 12	1.5	6.3
48. BD (1) B 3- C 13	150. BD*(1) C 2- C 4	1.8	7.4
48. BD (1) B 3- C 13	170. BD*(1) C 14- C 15	2.5	10.3
48. BD (1) B 3- C 13	178. BD*(1) C 18- C 20	2.3	9.7
49. BD (1) B 3- C 71	147. BD*(1) O 1- C 2	1.6	6.7
49. BD (1) B 3- C 71	229. BD*(1) N 72- C 77	2.6	10.7
51. BD (2) C 4- C 5	147. BD*(1) O 1- C 2	2.2	9.2
51. BD (2) C 4- C 5	160. BD*(2) C 6- C 8	11.8	49.5
51. BD (2) C 4- C 5	165. BD*(2) C 10- C 12	10.7	44.6
51. BD (2) C 4- C 5	191. BD*(1) C 31- C 33	2.0	8.2
53. BD (1) C 5- C 6	150. BD*(1) C 2- C 4	1.5	6.4
55. BD (1) C 6- H 7	153. BD*(1) C 4- C 5	2.2	9.1
55. BD (1) C 6- H 7	162. BD*(1) C 8- C 10	1.7	7.2
57. BD (2) C 6- C 8	154. BD*(2) C 4- C 5	8.8	36.8

57. BD (2) C 6- C 8	165. BD*(2) C 10- C 12	8.8	36.9
58. BD (1) C 8- H 9	156. BD*(1) C 5- C 6	1.7	7.1
58. BD (1) C 8- H 9	164. BD*(1) C 10- C 12	1.7	7.0
60. BD (1) C 10- H 11	155. BD*(1) C 4- C 12	2.2	9.4
60. BD (1) C 10- H 11	159. BD*(1) C 6- C 8	1.7	7.1
62. BD (2) C 10- C 12	154. BD*(2) C 4- C 5	9.3	39.0
62. BD (2) C 10- C 12	160. BD*(2) C 6- C 8	11.3	47.4
64. BD (1) C 13- C 14	151. BD*(1) B 3- C 13	1.6	6.9
64. BD (1) C 13- C 14	180. BD*(1) C 20- C 61	1.5	6.3
65. BD (2) C 13- C 14	148. BD*(1) C 2- B 3	1.6	6.9
65. BD (2) C 13- C 14	152. BD*(1) B 3- C 71	1.5	6.4
65. BD (2) C 13- C 14	174. BD*(2) C 15- C 17	11.4	47.7
65. BD (2) C 13- C 14	179. BD*(2) C 18- C 20	11.3	47.2
65. BD (2) C 13- C 14	201. BD*(1) C 41- C 47	1.8	7.4
66. BD (1) C 13- C 20	151. BD*(1) B 3- C 13	1.5	6.4
69. BD (1) C 15- H 16	167. BD*(1) C 13- C 14	2.3	9.5
69. BD (1) C 15- H 16	175. BD*(1) C 17- C 18	2.1	8.6
71. BD (2) C 15- C 17	168. BD*(2) C 13- C 14	9.5	39.6
71. BD (2) C 15- C 17	179. BD*(2) C 18- C 20	8.9	37.2
71. BD (2) C 15- C 17	209. BD*(1) C 51- C 53	1.7	7.1
74. BD (1) C 18- H 19	169. BD*(1) C 13- C 20	2.3	9.4
74. BD (1) C 18- H 19	173. BD*(1) C 15- C 17	2.1	8.9
76. BD (2) C 18- C 20	168. BD*(2) C 13- C 14	8.6	35.8
76. BD (2) C 18- C 20	174. BD*(2) C 15- C 17	11.4	47.6
78. BD (1) C 21- H 22	164. BD*(1) C 10- C 12	2.0	8.3
80. BD (1) C 21- C 27	165. BD*(2) C 10- C 12	1.6	6.6
82. BD (1) C 23- H 25	166. BD*(1) C 12- C 21	1.5	6.3
83. BD (1) C 23- H 26	183. BD*(1) C 21- C 27	1.5	6.3
87. BD (1) C 31- H 32	156. BD*(1) C 5- C 6	1.8	7.5
93. BD (1) C 37- H 38	157. BD*(1) C 5- C 31	1.5	6.4
96. BD (1) C 41- H 42	170. BD*(1) C 14- C 15	2.0	8.2
105. BD (1) C 51- H 52	173. BD*(1) C 15- C 17	1.9	7.9

106. BD (1) C 51- C 53	174. BD*(2) C 15- C 17	1.8	7.6
114. BD (1) C 61- H 62	178. BD*(1) C 18- C 20	2.0	8.4
119. BD (1) C 63- H 66	219. BD*(1) C 61- C 67	1.5	6.4
120. BD (1) C 67- H 68	180. BD*(1) C 20- C 61	1.5	6.3
124. BD (2) C 71- N 72	149. BD*(2) C 2- B 3	1.7	7.0
124. BD (2) C 71- N 72	236. BD*(1) C 77- C 86	2.2	9.3
124. BD (2) C 71- N 72	237. BD*(1) C 77- C 90	1.5	6.4
126. BD (1) N 72- C 77	152. BD*(1) B 3- C 71	2.4	9.8
135. BD (1) C 78- H 79	228. BD*(1) C 71- C 76	1.7	7.3
138. BD (1) C 82- H 83	228. BD*(1) C 71- C 76	1.6	6.7
141. BD (1) C 86- H 87	229. BD*(1) N 72- C 77	1.7	7.0
143. BD (1) C 86- H 89	233. BD*(1) C 73- C 77	1.6	6.5
144. BD (1) C 90- H 91	229. BD*(1) N 72- C 77	1.8	7.7
145. BD (1) C 90- H 92	233. BD*(1) C 73- C 77	1.5	6.3

Table S6. Second order perturbation theory analysis of [**3**]$^{\bullet-}$ (β system). Only terms above 1.5 kcal/mol are shown.

Donor NBO	Acceptor NBO	E(2)	
		kcal/mol	kJ/mol
40. LP (1) O 1	149. BD*(1) C 2- B 3	2.5	10.5
40. LP (1) O 1	264. RY (1) C 2	4.1	17.2
41. LP (2) O 1	149. BD*(1) C 2- B 3	3.5	14.7
41. LP (2) O 1	150. BD*(1) C 2- C 4	8.1	33.8
42. LP (1) N 72	228. BD*(1) C 71- C 76	4.6	19.3
42. LP (1) N 72	233. BD*(1) C 73- C 77	2.9	12.0
42. LP (1) N 72	853. RY (1) C 71	2.6	11.0
44. BD (2) O 1- C 2	146. LV (1) B 3	6.7	28.0
46. BD (1) C 2- C 4	156. BD*(1) C 5- C 6	1.5	6.2
46. BD (1) C 2- C 4	164. BD*(1) C 10- C 12	1.5	6.4
47. BD (1) B 3- C 13	150. BD*(1) C 2- C 4	1.7	7.1
47. BD (1) B 3- C 13	170. BD*(1) C 14- C 15	2.5	10.6

47. BD (1) B 3- C 13	178. BD*(1) C 18- C 20	2.3	9.8
48. BD (1) B 3- C 71	147. BD*(1) O 1- C 2	1.5	6.2
48. BD (1) B 3- C 71	229. BD*(1) N 72- C 77	2.6	10.7
50. BD (2) C 4- C 5	147. BD*(1) O 1- C 2	2.2	9.0
50. BD (2) C 4- C 5	160. BD*(2) C 6- C 8	11.8	49.5
50. BD (2) C 4- C 5	165. BD*(2) C 10- C 12	10.7	44.6
50. BD (2) C 4- C 5	191. BD*(1) C 31- C 33	1.9	8.1
52. BD (1) C 5- C 6	150. BD*(1) C 2- C 4	1.5	6.3
54. BD (1) C 6- H 7	153. BD*(1) C 4- C 5	2.2	9.2
54. BD (1) C 6- H 7	162. BD*(1) C 8- C 10	1.7	7.2
56. BD (2) C 6- C 8	154. BD*(2) C 4- C 5	8.8	36.9
56. BD (2) C 6- C 8	165. BD*(2) C 10- C 12	8.8	36.9
57. BD (1) C 8- H 9	156. BD*(1) C 5- C 6	1.7	7.1
57. BD (1) C 8- H 9	164. BD*(1) C 10- C 12	1.7	7.0
59. BD (1) C 10- H 11	155. BD*(1) C 4- C 12	2.2	9.4
59. BD (1) C 10- H 11	159. BD*(1) C 6- C 8	1.7	7.1
60. BD (1) C 10- C 12	150. BD*(1) C 2- C 4	1.5	6.1
61. BD (2) C 10- C 12	154. BD*(2) C 4- C 5	9.3	39.0
61. BD (2) C 10- C 12	160. BD*(2) C 6- C 8	11.3	47.4
63. BD (1) C 13- C 14	151. BD*(1) B 3- C 13	1.6	6.7
63. BD (1) C 13- C 14	180. BD*(1) C 20- C 61	1.5	6.4
64. BD (2) C 13- C 14	149. BD*(1) C 2- B 3	1.6	6.6
64. BD (2) C 13- C 14	152. BD*(1) B 3- C 71	1.6	6.6
64. BD (2) C 13- C 14	174. BD*(2) C 15- C 17	11.2	46.7
64. BD (2) C 13- C 14	179. BD*(2) C 18- C 20	11.5	48.1
64. BD (2) C 13- C 14	201. BD*(1) C 41- C 47	1.7	7.3
65. BD (1) C 13- C 20	151. BD*(1) B 3- C 13	1.6	6.8
65. BD (1) C 13- C 20	171. BD*(1) C 14- C 41	1.5	6.2
66. BD (1) C 14- C 15	176. BD*(1) C 17- C 51	1.5	6.1
68. BD (1) C 15- H 16	167. BD*(1) C 13- C 14	2.3	9.6
68. BD (1) C 15- H 16	175. BD*(1) C 17- C 18	2.1	8.6
70. BD (2) C 15- C 17	168. BD*(2) C 13- C 14	9.6	40.3

70. BD (2) C 15- C 17	179. BD*(2) C 18- C 20	8.7	36.4
70. BD (2) C 15- C 17	209. BD*(1) C 51- C 53	1.7	7.0
73. BD (1) C 18- H 19	169. BD*(1) C 13- C 20	2.3	9.5
73. BD (1) C 18- H 19	173. BD*(1) C 15- C 17	2.1	8.9
75. BD (2) C 18- C 20	168. BD*(2) C 13- C 14	8.4	35.1
75. BD (2) C 18- C 20	174. BD*(2) C 15- C 17	11.6	48.5
77. BD (1) C 21- H 22	164. BD*(1) C 10- C 12	2.0	8.3
79. BD (1) C 21- C 27	165. BD*(2) C 10- C 12	1.6	6.6
81. BD (1) C 23- H 25	166. BD*(1) C 12- C 21	1.5	6.3
82. BD (1) C 23- H 26	183. BD*(1) C 21- C 27	1.5	6.3
84. BD (1) C 27- H 29	182. BD*(1) C 21- C 23	1.5	6.1
86. BD (1) C 31- H 32	156. BD*(1) C 5- C 6	1.8	7.5
87. BD (1) C 31- C 33	154. BD*(2) C 4- C 5	1.5	6.1
90. BD (1) C 33- H 35	192. BD*(1) C 31- C 37	1.5	6.2
92. BD (1) C 37- H 38	157. BD*(1) C 5- C 31	1.5	6.4
95. BD (1) C 41- H 42	146. LV (1) B 3	2.2	9.2
95. BD (1) C 41- H 42	170. BD*(1) C 14- C 15	2.0	8.2
98. BD (1) C 43- H 44	171. BD*(1) C 14- C 41	1.5	6.2
104. BD (1) C 51- H 52	173. BD*(1) C 15- C 17	1.9	7.9
105. BD (1) C 51- C 53	174. BD*(2) C 15- C 17	1.8	7.7
111. BD (1) C 57- H 59	176. BD*(1) C 17- C 51	1.5	6.1
113. BD (1) C 61- H 62	146. LV (1) B 3	1.9	7.9
113. BD (1) C 61- H 62	178. BD*(1) C 18- C 20	2.0	8.4
116. BD (1) C 63- H 64	180. BD*(1) C 20- C 61	1.5	6.1
118. BD (1) C 63- H 66	219. BD*(1) C 61- C 67	1.5	6.4
119. BD (1) C 67- H 68	180. BD*(1) C 20- C 61	1.5	6.3
123. BD (2) C 71- N 72	146. LV (1) B 3	3.9	16.4
123. BD (2) C 71- N 72	235. BD*(1) C 76- C 82	1.6	6.6
123. BD (2) C 71- N 72	236. BD*(1) C 77- C 86	2.1	8.7
125. BD (1) N 72- C 77	152. BD*(1) B 3- C 71	2.3	9.5
125. BD (1) N 72- C 77	855. RY (3) C 71	1.5	6.1
134. BD (1) C 78- H 79	228. BD*(1) C 71- C 76	1.8	7.3

135. BD (1) C 78- H 80	235. BD*(1) C 76- C 82	1.5	6.2

Table S7. MBOs of **4-H** and **4-K** at B3LYP-D3(BJ)/def2-SVP.

	**4-H**	**4-K**
N1–C1	1.196	1.529
C1–B1	1.472	1.270
B1–C36	1.353	1.564
C36–O1	1.363	1.215

Cartesian coordinates

**2** (singlet)			
C	-0.712716000	0.049628000	0.940316000
C	-2.695195000	-0.148819000	2.311637000
C	-1.529146000	0.324574000	3.192401000
H	-1.519428000	-0.177403000	4.170520000
H	-1.638864000	1.403743000	3.379383000
C	-0.226314000	0.083132000	2.394372000
C	-3.919093000	0.758851000	2.446757000
H	-4.697494000	0.494467000	1.715692000
H	-3.656488000	1.814617000	2.313000000
H	-4.347080000	0.642943000	3.453925000
C	-3.126207000	-1.582825000	2.653479000
H	-3.940318000	-1.914028000	1.997148000
H	-3.497908000	-1.613269000	3.688867000
H	-2.298187000	-2.297602000	2.569881000
C	0.441201000	-1.258876000	2.753275000
H	0.670638000	-1.290602000	3.830775000
H	1.381574000	-1.372385000	2.199382000
H	-0.198035000	-2.120221000	2.512790000
C	0.766628000	1.219223000	2.681636000
H	0.950912000	1.277457000	3.767120000
H	0.364543000	2.189039000	2.352946000
H	1.727405000	1.058067000	2.179133000
C	-2.838510000	-0.073426000	-0.258581000
C	-3.119537000	-1.300049000	-0.911403000
C	-3.882948000	-1.269573000	-2.085707000
H	-4.110103000	-2.206603000	-2.597879000
C	-4.335768000	-0.069667000	-2.624447000
H	-4.925750000	-0.069231000	-3.544044000
C	-4.011139000	1.132162000	-2.001941000
H	-4.340290000	2.073031000	-2.447393000

C	-3.255350000	1.159709000	-0.824074000
C	-2.569441000	-2.641701000	-0.447831000
H	-1.979000000	-2.463943000	0.458533000
C	-3.683025000	-3.650279000	-0.123491000
H	-3.256539000	-4.568855000	0.310476000
H	-4.229463000	-3.939942000	-1.035046000
H	-4.419879000	-3.248244000	0.585968000
C	-1.619100000	-3.241214000	-1.497131000
H	-1.133845000	-4.146882000	-1.099740000
H	-0.834746000	-2.531242000	-1.779918000
H	-2.161523000	-3.527260000	-2.412133000
C	-2.865942000	2.514594000	-0.247597000
H	-2.300475000	2.334199000	0.676268000
C	-1.936825000	3.273475000	-1.209282000
H	-1.568870000	4.199018000	-0.738078000
H	-2.464568000	3.555297000	-2.134306000
H	-1.069499000	2.665071000	-1.487936000
C	-4.090713000	3.380048000	0.089924000
H	-3.777432000	4.306749000	0.597056000
H	-4.803297000	2.855967000	0.741912000
H	-4.632122000	3.673934000	-0.823140000
C	1.788411000	0.141168000	-0.266580000
C	2.480436000	1.378444000	-0.275964000
C	3.879610000	1.401197000	-0.206599000
H	4.404580000	2.359590000	-0.205251000
C	4.635573000	0.228392000	-0.141389000
C	3.951681000	-0.990799000	-0.174209000
H	4.524815000	-1.920114000	-0.151928000
C	2.554768000	-1.053364000	-0.250226000
C	1.723643000	2.692803000	-0.414552000
H	0.693296000	2.498347000	-0.081928000
C	1.656193000	3.102848000	-1.895014000
H	1.072324000	4.028437000	-2.025593000
H	1.190542000	2.315758000	-2.506494000

H	2.668740000	3.278191000	-2.294137000
C	2.281535000	3.835508000	0.441461000
H	1.606919000	4.706104000	0.400372000
H	3.267490000	4.175997000	0.086528000
H	2.390073000	3.536646000	1.495172000
C	6.151473000	0.280215000	-0.050163000
H	6.437243000	1.346346000	-0.054307000
C	6.659129000	-0.330049000	1.264921000
H	7.754291000	-0.234826000	1.347386000
H	6.410961000	-1.402218000	1.325929000
H	6.203558000	0.167477000	2.135083000
C	6.818935000	-0.382040000	-1.264693000
H	7.915480000	-0.280518000	-1.215372000
H	6.472440000	0.073274000	-2.205234000
H	6.585500000	-1.458233000	-1.309137000
C	1.888684000	-2.414998000	-0.407842000
H	0.833981000	-2.289700000	-0.120472000
C	2.485721000	-3.519827000	0.471598000
H	1.876980000	-4.435592000	0.397991000
H	2.524930000	-3.221848000	1.530424000
H	3.508466000	-3.788204000	0.161981000
C	1.917658000	-2.827665000	-1.889285000
H	1.403633000	-3.789413000	-2.046825000
H	2.957610000	-2.934313000	-2.238901000
H	1.432057000	-2.070793000	-2.523269000
C	-0.388637000	0.069242000	-1.621403000
B	0.180585000	0.089498000	-0.263911000
N	-2.073463000	-0.055622000	0.951748000
O	-0.684063000	0.075664000	-2.745014000

[**2**]$^{\bullet-}$ (doublet)

C	-0.655862050	0.014917001	0.901365066
C	-2.576694187	-0.611836042	2.216087160
C	-1.600127113	0.173777013	3.119160225

H	-1.503935109	-0.268268019	4.117924295
H	-1.989966145	1.191565086	3.243072232
C	-0.246463018	0.234228017	2.362102171
C	-4.028442291	-0.171441013	2.417199175
H	-4.691929340	-0.661969046	1.697733121
H	-4.133674298	0.907978067	2.291400164
H	-4.360425316	-0.437577031	3.427375249
C	-2.479198179	-2.129395153	2.510221180
H	-3.266022237	-2.684710191	1.995679142
H	-2.586325187	-2.316163167	3.586258257
H	-1.513948111	-2.525028183	2.188207155
C	0.738380055	-0.841270062	2.880482209
H	0.956140071	-0.686388052	3.947506287
H	1.679210123	-0.784194057	2.328501167
H	0.341089024	-1.851001135	2.756483200
C	0.423468031	1.604496117	2.606861185
H	0.586398042	1.775865126	3.682110265
H	-0.195079014	2.419955172	2.219643157
H	1.393493102	1.648318121	2.107623150
C	-2.873522205	0.015691001	-0.247264018
C	-3.373747241	-1.052688073	-1.035778077
C	-4.235344303	-0.776538058	-2.102824152
H	-4.615514330	-1.597536113	-2.705669193
C	-4.572288328	0.532167037	-2.439705173
H	-5.232649379	0.732347054	-3.279443235
C	-4.019901290	1.585205113	-1.709874124
H	-4.256333304	2.608577185	-1.990141144
C	-3.176031227	1.354754100	-0.621339044
C	-2.903949209	-2.481287180	-0.820006059
H	-2.207686159	-2.459541176	0.018243001
C	-4.061985291	-3.436054248	-0.483445035
H	-3.682675267	-4.436298321	-0.239479017
H	-4.745786344	-3.537658253	-1.334818098
H	-4.649250332	-3.075137224	0.367252027

C	-2.117985155	-2.992750216	-2.041452149
H	-1.691014120	-3.981436285	-1.830912133
H	-1.305848094	-2.306915165	-2.288773164
H	-2.765333197	-3.085159222	-2.921827208
C	-2.605084187	2.546586182	0.129609009
H	-1.952310140	2.150453153	0.907452065
C	-1.742993127	3.419716244	-0.798538057
H	-1.218496086	4.193004302	-0.223609016
H	-2.358939167	3.922863280	-1.554091110
H	-1.002906073	2.804912200	-1.312845092
C	-3.699064268	3.397766247	0.798793057
H	-3.246893236	4.209222304	1.382806098
H	-4.323019310	2.801200202	1.471004107
H	-4.359770316	3.852660277	0.051203004
C	1.822777133	0.126030009	-0.283906020
C	2.580459188	1.319919097	-0.344188025
C	3.981874285	1.276551090	-0.301296021
H	4.551998328	2.202356159	-0.344896025
C	4.677546339	0.070835005	-0.208372015
C	3.932711285	-1.112132078	-0.167955012
H	4.455097321	-2.064039148	-0.111338008
C	2.533604183	-1.100428079	-0.205470015
C	1.877371133	2.660237193	-0.509390037
H	0.852612061	2.506921183	-0.161152011
C	1.799335131	3.047256219	-1.997294144
H	1.257717089	3.993156286	-2.125624151
H	1.278502092	2.275388165	-2.569729183
H	2.806108204	3.169035228	-2.417248177
C	2.496366181	3.801701275	0.311408022
H	1.860348136	4.693124338	0.250227018
H	3.489302253	4.084757296	-0.059298004
H	2.596721186	3.527542252	1.366614099
C	6.196493444	0.051808004	-0.158280011
H	6.533862462	1.096123079	-0.212411015

C	6.717138504	-0.536149038	1.165451083
H	7.812783541	-0.491885036	1.212897089
H	6.416756436	-1.584833112	1.269643093
H	6.309704461	0.011341001	2.021435146
C	6.801733467	-0.693996049	-1.360885100
H	7.898123585	-0.648212046	-1.341474095
H	6.452225441	-0.261019019	-2.303614168
H	6.508575462	-1.749806125	-1.352351095
C	1.776001129	-2.423018172	-0.229265017
H	0.751947054	-2.192912160	0.082091006
C	2.341841170	-3.486545250	0.723601050
H	1.678000122	-4.359522312	0.747575055
H	2.432330173	-3.099297224	1.743361127
H	3.331137238	-3.840733278	0.407828029
C	1.705392125	-2.966317214	-1.667915121
H	1.111078078	-3.887815278	-1.708858125
H	2.710862194	-3.188340228	-2.048536149
H	1.241146089	-2.232370161	-2.331876170
C	-0.300042022	0.144544010	-1.701975122
B	0.208586015	0.119985009	-0.328931024
N	-2.061740148	-0.243224018	0.882684061
O	-0.606362042	0.152711011	-2.861100203

3 (closed-shell singlet)

O	-1.578546000	0.177720000	-0.036090000
C	-2.808387000	0.565621000	0.029409000
B	-3.996898000	-0.386673000	-0.176035000
K	-0.245515000	-2.031175000	0.180091000
C	-2.945155000	2.038938000	0.358211000
C	-3.098002000	3.005788000	-0.663199000
C	-3.009863000	4.366977000	-0.332419000
H	-3.112624000	5.122452000	-1.113776000
C	-2.786658000	4.774015000	0.983413000
H	-2.712550000	5.837762000	1.222688000

C	-2.680928000	3.816756000	1.994516000
H	-2.542195000	4.140302000	3.029077000
C	-2.771049000	2.449318000	1.701519000
C	-3.650732000	-1.913247000	-0.458342000
C	-3.682614000	-2.841035000	0.615141000
C	-3.199437000	-4.149477000	0.442466000
H	-3.206978000	-4.832969000	1.294153000
C	-2.677943000	-4.586401000	-0.782838000
C	-2.685882000	-3.679458000	-1.852432000
H	-2.288672000	-4.007848000	-2.818267000
C	-3.149620000	-2.362754000	-1.708136000
C	-2.697493000	1.417119000	2.817306000
H	-3.021325000	0.465721000	2.376443000
C	-3.680190000	1.727783000	3.951744000
H	-3.416804000	2.651869000	4.492001000
H	-3.686479000	0.907103000	4.687719000
H	-4.693971000	1.832936000	3.540708000
C	-1.263891000	1.226757000	3.334958000
H	-0.836502000	2.173847000	3.705664000
H	-0.605194000	0.832889000	2.545101000
H	-1.235852000	0.502291000	4.163793000
C	-3.287406000	2.573974000	-2.111424000
H	-3.761663000	1.581600000	-2.079485000
C	-1.927569000	2.406353000	-2.809031000
H	-2.050621000	2.097725000	-3.859314000
H	-1.323874000	1.634488000	-2.312900000
H	-1.364192000	3.355303000	-2.801765000
C	-4.208690000	3.497916000	-2.913809000
H	-5.155443000	3.678433000	-2.382652000
C	-4.126706000	-2.397280000	2.006138000
H	-4.571206000	-1.395786000	1.904175000
C	-5.195697000	-3.311296000	2.617368000
H	-5.543587000	-2.908648000	3.582238000
H	-4.811510000	-4.327613000	2.803064000

H	-6.067023000	-3.400183000	1.950620000
C	-2.911356000	-2.256153000	2.937905000
H	-2.173277000	-1.558178000	2.514574000
H	-2.414296000	-3.227186000	3.100141000
H	-3.206400000	-1.859749000	3.922555000
C	-2.007055000	-5.942680000	-0.946999000
H	-2.109620000	-6.225214000	-2.009483000
C	-0.500605000	-5.816637000	-0.649935000
H	-0.022076000	-5.065864000	-1.298785000
H	0.024479000	-6.772931000	-0.806488000
H	-0.330371000	-5.513117000	0.396817000
C	-2.638566000	-7.056770000	-0.106461000
H	-2.481073000	-6.890275000	0.971394000
H	-2.186150000	-8.030089000	-0.353238000
H	-3.723014000	-7.126824000	-0.281586000
C	-3.048718000	-1.415620000	-2.897919000
H	-3.476020000	-0.454996000	-2.570166000
C	-1.584791000	-1.150120000	-3.280193000
H	-1.514428000	-0.434879000	-4.114550000
H	-1.075178000	-2.076148000	-3.593486000
H	-1.037880000	-0.721284000	-2.428483000
C	-3.864896000	-1.904278000	-4.102183000
H	-3.851068000	-1.157188000	-4.912356000
H	-4.914175000	-2.087491000	-3.824905000
H	-3.458904000	-2.844387000	-4.509971000
C	-5.450196000	0.174886000	0.029147000
N	-5.673562000	1.037617000	0.963425000
C	-7.811938000	0.547999000	-0.017985000
H	-8.391389000	-0.189834000	0.558566000
H	-8.522557000	1.077443000	-0.670809000
C	-7.068921000	1.505035000	0.953314000
C	-6.936166000	-1.683027000	-0.910894000
H	-7.893941000	-1.886839000	-1.418295000
H	-6.137268000	-2.187824000	-1.472349000

C	-6.535713000	0.410749000	-2.234031000
H	-7.430862000	0.199610000	-2.841981000
H	-5.669518000	-0.030942000	-2.748146000
H	-6.390722000	1.500309000	-2.210694000
C	-7.057678000	2.961944000	0.461204000
H	-8.069577000	3.397336000	0.495486000
H	-6.688644000	3.023549000	-0.573180000
H	-6.386871000	3.567810000	1.088856000
C	-7.631356000	1.442011000	2.376566000
H	-8.690805000	1.745302000	2.393823000
H	-7.068810000	2.109747000	3.047100000
O	1.553534000	-0.138336000	0.012488000
C	2.772956000	-0.560978000	0.082821000
B	3.980231000	0.365121000	-0.133017000
K	0.229994000	2.078205000	0.130584000
C	2.879235000	-2.031915000	0.423957000
C	3.133170000	-2.998658000	-0.579678000
C	3.073235000	-4.359678000	-0.247901000
H	3.256045000	-5.113368000	-1.015808000
C	2.773860000	-4.771562000	1.051290000
H	2.730451000	-5.836640000	1.292086000
C	2.538485000	-3.817062000	2.041102000
H	2.329075000	-4.142921000	3.062788000
C	2.585383000	-2.446896000	1.746418000
C	3.648214000	1.900052000	-0.391409000
C	3.641255000	2.798190000	0.706443000
C	3.192929000	4.120417000	0.543619000
H	3.175770000	4.784179000	1.410998000
C	2.736290000	4.595798000	-0.693306000
C	2.770983000	3.712568000	-1.782427000
H	2.417902000	4.069407000	-2.755283000
C	3.206640000	2.384961000	-1.649549000
C	2.403276000	-1.427543000	2.864037000
H	2.288419000	-0.445221000	2.390702000

C	3.670136000	-1.368735000	3.731474000
H	3.820250000	-2.317022000	4.274361000
H	3.595580000	-0.560535000	4.477723000
H	4.547007000	-1.196745000	3.090826000
C	1.148071000	-1.672023000	3.710468000
H	1.193265000	-2.629815000	4.252952000
H	0.229579000	-1.678646000	3.101176000
H	1.029612000	-0.875247000	4.461374000
C	3.378902000	-2.564980000	-2.018983000
H	3.871560000	-1.582827000	-1.969615000
C	2.040510000	-2.364780000	-2.750380000
H	2.197662000	-2.042835000	-3.792137000
H	1.433795000	-1.592446000	-2.258521000
H	1.463280000	-3.305022000	-2.771964000
C	4.298100000	-3.503105000	-2.805664000
H	4.568443000	-3.048145000	-3.771395000
H	3.814462000	-4.468809000	-3.025771000
H	5.227030000	-3.710620000	-2.253774000
C	4.023853000	2.315915000	2.103177000
H	4.360608000	1.272152000	2.006321000
C	5.183556000	3.117484000	2.708030000
H	5.476760000	2.700788000	3.685074000
H	4.911375000	4.174346000	2.864056000
H	6.064702000	3.093605000	2.049169000
C	2.803669000	2.309544000	3.038587000
H	1.993735000	1.684892000	2.630375000
H	2.406071000	3.326359000	3.192413000
H	3.064798000	1.898921000	4.026643000
C	2.100949000	5.969642000	-0.853467000
H	2.241524000	6.267730000	-1.907260000
C	0.583823000	5.873298000	-0.603282000
H	0.108920000	5.144075000	-1.278950000
H	0.085205000	6.843818000	-0.759031000
H	0.376027000	5.556364000	0.432731000

C	2.732416000	7.054373000	0.024838000
H	2.540284000	6.872770000	1.094589000
H	2.309353000	8.042026000	-0.217108000
H	3.822757000	7.102388000	-0.118080000
C	3.126139000	1.459125000	-2.857863000
H	3.554832000	0.494644000	-2.542688000
C	1.666468000	1.191181000	-3.255456000
H	1.605954000	0.490986000	-4.103349000
H	1.154825000	2.120662000	-3.554922000
H	1.115968000	0.743252000	-2.415946000
C	3.946762000	1.971740000	-4.048695000
H	3.947210000	1.234675000	-4.868016000
H	4.991849000	2.162728000	-3.761414000
H	3.533807000	2.911907000	-4.449130000
C	5.437618000	-0.202773000	0.032622000
N	5.671692000	-1.101965000	0.929269000
C	7.807322000	-0.529558000	-0.005080000
H	8.355482000	0.204603000	0.605823000
H	8.543268000	-1.015677000	-0.663508000
C	6.682939000	0.184473000	-0.797484000
C	7.076372000	-1.537763000	0.921335000
C	6.904986000	1.701242000	-0.865629000
H	7.867439000	1.924181000	-1.355789000
H	6.926114000	2.144800000	0.141596000
H	6.108595000	2.205029000	-1.430802000
C	6.550130000	-0.375177000	-2.226378000
H	7.447482000	-0.134099000	-2.819663000
H	5.681478000	0.056923000	-2.743489000
H	6.429019000	-1.467612000	-2.224149000
C	7.111522000	-2.978096000	0.384463000
H	8.133255000	-3.388884000	0.426981000
H	6.766836000	-3.017267000	-0.659072000
H	6.444079000	-3.619087000	0.979893000
C	7.614334000	-1.508127000	2.355693000

H	8.678444000	-1.794203000	2.381517000
H	7.050395000	-2.203950000	2.995568000
H	7.516698000	-0.497424000	2.782083000
H	-4.444838000	3.046203000	-3.890080000
H	-3.742803000	4.476853000	-3.112393000
C	-6.694572000	-0.170162000	-0.816818000
H	-6.977640000	-2.140641000	0.089763000
H	-7.557258000	0.417618000	2.774134000

3 (open-shell singlet)

O	-1.630268065	-0.186501990	0.065506005
C	-2.863477156	-0.569354000	-0.025156002
B	-4.044870233	0.388481082	0.136463010
K	-0.260868938	2.021896150	-0.116723008
C	-3.002337188	-2.051107104	-0.322499023
C	-3.104050204	-2.991383171	0.729828054
C	-3.039865221	-4.361555273	0.430707031
H	-3.106579232	-5.095933325	1.235801087
C	-2.888468213	-4.802911308	-0.883532062
H	-2.834079222	-5.872782386	-1.099771079
C	-2.825391195	-3.871883238	-1.922134136
H	-2.734897197	-4.223068264	-2.952710215
C	-2.892278184	-2.496343141	-1.661803118
C	-3.697567188	1.920895189	0.400631029
C	-3.674695174	2.822525251	-0.693981052
C	-3.189199120	4.131437339	-0.530283039
H	-3.155053111	4.795878389	-1.396409102
C	-2.717996080	4.592282369	0.706776050
C	-2.780199099	3.710465307	1.795571131
H	-2.422022070	4.057851327	2.769946199
C	-3.249737151	2.394424217	1.660756120
C	-2.823703168	-1.493657068	-2.804789203
H	-3.173983179	-0.537527994	-2.396458173
C	-3.767872238	-1.848083082	-3.957754286

H	-3.471335230	-2.775867153	-4.473788322
H	-3.770466228	-1.042026025	-4.709598341
H	-4.790590316	-1.964736079	-3.573847257
C	-1.379866063	-1.294604072	-3.289877235
H	-0.933186039	-2.242912145	-3.633486263
H	-0.750263011	-0.884565053	-2.486048178
H	-1.334434048	-0.580123021	-4.126889299
C	-3.221290207	-2.527769136	2.176704154
H	-3.662498226	-1.520970060	2.148189153
C	-1.831851105	-2.398848144	2.822624202
H	-1.905921108	-2.085869123	3.876135278
H	-1.228002056	-1.642575098	2.303060167
H	-1.295150082	-3.362559222	2.795890199
C	-4.143964288	-3.407134187	3.027544218
H	-5.115287358	-3.562200187	2.533972184
C	-4.067398208	2.349272226	-2.090545150
H	-4.522098252	1.353167155	-1.984502141
C	-5.110814271	3.252892301	-2.758838197
H	-5.423218319	2.832333276	-3.728197269
H	-4.718941230	4.265559371	-2.948524211
H	-6.006393344	3.353778319	-2.126455155
C	-2.818621119	2.182305194	-2.972609216
H	-2.102203078	1.486989139	-2.509245181
H	-2.309377072	3.147307258	-3.134253228
H	-3.080061144	1.770155171	-3.960299284
C	-2.044071015	5.947560476	0.867208061
H	-2.168437025	6.245210454	1.923141139
C	-0.532098911	5.811168427	0.605128043
H	-0.071579886	5.067046370	1.274358089
H	-0.005587860	6.766771467	0.760901056
H	-0.340195900	5.493701395	-0.433828031
C	-2.652625047	7.052517550	-0.002119000
H	-2.474531037	6.869515516	-1.074049075
H	-2.200466001	8.027273632	0.239560017

H	-3.739996123	7.129968568	0.150146011
C	-3.205066156	1.470781149	2.872398206
H	-3.644091202	0.513549086	2.553178185
C	-1.758574058	1.178836108	3.299567238
H	-1.728297065	0.478235059	4.148848297
H	-1.236408005	2.098870172	3.609134261
H	-1.200255023	0.720971071	2.470527176
C	-4.042606212	1.998868197	4.044639290
H	-4.063052224	1.268229148	4.869509350
H	-5.081126284	2.193782223	3.736571266
H	-3.630803170	2.939656259	4.444720320
C	-5.505962326	-0.158894939	-0.094371007
N	-5.731507354	-0.974087995	-1.066223078
C	-7.867688538	-0.530228936	-0.055967004
H	-8.456146551	0.230385127	-0.592239042
H	-8.569707565	-1.093118967	0.577800039
C	-7.129253490	-1.438780012	-1.076246076
C	-6.982130415	1.665525213	0.911663067
H	-7.939254469	1.856752239	1.425134102
H	-6.181393372	2.148123238	1.489399107
C	-6.586052409	-0.476920948	2.160942155
H	-7.483205494	-0.294822924	2.775176201
H	-5.723633355	-0.049947928	2.693604195
H	-6.433479443	-1.563870031	2.095559152
C	-7.118486480	-2.917444118	-0.655291046
H	-8.133015576	-3.345956138	-0.696504048
H	-6.734465461	-3.030710132	0.369516027
H	-6.460309470	-3.496532169	-1.320444094
C	-7.696460539	-1.303078994	-2.492199180
H	-8.756402621	-1.603728000	-2.520557181
H	-7.138301469	-1.936638048	-3.198500230
O	1.630356173	0.186744996	0.066182005
C	2.863567270	0.569526009	-0.024730002
B	4.044935343	-0.388400076	0.136578010

K	0.260875049	-2.021845147	-0.116430009
C	3.002395297	2.051317113	-0.321970023
C	3.103876316	2.991520180	0.730446053
C	3.039614328	4.361711280	0.431424031
H	3.106147344	5.096028333	1.236588090
C	2.888355324	4.803164313	-0.882796061
H	2.833909335	5.873047398	-1.098956078
C	2.825494306	3.872214244	-1.921481136
H	2.735109308	4.223479271	-2.952037213
C	2.892467296	2.496659145	-1.661255118
C	3.697582296	-1.920798180	0.400791029
C	3.674672282	-2.822521245	-0.693756051
C	3.189092232	-4.131386332	-0.529969036
H	3.154891220	-4.795879384	-1.396053103
C	2.717856192	-4.592123362	0.707119052
C	2.780126210	-3.710240301	1.795851129
H	2.421918176	-4.057532318	2.770248201
C	3.249736257	-2.394232210	1.660946121
C	2.824065278	1.494048077	-2.804327200
H	3.175002289	0.538080000	-2.396175174
C	3.767632351	1.849050090	-3.957597284
H	3.470349341	2.776630158	-4.473567322
H	3.770488337	1.042977030	-4.709420341
H	4.790415423	1.966362082	-3.574057255
C	1.380162169	1.294291080	-3.288949238
H	0.932800152	2.242452152	-3.632088261
H	0.751127120	0.883593055	-2.485015181
H	1.334832158	0.580072030	-4.126188298
C	3.220934319	2.527825144	2.177309157
H	3.662165336	1.521037064	2.148804157
C	1.831403214	2.398887155	2.823017201
H	1.905304220	2.085828127	3.876515280
H	1.227609163	1.642693104	2.303284167
H	1.294739192	3.362620231	2.796278202

C	4.143488398	3.407165197	3.028313218
H	4.327988401	2.933208160	4.005298290
H	3.704549375	4.398635273	3.225197233
H	5.114892470	3.562188196	2.534891185
C	4.067254320	-2.349359218	-2.090388148
H	4.522346362	-1.353428151	-1.984412144
C	5.110132383	-3.253328296	-2.759043201
H	5.422525415	-2.832727270	-3.728387270
H	4.717777341	-4.265782361	-2.948860212
H	6.005800454	-3.354737316	-2.126867151
C	2.818288229	-2.181896188	-2.972095216
H	2.102252186	-1.486368128	-2.508480181
H	2.308685183	-3.146726253	-3.133646225
H	3.079581254	-1.769781164	-3.959839286
C	2.043830128	-5.947343460	0.867604060
H	2.167883135	-6.244808491	1.923626140
C	0.531936019	-5.810950406	0.605073046
H	0.071244996	-5.066728361	1.274076091
H	0.005353970	-6.766515504	0.760843053
H	0.340325011	-5.493633396	-0.433982031
C	2.652608156	-7.052473523	-0.001351000
H	2.474874145	-6.869632517	-1.073369077
H	2.200325113	-8.027169596	0.240336017
H	3.739926234	-7.129947559	0.151280011
C	3.205200265	-1.470579140	2.872584207
H	3.644106310	-0.513322079	2.553282184
C	1.758776167	-1.178742105	3.300037237
H	1.728624171	-0.478150053	4.149328298
H	1.236711118	-2.098803162	3.609701259
H	1.200259135	-0.720902066	2.471115177
C	4.043017319	-1.998630189	4.044650291
H	4.063555334	-1.268020139	4.869545350
H	5.081492395	-2.193431220	3.736369269
H	3.631377280	-2.939476254	4.444761320

C	5.506047471	0.158798945	-0.094554007
N	5.731543496	0.974072004	-1.066351075
C	7.867904654	0.529375944	-0.056819004
H	8.455937684	-0.231375120	-0.593363041
H	8.570308710	1.091955973	0.576794041
C	6.746952562	-0.155935093	0.765915053
C	7.129446587	1.438290015	-1.076767078
C	6.981980552	-1.666147203	0.910958065
H	7.939356600	-1.857669229	1.423842100
H	7.018652535	-2.157301241	-0.074069005
H	6.181454492	-2.148499229	1.489201105
C	6.586904543	0.476347954	2.160435156
H	7.483921577	0.293370930	2.774606198
H	5.724117446	0.050103935	2.693088194
H	6.435370564	1.563443032	2.095154151
C	7.119368618	2.916922122	-0.655697047
H	8.134061707	3.345027141	-0.697113051
H	6.735625576	3.030268137	0.369206027
H	6.461287548	3.496332172	-1.320664094
C	7.696142609	1.302483998	-2.492918179
H	8.756172694	1.602790007	-2.521610180
H	7.137953588	1.936264052	-3.198999229
H	7.621893590	0.259475926	-2.838783206
H	-4.328654291	-2.933182151	4.004495290
H	-3.705016268	-4.398621266	3.224531232
C	-6.746707447	0.155371099	0.766489056
H	-7.019580447	2.156630249	-0.073358005
H	-7.622667512	-0.260018920	-2.838035203

**3** (triplet)			
O	1.630967000	0.186390000	0.062863000
C	2.864298000	0.569042000	-0.026777000
B	4.045270000	-0.388975000	0.137016000
K	0.260303000	-2.021808000	-0.119563000

C	3.003553000	2.050491000	-0.325381000
C	3.105622000	2.991743000	0.726062000
C	3.041649000	4.361625000	0.425652000
H	3.108622000	5.096750000	1.230038000
C	2.890103000	4.801810000	-0.888982000
H	2.835884000	5.871495000	-1.106188000
C	2.826715000	3.869831000	-1.926692000
H	2.736215000	4.220027000	-2.957620000
C	2.893406000	2.494524000	-1.665050000
C	3.696873000	-1.921064000	0.401708000
C	3.674732000	-2.823310000	-0.692435000
C	3.188654000	-4.131969000	-0.528488000
H	3.155143000	-4.796894000	-1.394276000
C	2.716018000	-4.591955000	0.708354000
C	2.777261000	-3.709483000	1.796665000
H	2.417850000	-4.056151000	2.770843000
C	3.247396000	-2.393672000	1.661582000
C	2.825246000	1.490922000	-2.807249000
H	3.172178000	0.534297000	-2.397252000
C	3.773360000	1.842268000	-3.957952000
H	3.480650000	2.770690000	-4.475032000
H	3.775744000	1.035915000	-4.709494000
H	4.795329000	1.956313000	-3.571295000
C	1.382275000	1.294793000	-3.296020000
H	0.938929000	2.243684000	-3.642293000
H	0.749239000	0.887623000	-2.493410000
H	1.337365000	0.579120000	-4.132057000
C	3.222971000	2.529383000	2.173321000
H	3.665148000	1.522983000	2.145694000
C	1.833477000	2.399653000	2.818923000
H	1.907495000	2.087281000	3.872616000
H	1.230412000	1.642659000	2.299518000
H	1.296000000	3.362912000	2.791518000
C	4.144582000	3.410246000	3.023768000

H	5.115911000	3.565771000	2.530358000
C	4.068750000	-2.350938000	-2.088937000
H	4.523248000	-1.354710000	-1.983106000
C	5.112857000	-3.254927000	-2.755652000
H	5.426126000	-2.835019000	-3.725014000
H	4.721226000	-4.267755000	-2.944990000
H	6.007859000	-3.355316000	-2.122374000
C	2.820809000	-2.184709000	-2.972348000
H	2.103775000	-1.489253000	-2.510117000
H	2.311928000	-3.149915000	-3.133950000
H	3.083177000	-1.773088000	-3.960012000
C	2.041547000	-5.946955000	0.868829000
H	2.164148000	-6.243748000	1.925209000
C	0.530021000	-5.810499000	0.604214000
H	0.068561000	-5.065771000	1.272123000
H	0.003075000	-6.765875000	0.759921000
H	0.339833000	-5.493849000	-0.435310000
C	2.651308000	-7.052751000	0.001412000
H	2.475078000	-6.870561000	-1.070966000
H	2.198521000	-8.027214000	0.243101000
H	3.738405000	-7.130321000	0.155579000
C	3.201568000	-1.469320000	2.872638000
H	3.640757000	-0.512212000	2.553264000
C	1.754694000	-1.177374000	3.298448000
H	1.723587000	-0.476182000	4.147208000
H	1.232415000	-2.097265000	3.608223000
H	1.196912000	-0.720183000	2.468671000
C	4.038161000	-1.996725000	4.045879000
H	4.057865000	-1.265661000	4.870392000
H	5.076946000	-2.191747000	3.738785000
H	3.626059000	-2.937338000	4.446072000
C	5.506769000	0.157772000	-0.092414000
N	5.733644000	0.972867000	-1.064076000
C	7.868710000	0.527504000	-0.052196000

H	8.456831000	-0.233553000	-0.588217000
H	8.570832000	1.089698000	0.582080000
C	7.131655000	1.436767000	-1.072820000
C	6.980913000	-1.667560000	0.914958000
H	7.937553000	-1.859375000	1.429111000
H	6.179440000	-2.149525000	1.492212000
C	6.585411000	0.475227000	2.163732000
H	7.481656000	0.291776000	2.778893000
H	5.721803000	0.049511000	2.695468000
H	6.434581000	1.562407000	2.098244000
C	7.121386000	2.915338000	-0.651495000
H	8.136170000	3.343327000	-0.691872000
H	6.736705000	3.028559000	0.373066000
H	6.463971000	3.494910000	-1.316981000
C	7.699987000	1.301165000	-2.488336000
H	8.760128000	1.601210000	-2.515704000
H	7.142800000	1.935285000	-3.194905000
O	-1.631151000	-0.186766000	0.064819000
C	-2.864527000	-0.569162000	-0.025420000
B	-4.045415000	0.389063000	0.137558000
K	-0.260681000	2.021072000	-0.120204000
C	-3.003954000	-2.050602000	-0.324007000
C	-3.105792000	-2.991852000	0.727425000
C	-3.042017000	-4.361748000	0.426964000
H	-3.108862000	-5.096893000	1.231341000
C	-2.890884000	-4.801874000	-0.887717000
H	-2.836813000	-5.871552000	-1.104999000
C	-2.827771000	-3.869868000	-1.925445000
H	-2.737606000	-4.220108000	-2.956370000
C	-2.894310000	-2.494575000	-1.663768000
C	-3.697109000	1.921085000	0.402646000
C	-3.675413000	2.823780000	-0.691145000
C	-3.188966000	4.132269000	-0.526933000
H	-3.155743000	4.797514000	-1.392484000

C	-2.715623000	4.591688000	0.709839000
C	-2.776605000	3.708849000	1.797863000
H	-2.416770000	4.055117000	2.772026000
C	-3.247040000	2.393179000	1.662516000
C	-2.826065000	-1.490672000	-2.805710000
H	-3.177537000	-0.535332000	-2.396517000
C	-3.769253000	-1.844718000	-3.959581000
H	-3.471432000	-2.771504000	-4.476669000
H	-3.772382000	-1.037711000	-4.710402000
H	-4.792073000	-1.963045000	-3.576415000
C	-1.382128000	-1.289654000	-3.289723000
H	-0.934023000	-2.237281000	-3.633379000
H	-0.753755000	-0.879126000	-2.485171000
H	-1.336943000	-0.574818000	-4.126444000
C	-3.222744000	-2.529471000	2.174711000
H	-3.664969000	-1.523087000	2.147157000
C	-1.833052000	-2.399649000	2.819871000
H	-1.906750000	-2.087229000	3.873573000
H	-1.230215000	-1.642665000	2.300200000
H	-1.295579000	-3.362912000	2.792367000
C	-4.144049000	-3.410322000	3.025471000
H	-4.328541000	-2.937243000	4.002882000
H	-3.704059000	-4.401510000	3.221449000
H	-5.115543000	-3.565929000	2.532400000
C	-4.070320000	2.352128000	-2.087635000
H	-4.525227000	1.356081000	-1.982012000
C	-5.114321000	3.256856000	-2.753519000
H	-5.428262000	2.837436000	-3.722877000
H	-4.722318000	4.269567000	-2.942697000
H	-6.008968000	3.357440000	-2.119772000
C	-2.822877000	2.185642000	-2.971670000
H	-2.105975000	1.489724000	-2.509960000
H	-2.313602000	3.150654000	-3.133182000
H	-3.085854000	1.774432000	-3.959344000

C	-2.040761000	5.946469000	0.870494000
H	-2.162879000	6.242949000	1.927018000
C	-0.529376000	5.809718000	0.605249000
H	-0.067848000	5.064667000	1.272748000
H	-0.002131000	6.764914000	0.761050000
H	-0.339669000	5.493331000	-0.434442000
C	-2.650582000	7.052698000	0.003672000
H	-2.474807000	6.870816000	-1.068833000
H	-2.197461000	8.026971000	0.245506000
H	-3.737600000	7.130486000	0.158281000
C	-3.201182000	1.468527000	2.873338000
H	-3.639691000	0.511255000	2.553527000
C	-1.754384000	1.177297000	3.299875000
H	-1.723382000	0.475834000	4.148416000
H	-1.232788000	2.097364000	3.610271000
H	-1.195844000	0.720709000	2.470277000
C	-4.038704000	1.995229000	4.046245000
H	-4.058341000	1.264063000	4.870672000
H	-5.077464000	2.189607000	3.738658000
H	-3.627415000	2.936082000	4.446711000
C	-5.506752000	-0.157622000	-0.093083000
N	-5.733047000	-0.972012000	-1.065445000
C	-7.868589000	-0.528105000	-0.053825000
H	-8.457038000	0.233108000	-0.589262000
H	-8.570507000	-1.091196000	0.579879000
C	-6.746953000	0.156493000	0.768584000
C	-7.130959000	-1.436190000	-1.075119000
C	-6.981701000	1.666624000	0.915004000
H	-7.938433000	1.857856000	1.429199000
H	-7.019552000	2.158481000	-0.069628000
H	-6.180410000	2.148476000	1.492620000
C	-6.585795000	-0.476845000	2.162498000
H	-7.482239000	-0.294170000	2.777599000
H	-5.722456000	-0.051115000	2.694680000

H	-6.434499000	-1.563918000	2.096300000
C	-7.120519000	-2.915163000	-0.655230000
H	-8.135227000	-3.343278000	-0.696220000
H	-6.736003000	-3.029292000	0.369296000
H	-6.462888000	-3.494007000	-1.321135000
C	-7.698725000	-1.299273000	-2.490726000
H	-8.758699000	-1.599830000	-2.518950000
H	-7.140842000	-1.932289000	-3.197738000
H	-7.624986000	-0.255942000	-2.835734000
H	4.329363000	2.937212000	4.001141000
H	3.704688000	4.401475000	3.219799000
C	6.746630000	-0.157259000	0.769443000
H	7.018691000	-2.158823000	-0.069972000
H	7.625874000	0.258260000	-2.834554000

[**3**]$^{\bullet-}$ (doublet)

O	-0.027472000	0.718804000	-2.183947000
C	0.631588000	0.397590000	-1.117150000
B	-0.049186000	0.064687000	0.211381000
C	2.133337000	0.372480000	-1.334980000
C	2.914886000	1.529174000	-1.104552000
C	4.263627000	1.531534000	-1.494245000
H	4.873531000	2.422224000	-1.331074000
C	4.839900000	0.411078000	-2.092092000
H	5.889641000	0.430411000	-2.395423000
C	4.074025000	-0.740505000	-2.283869000
H	4.537172000	-1.625221000	-2.727010000
C	2.726171000	-0.781366000	-1.901801000
C	-1.641965000	0.101024000	0.232432000
C	-2.362831000	-1.107798000	0.057043000
C	-3.757036000	-1.087864000	-0.120548000
H	-4.283788000	-2.030684000	-0.283165000
C	-4.480992000	0.112373000	-0.127388000
C	-3.769592000	1.301877000	0.086908000

H	-4.321854000	2.247056000	0.090950000
C	-2.376147000	1.314736000	0.256483000
C	1.904616000	-2.044569000	-2.116012000
H	1.004391000	-1.934178000	-1.498736000
C	2.621016000	-3.305052000	-1.621714000
H	3.521073000	-3.536699000	-2.214371000
H	1.950026000	-4.176774000	-1.691516000
H	2.910156000	-3.175934000	-0.569726000
C	1.455520000	-2.179706000	-3.578772000
H	2.317410000	-2.205758000	-4.266779000
H	0.805188000	-1.340372000	-3.867312000
H	0.874266000	-3.102173000	-3.734757000
C	2.290847000	2.779557000	-0.496895000
H	1.423254000	2.443445000	0.089135000
C	1.763479000	3.715777000	-1.596775000
H	1.330243000	4.633898000	-1.169356000
H	0.971620000	3.226474000	-2.179922000
H	2.575866000	4.015087000	-2.280840000
C	3.226563000	3.526777000	0.459477000
H	2.673792000	4.312907000	0.997641000
H	4.056249000	4.018856000	-0.073368000
H	3.663704000	2.845227000	1.204809000
C	-1.622163000	-2.436981000	-0.058136000
H	-0.574446000	-2.255173000	0.223740000
C	-2.165453000	-3.510896000	0.892335000
H	-1.553512000	-4.425336000	0.833592000
H	-3.203223000	-3.791193000	0.648399000
H	-2.150710000	-3.157205000	1.934850000
C	-1.617577000	-2.925221000	-1.516549000
H	-1.173001000	-2.168865000	-2.181142000
H	-2.639751000	-3.139856000	-1.871207000
H	-1.022719000	-3.845906000	-1.627166000
C	-5.961788000	0.167486000	-0.475084000
H	-6.369070000	1.078444000	-0.002502000

C	-6.133238000	0.326043000	-1.997850000
H	-5.613166000	1.222342000	-2.371802000
H	-7.194870000	0.418200000	-2.279101000
H	-5.724384000	-0.547930000	-2.532687000
C	-6.768485000	-1.026556000	0.045020000
H	-6.481547000	-1.961711000	-0.462321000
H	-7.843932000	-0.875445000	-0.138243000
H	-6.620222000	-1.170810000	1.126116000
C	-1.665743000	2.655803000	0.398077000
H	-0.599216000	2.431080000	0.549579000
C	-1.769515000	3.482296000	-0.892724000
H	-1.220061000	4.432500000	-0.801916000
H	-2.817515000	3.726132000	-1.131881000
H	-1.337155000	2.927432000	-1.737626000
C	-2.151224000	3.451730000	1.616815000
H	-1.558004000	4.371964000	1.743009000
H	-2.062325000	2.859924000	2.540545000
H	-3.207149000	3.749395000	1.511247000
C	0.839335000	-0.414413000	1.423276000
N	1.812644000	-1.231668000	1.211915000
C	1.722253000	-0.900170000	3.591597000
H	1.196260000	-1.706250000	4.126360000
H	2.333592000	-0.365858000	4.334638000
C	0.693917000	0.026759000	2.894170000
C	2.575940000	-1.502031000	2.442371000
C	-0.729821000	-0.192034000	3.425314000
H	-0.771340000	0.015358000	4.507702000
H	-1.056796000	-1.231303000	3.264496000
H	-1.456088000	0.462496000	2.923322000
C	1.071719000	1.512881000	3.031257000
H	1.010644000	1.833125000	4.084366000
H	0.391962000	2.150917000	2.447609000
H	2.095038000	1.703009000	2.676760000
C	3.940270000	-0.809610000	2.291005000

H	4.586962000	-1.020010000	3.158250000
H	3.819334000	0.280472000	2.203667000
H	4.444441000	-1.160137000	1.377819000
C	2.771302000	-3.013326000	2.593677000
H	3.303169000	-3.248219000	3.529953000
H	3.356127000	-3.417257000	1.753070000
H	1.797495000	-3.527564000	2.609849000

4-H (closed-shell singlet)

B	-0.796968000	3.997349000	0.069012000
C	0.319654000	2.987227000	0.266073000
O	0.087692000	1.708456000	0.197681000
K	-1.973477000	0.161387000	0.257376000
C	-2.249884000	3.349361000	-0.243217000
C	-3.238657000	3.246540000	0.770012000
C	-4.482186000	2.643365000	0.502967000
H	-5.224039000	2.553576000	1.302098000
C	-4.795585000	2.127375000	-0.759686000
C	-3.829623000	2.249071000	-1.771452000
H	-4.053179000	1.857675000	-2.765984000
C	-2.580780000	2.842968000	-1.532076000
C	-2.925298000	3.704031000	2.190521000
H	-2.054498000	4.370771000	2.113926000
C	-2.492827000	2.501293000	3.045150000
H	-2.203184000	2.812087000	4.062371000
H	-1.622109000	2.001675000	2.595875000
H	-3.309783000	1.764703000	3.140789000
C	-4.062135000	4.489284000	2.852448000
H	-3.738441000	4.887558000	3.827807000
H	-4.953019000	3.866067000	3.035857000
H	-4.368897000	5.340028000	2.224029000
C	-6.106346000	1.393275000	-0.999756000
H	-6.746086000	1.581939000	-0.120160000
C	-5.867826000	-0.124400000	-1.078450000

H	-6.814232000	-0.673963000	-1.211353000
H	-5.386100000	-0.506404000	-0.164859000
H	-5.213100000	-0.376235000	-1.929186000
C	-6.857995000	1.901159000	-2.237272000
H	-7.835539000	1.401642000	-2.332772000
H	-6.293820000	1.698914000	-3.161968000
H	-7.030410000	2.986991000	-2.180769000
C	-1.568412000	2.963805000	-2.664032000
H	-0.585187000	2.969150000	-2.179524000
C	-1.720421000	4.314352000	-3.379825000
H	-0.938774000	4.445662000	-4.146753000
H	-1.636739000	5.143312000	-2.663224000
H	-2.701847000	4.388505000	-3.878039000
C	-1.586039000	1.799877000	-3.658821000
H	-0.754162000	1.897893000	-4.373957000
H	-2.515422000	1.762712000	-4.250334000
H	-1.472028000	0.830391000	-3.149676000
C	-0.776170000	5.497004000	0.126420000
N	-1.944104000	6.178841000	-0.101637000
H	-2.766479000	5.673305000	-0.414546000
C	-1.882687000	7.630926000	-0.199140000
C	-0.534025000	7.893827000	0.496920000
H	-0.721218000	8.136957000	1.553912000
H	0.003793000	8.746491000	0.056913000
C	0.288828000	6.572099000	0.412617000
C	-1.890822000	8.085009000	-1.670684000
H	-1.826892000	9.182476000	-1.744956000
H	-2.819817000	7.763609000	-2.168650000
H	-1.045298000	7.651457000	-2.222627000
C	-3.065639000	8.261518000	0.544557000
H	-2.994390000	9.360709000	0.538126000
H	-3.091153000	7.916815000	1.589302000
H	-4.020976000	7.985202000	0.068107000
C	1.021338000	6.351466000	1.742968000

H	1.645932000	7.229187000	1.981394000
H	1.677389000	5.476496000	1.697646000
H	0.305426000	6.205992000	2.566233000
C	1.323179000	6.635036000	-0.725773000
H	1.996422000	7.497241000	-0.584716000
H	0.844459000	6.729882000	-1.711205000
H	1.935952000	5.726961000	-0.733503000
C	1.798549000	3.217851000	0.528595000
C	2.287369000	3.113858000	1.857064000
C	3.671925000	3.110111000	2.080732000
H	4.054967000	3.033437000	3.100398000
C	4.570841000	3.206705000	1.018228000
H	5.647352000	3.203500000	1.206444000
C	4.089227000	3.312143000	-0.286546000
H	4.800533000	3.384031000	-1.110847000
C	2.711415000	3.314757000	-0.553978000
C	1.323179000	2.957741000	3.029022000
H	0.359522000	3.372507000	2.696340000
C	1.752606000	3.724649000	4.285457000
H	0.948248000	3.697273000	5.037833000
H	1.973957000	4.778444000	4.061254000
H	2.647110000	3.282894000	4.753999000
C	1.095714000	1.472321000	3.355139000
H	0.403607000	1.352123000	4.203267000
H	2.043224000	0.973614000	3.622211000
H	0.653211000	0.957052000	2.493041000
C	2.212459000	3.346574000	-1.996665000
H	1.229264000	3.839160000	-1.974030000
C	1.995389000	1.919924000	-2.533491000
H	1.665456000	1.938295000	-3.583749000
H	1.218598000	1.402421000	-1.954181000
H	2.931640000	1.337196000	-2.491032000
C	3.111068000	4.142148000	-2.949501000
H	2.614281000	4.257615000	-3.925692000

H	4.071239000	3.633423000	-3.134389000
H	3.330517000	5.147126000	-2.558825000
B	0.796968000	-3.997349000	0.069012000
C	-0.319654000	-2.987227000	0.266073000
O	-0.087692000	-1.708456000	0.197681000
K	1.973477000	-0.161387000	0.257376000
C	2.249884000	-3.349361000	-0.243217000
C	3.238657000	-3.246540000	0.770012000
C	4.482186000	-2.643365000	0.502967000
H	5.224039000	-2.553576000	1.302098000
C	4.795585000	-2.127375000	-0.759686000
C	3.829623000	-2.249071000	-1.771452000
H	4.053179000	-1.857675000	-2.765984000
C	2.580780000	-2.842968000	-1.532076000
C	2.925298000	-3.704031000	2.190521000
H	2.054498000	-4.370771000	2.113926000
C	2.492827000	-2.501293000	3.045150000
H	2.203184000	-2.812087000	4.062371000
H	1.622109000	-2.001675000	2.595875000
H	3.309783000	-1.764703000	3.140789000
C	4.062135000	-4.489284000	2.852448000
H	3.738441000	-4.887558000	3.827807000
H	4.953019000	-3.866067000	3.035857000
H	4.368897000	-5.340028000	2.224029000
C	6.106346000	-1.393275000	-0.999756000
H	6.746086000	-1.581939000	-0.120160000
C	5.867826000	0.124400000	-1.078450000
H	6.814232000	0.673963000	-1.211353000
H	5.386100000	0.506404000	-0.164859000
H	5.213100000	0.376235000	-1.929186000
C	6.857995000	-1.901159000	-2.237272000
H	7.835539000	-1.401642000	-2.332772000
H	6.293820000	-1.698914000	-3.161968000
H	7.030410000	-2.986991000	-2.180769000

C	1.568412000	-2.963805000	-2.664032000
H	0.585187000	-2.969150000	-2.179524000
C	1.720421000	-4.314352000	-3.379825000
H	0.938774000	-4.445662000	-4.146753000
H	1.636739000	-5.143312000	-2.663224000
H	2.701847000	-4.388505000	-3.878039000
C	1.586039000	-1.799877000	-3.658821000
H	0.754162000	-1.897893000	-4.373957000
H	2.515422000	-1.762712000	-4.250334000
H	1.472028000	-0.830391000	-3.149676000
C	0.776170000	-5.497004000	0.126420000
N	1.944104000	-6.178841000	-0.101637000
H	2.766479000	-5.673305000	-0.414546000
C	1.882687000	-7.630926000	-0.199140000
C	0.534025000	-7.893827000	0.496920000
H	0.721218000	-8.136957000	1.553912000
H	-0.003793000	-8.746491000	0.056913000
C	-0.288828000	-6.572099000	0.412617000
C	1.890822000	-8.085009000	-1.670684000
H	1.826892000	-9.182476000	-1.744956000
H	2.819817000	-7.763609000	-2.168650000
H	1.045298000	-7.651457000	-2.222627000
C	3.065639000	-8.261518000	0.544557000
H	2.994390000	-9.360709000	0.538126000
H	3.091153000	-7.916815000	1.589302000
H	4.020976000	-7.985202000	0.068107000
C	-1.021338000	-6.351466000	1.742968000
H	-1.645932000	-7.229187000	1.981394000
H	-1.677389000	-5.476496000	1.697646000
H	-0.305426000	-6.205992000	2.566233000
C	-1.323179000	-6.635036000	-0.725773000
H	-1.996422000	-7.497241000	-0.584716000
H	-0.844459000	-6.729882000	-1.711205000
H	-1.935952000	-5.726961000	-0.733503000

C	-1.798549000	-3.217851000	0.528595000
C	-2.287369000	-3.113858000	1.857064000
C	-3.671925000	-3.110111000	2.080732000
H	-4.054967000	-3.033437000	3.100398000
C	-4.570841000	-3.206705000	1.018228000
H	-5.647352000	-3.203500000	1.206444000
C	-4.089227000	-3.312143000	-0.286546000
H	-4.800533000	-3.384031000	-1.110847000
C	-2.711415000	-3.314757000	-0.553978000
C	-1.323179000	-2.957741000	3.029022000
H	-0.359522000	-3.372507000	2.696340000
C	-1.752606000	-3.724649000	4.285457000
H	-0.948248000	-3.697273000	5.037833000
H	-1.973957000	-4.778444000	4.061254000
H	-2.647110000	-3.282894000	4.753999000
C	-1.095714000	-1.472321000	3.355139000
H	-0.403607000	-1.352123000	4.203267000
H	-2.043224000	-0.973614000	3.622211000
H	-0.653211000	-0.957052000	2.493041000
C	-2.212459000	-3.346574000	-1.996665000
H	-1.229264000	-3.839160000	-1.974030000
C	-1.995389000	-1.919924000	-2.533491000
H	-1.665456000	-1.938295000	-3.583749000
H	-1.218598000	-1.402421000	-1.954181000
H	-2.931640000	-1.337196000	-2.491032000
C	-3.111068000	-4.142148000	-2.949501000
H	-2.614281000	-4.257615000	-3.925692000
H	-4.071239000	-3.633423000	-3.134389000
H	-3.330517000	-5.147126000	-2.558825000

4-H (triplet)

B	-0.241194000	4.081694000	0.018750000
C	0.703359000	2.908849000	0.338168000
O	0.241465000	1.681848000	0.394324000

K	-1.942565000	0.346716000	0.436226000
C	-1.762054000	3.641500000	-0.289946000
C	-2.745162000	3.691443000	0.731145000
C	-4.053541000	3.236876000	0.484966000
H	-4.795700000	3.259570000	1.288806000
C	-4.434901000	2.728379000	-0.763055000
C	-3.470272000	2.708731000	-1.783021000
H	-3.746874000	2.323856000	-2.767011000
C	-2.156804000	3.153615000	-1.565276000
C	-2.363225000	4.146145000	2.135654000
H	-1.390400000	4.653148000	2.043087000
C	-2.148353000	2.928464000	3.049541000
H	-1.813534000	3.234096000	4.054256000
H	-1.374851000	2.266991000	2.632606000
H	-3.080687000	2.349556000	3.170127000
C	-3.351467000	5.147593000	2.742813000
H	-2.979916000	5.519169000	3.711619000
H	-4.342445000	4.699391000	2.923475000
H	-3.488808000	6.013349000	2.076302000
C	-5.826283000	2.150813000	-0.975958000
H	-6.434325000	2.448320000	-0.104010000
C	-5.774569000	0.613583000	-0.998241000
H	-6.781175000	0.178686000	-1.111497000
H	-5.340869000	0.208529000	-0.070540000
H	-5.158406000	0.252556000	-1.838178000
C	-6.516750000	2.699847000	-2.231302000
H	-7.548969000	2.321587000	-2.305967000
H	-5.987827000	2.393856000	-3.148266000
H	-6.553523000	3.800021000	-2.216891000
C	-1.145509000	3.127408000	-2.704575000
H	-0.157887000	3.122112000	-2.224599000
C	-1.232715000	4.414391000	-3.539631000
H	-0.456106000	4.431042000	-4.322311000
H	-1.093526000	5.301598000	-2.905281000

H	-2.215248000	4.498197000	-4.034057000
C	-1.235342000	1.880062000	-3.589401000
H	-0.418334000	1.873428000	-4.327761000
H	-2.180420000	1.833691000	-4.154587000
H	-1.152507000	0.958603000	-2.993756000
C	-0.007487000	5.587810000	-0.004988000
N	-1.059994000	6.405628000	-0.350690000
H	-1.926230000	6.005218000	-0.694496000
C	-0.791854000	7.831973000	-0.465642000
C	0.520870000	7.931396000	0.338196000
H	0.281086000	8.217259000	1.373760000
H	1.198159000	8.699268000	-0.063813000
C	1.167060000	6.512351000	0.336216000
C	-0.618023000	8.241470000	-1.940523000
H	-0.379112000	9.313734000	-2.026167000
H	-1.545313000	8.053236000	-2.505333000
H	0.189611000	7.667711000	-2.415847000
C	-1.933050000	8.638226000	0.165043000
H	-1.711986000	9.717087000	0.141411000
H	-2.087235000	8.334497000	1.211453000
H	-2.876579000	8.477977000	-0.382751000
C	1.765753000	6.218492000	1.720407000
H	2.506102000	6.990968000	1.990102000
H	2.274349000	5.248422000	1.732614000
H	0.982370000	6.208238000	2.493777000
C	2.288521000	6.415592000	-0.716905000
H	3.059399000	7.182658000	-0.530364000
H	1.905564000	6.554753000	-1.738311000
H	2.774209000	5.434253000	-0.667070000
C	2.190123000	2.940525000	0.614200000
C	2.650816000	2.792846000	1.952132000
C	4.021546000	2.622025000	2.194427000
H	4.379966000	2.508168000	3.219883000
C	4.940188000	2.606879000	1.144415000

H	6.005775000	2.473649000	1.347207000
C	4.492385000	2.771093000	-0.167146000
H	5.218969000	2.755699000	-0.980955000
C	3.128716000	2.936314000	-0.452717000
C	1.664134000	2.795720000	3.115288000
H	0.762564000	3.312618000	2.752123000
C	2.173934000	3.555464000	4.346473000
H	1.366110000	3.657920000	5.088717000
H	2.525258000	4.564039000	4.083092000
H	3.003531000	3.027096000	4.844009000
C	1.246512000	1.366438000	3.497934000
H	0.540241000	1.370585000	4.343193000
H	2.120825000	0.761993000	3.795700000
H	0.744424000	0.879238000	2.652373000
C	2.650816000	3.014920000	-1.900114000
H	1.768495000	3.672362000	-1.902486000
C	2.187890000	1.631823000	-2.391909000
H	1.864873000	1.672139000	-3.443292000
H	1.335049000	1.271223000	-1.799672000
H	3.010052000	0.898435000	-2.328850000
C	3.677112000	3.609468000	-2.869337000
H	3.212591000	3.773083000	-3.854451000
H	4.536490000	2.936940000	-3.025472000
H	4.063936000	4.575550000	-2.511445000
B	0.241194000	-4.081694000	0.018750000
C	-0.703359000	-2.908849000	0.338168000
O	-0.241465000	-1.681848000	0.394324000
K	1.942565000	-0.346716000	0.436226000
C	1.762054000	-3.641500000	-0.289946000
C	2.745162000	-3.691443000	0.731145000
C	4.053541000	-3.236876000	0.484966000
H	4.795700000	-3.259570000	1.288806000
C	4.434901000	-2.728379000	-0.763055000
C	3.470272000	-2.708731000	-1.783021000

H	3.746874000	-2.323856000	-2.767011000
C	2.156804000	-3.153615000	-1.565276000
C	2.363225000	-4.146145000	2.135654000
H	1.390400000	-4.653148000	2.043087000
C	2.148353000	-2.928464000	3.049541000
H	1.813534000	-3.234096000	4.054256000
H	1.374851000	-2.266991000	2.632606000
H	3.080687000	-2.349556000	3.170127000
C	3.351467000	-5.147593000	2.742813000
H	2.979916000	-5.519169000	3.711619000
H	4.342445000	-4.699391000	2.923475000
H	3.488808000	-6.013349000	2.076302000
C	5.826283000	-2.150813000	-0.975958000
H	6.434325000	-2.448320000	-0.104010000
C	5.774569000	-0.613583000	-0.998241000
H	6.781175000	-0.178686000	-1.111497000
H	5.340869000	-0.208529000	-0.070540000
H	5.158406000	-0.252556000	-1.838178000
C	6.516750000	-2.699847000	-2.231302000
H	7.548969000	-2.321587000	-2.305967000
H	5.987827000	-2.393856000	-3.148266000
H	6.553523000	-3.800021000	-2.216891000
C	1.145509000	-3.127408000	-2.704575000
H	0.157887000	-3.122112000	-2.224599000
C	1.232715000	-4.414391000	-3.539631000
H	0.456106000	-4.431042000	-4.322311000
H	1.093526000	-5.301598000	-2.905281000
H	2.215248000	-4.498197000	-4.034057000
C	1.235342000	-1.880062000	-3.589401000
H	0.418334000	-1.873428000	-4.327761000
H	2.180420000	-1.833691000	-4.154587000
H	1.152507000	-0.958603000	-2.993756000
C	0.007487000	-5.587810000	-0.004988000
N	1.059994000	-6.405628000	-0.350690000

H	1.926230000	-6.005218000	-0.694496000
C	0.791854000	-7.831973000	-0.465642000
C	-0.520870000	-7.931396000	0.338196000
H	-0.281086000	-8.217259000	1.373760000
H	-1.198159000	-8.699268000	-0.063813000
C	-1.167060000	-6.512351000	0.336216000
C	0.618023000	-8.241470000	-1.940523000
H	0.379112000	-9.313734000	-2.026167000
H	1.545313000	-8.053236000	-2.505333000
H	-0.189611000	-7.667711000	-2.415847000
C	1.933050000	-8.638226000	0.165043000
H	1.711986000	-9.717087000	0.141411000
H	2.087235000	-8.334497000	1.211453000
H	2.876579000	-8.477977000	-0.382751000
C	-1.765753000	-6.218492000	1.720407000
H	-2.506102000	-6.990968000	1.990102000
H	-2.274349000	-5.248422000	1.732614000
H	-0.982370000	-6.208238000	2.493777000
C	-2.288521000	-6.415592000	-0.716905000
H	-3.059399000	-7.182658000	-0.530364000
H	-1.905564000	-6.554753000	-1.738311000
H	-2.774209000	-5.434253000	-0.667070000
C	-2.190123000	-2.940525000	0.614200000
C	-2.650816000	-2.792846000	1.952132000
C	-4.021546000	-2.622025000	2.194427000
H	-4.379966000	-2.508168000	3.219883000
C	-4.940188000	-2.606879000	1.144415000
H	-6.005775000	-2.473649000	1.347207000
C	-4.492385000	-2.771093000	-0.167146000
H	-5.218969000	-2.755699000	-0.980955000
C	-3.128716000	-2.936314000	-0.452717000
C	-1.664134000	-2.795720000	3.115288000
H	-0.762564000	-3.312618000	2.752123000
C	-2.173934000	-3.555464000	4.346473000

H	-1.366110000	-3.657920000	5.088717000
H	-2.525258000	-4.564039000	4.083092000
H	-3.003531000	-3.027096000	4.844009000
C	-1.246512000	-1.366438000	3.497934000
H	-0.540241000	-1.370585000	4.343193000
H	-2.120825000	-0.761993000	3.795700000
H	-0.744424000	-0.879238000	2.652373000
C	-2.650816000	-3.014920000	-1.900114000
H	-1.768495000	-3.672362000	-1.902486000
C	-2.187890000	-1.631823000	-2.391909000
H	-1.864873000	-1.672139000	-3.443292000
H	-1.335049000	-1.271223000	-1.799672000
H	-3.010052000	-0.898435000	-2.328850000
C	-3.677112000	-3.609468000	-2.869337000
H	-3.212591000	-3.773083000	-3.854451000
H	-4.536490000	-2.936940000	-3.025472000
H	-4.063936000	-4.575550000	-2.511445000

**4-K**			
C	-4.940178553	-2.723076013	-0.577067361
N	-6.120306420	-2.136594546	-0.398083882
C	-7.250333690	-3.039219593	-0.640324982
C	-6.631491537	-4.453783142	-0.673073640
H	-7.084164467	-5.102777137	-1.439894422
H	-6.792092916	-4.946348125	0.299622122
C	-5.114993284	-4.234719376	-0.902182152
C	-8.289258493	-2.898918363	0.482826898
H	-9.121930166	-3.610950456	0.353111351
H	-8.730648835	-1.886574880	0.500991121
H	-7.826230675	-3.088206801	1.464132190
C	-7.916073383	-2.667701582	-1.979046530
H	-8.777974109	-3.320841211	-2.200017455
H	-7.199188762	-2.749559487	-2.809901802
H	-8.277432969	-1.626060878	-1.952216211

C	-4.716053515	-4.522982206	-2.358910740
H	-4.925018789	-5.574621487	-2.624063123
H	-3.643850882	-4.346266480	-2.508325958
H	-5.265435127	-3.881009789	-3.065083279
C	-4.310640928	-5.150197531	0.030707085
H	-4.601563858	-6.205087442	-0.121223323
H	-4.494483074	-4.898512121	1.087477726
H	-3.234751476	-5.074657240	-0.157637887
C	-1.471676215	-3.471346756	-1.047460268
C	-1.114269081	-3.738305544	-2.398130426
C	-0.274849706	-4.824299250	-2.695408086
H	-0.002813138	-5.029036991	-3.733726568
C	0.225945225	-5.645708301	-1.685180677
H	0.878245788	-6.488536420	-1.930731885
C	-0.111805572	-5.381752202	-0.356103201
H	0.286857495	-6.025104205	0.430736757
C	-0.948015792	-4.306129460	-0.019676350
C	-1.565131679	-2.810345258	-3.521831467
H	-2.468473561	-2.299092247	-3.156721607
C	-1.923349995	-3.537870311	-4.822244291
H	-2.384841610	-2.834380107	-5.534897050
H	-2.637714526	-4.357801808	-4.647777081
H	-1.036109929	-3.964029344	-5.319702889
C	-0.504753721	-1.726429355	-3.781534420
H	-0.809303364	-1.060000176	-4.604450382
H	0.465326637	-2.175400659	-4.056761322
H	-0.374927623	-1.112589022	-2.880824613
C	-1.246319135	-4.006892150	1.446916996
H	-2.238480724	-3.532806185	1.465570917
C	-1.298451085	-5.250097840	2.339590246
H	-1.675928251	-4.981166649	3.339858079
H	-0.303074662	-5.703239499	2.482191359
H	-1.964132865	-6.023613618	1.924788918
C	-0.251461707	-2.975003897	2.008252531

H	-0.454448348	-2.767432634	3.071458225
H	-0.343611791	-2.030696378	1.454263383
H	0.785917254	-3.344927840	1.934621026
C	-3.889600992	-0.310497886	-0.162520789
C	-4.309167630	0.603568193	-1.165247196
C	-4.492681432	1.969133532	-0.880107169
H	-4.813086545	2.640642252	-1.679301274
C	-4.280861722	2.489003749	0.403233098
C	-3.862574582	1.593314485	1.398940649
H	-3.690077933	1.983603519	2.405137484
C	-3.661526503	0.228386494	1.135482855
C	-4.528000642	0.128031338	-2.594679879
H	-4.512135972	-0.970073614	-2.549863842
C	-5.878235346	0.562788582	-3.173373958
H	-6.039979960	0.108090476	-4.164821005
H	-5.947036273	1.656368367	-3.300738217
H	-6.708200542	0.240210383	-2.526148018
C	-3.361508578	0.570554047	-3.489377603
H	-3.472694173	0.180817017	-4.514938839
H	-2.405518931	0.192057202	-3.100316483
H	-3.299418004	1.670790982	-3.555389448
C	-3.233953101	-0.696865322	2.265691320
H	-2.700715083	-1.521315618	1.777004106
C	-4.472875529	-1.297328441	2.947567358
H	-4.188090823	-2.023117697	3.728047464
H	-5.103027872	-1.822305229	2.213395119
H	-5.079623389	-0.510270190	3.426601957
C	-2.288936966	-0.056265121	3.284105006
H	-1.923158496	-0.814994189	3.994782374
H	-2.777830930	0.733035889	3.880130376
H	-1.409332690	0.389419885	2.793687139
C	-6.047625671	4.136000757	1.067958123
H	-6.266585829	5.182387520	1.339037705
H	-6.675626771	3.864769927	0.204227438

B	-3.641317642	-1.878093428	-0.492672832
C	-2.188877559	-2.180124608	-0.695779757
O	-1.262525882	-1.223084301	-0.588039449
K	-6.740187137	0.309608518	0.644187749
C	4.923765068	2.693365388	-0.470861064
N	6.110839280	2.104804207	-0.355624855
C	7.231045021	3.030692902	-0.549117267
C	6.602762927	4.439405036	-0.470853925
H	7.037086424	5.143935078	-1.198204202
H	6.778232600	4.863806110	0.530981278
C	5.083364397	4.225858377	-0.687571779
C	7.878042314	2.760535248	-1.920617138
H	8.733653768	3.432490498	-2.106910660
H	7.148244762	2.897369298	-2.732797184
H	8.244257842	1.721458518	-1.973217838
C	8.288850074	2.816413481	0.544785491
H	9.115158231	3.541721128	0.454391877
H	8.736738024	1.808617922	0.481405711
H	7.841515907	2.929666133	1.544829556
C	4.653503944	4.618581473	-2.111080169
H	4.852518495	5.688104342	-2.301651634
H	3.579367147	4.447190863	-2.250065092
H	5.190767539	4.033286215	-2.873504234
C	4.291427736	5.062233376	0.326235687
H	4.570622684	6.128261065	0.248020973
H	4.498696923	4.734079392	1.357460196
H	3.212296585	4.990560148	0.154457700
C	1.442425239	3.450126314	-0.826901212
C	0.918075155	4.194941779	0.267921091
C	0.058594077	5.277408217	0.025328915
H	-0.346672878	5.847099350	0.864499472
C	-0.295195286	5.640769490	-1.275846792
H	-0.963246175	6.488965635	-1.448624021
C	0.210663441	4.911630467	-2.352198280

H	-0.074682241	5.193615815	-3.368545069
C	1.067371440	3.817575661	-2.148889048
C	1.239679538	3.790575831	1.703802387
H	2.233264825	3.320161329	1.671808907
C	0.255002353	2.718637745	2.204066910
H	0.471945462	2.437025816	3.247336882
H	0.340780011	1.815662689	1.584074066
H	-0.784865371	3.089435577	2.169754925
C	1.305934297	4.966172052	2.683202597
H	1.701350783	4.626524699	3.654647082
H	0.312954031	5.405817437	2.875963152
H	1.963355086	5.769076081	2.313622587
C	1.515956820	2.985421360	-3.346073626
H	2.429573799	2.458713527	-3.031285665
C	1.849677070	3.815329790	-4.590565147
H	2.311850949	3.174770049	-5.359831087
H	2.555257910	4.629380885	-4.361739443
H	0.951236301	4.265937548	-5.044409807
C	0.463047849	1.913497947	-3.676730829
H	0.763356502	1.316544380	-4.552725380
H	-0.514303367	2.373009816	-3.904894490
H	0.349663461	1.229728047	-2.825601411
C	3.899160616	0.247586122	-0.224069791
C	3.703069880	-0.390903368	1.034546824
C	3.943503754	-1.764692675	1.199210364
H	3.799926928	-2.219767625	2.181210480
C	4.367707154	-2.574014015	0.132576444
C	4.535149748	-1.958974779	-1.113173054
H	4.853580268	-2.576328515	-1.958023561
C	4.315088425	-0.581270458	-1.299034116
C	3.269868022	0.442772795	2.232235491
H	2.716055593	1.287999623	1.805153150
C	2.348984211	-0.287954915	3.211232209
H	1.977736467	0.409957794	3.979047092

H	2.859773228	-1.108800537	3.742815177
H	1.471098226	-0.714076278	2.700696339
C	4.501462207	1.020622726	2.945751518
H	4.207630830	1.689573102	3.772210216
H	5.117772099	1.604284329	2.244899311
H	5.124976869	0.216545445	3.371958463
C	3.503787771	-4.812866339	0.951041855
H	3.725441975	-5.891144519	1.012384669
H	2.585231298	-4.693130514	0.355745788
H	3.291856395	-4.461167048	1.973856672
C	4.484153238	-0.003892010	-2.697094542
H	4.462883085	1.088194840	-2.575661360
C	3.287030330	-0.391738923	-3.577026543
H	3.354665374	0.074015090	-4.574231683
H	2.344878647	-0.054042386	-3.122633189
H	3.232074214	-1.484742898	-3.722158055
C	5.814338410	-0.383624736	-3.354782629
H	5.932016370	0.137610609	-4.319378010
H	5.890818334	-1.465366226	-3.556536599
H	6.665229391	-0.094151603	-2.718956225
B	3.631132006	1.834204970	-0.426424499
C	2.174271669	2.140895053	-0.583957953
O	1.256049350	1.172009933	-0.532991474
K	-1.337077119	1.348398993	-0.588590805
K	1.335619399	-1.397016285	-0.673797539
K	6.744765525	-0.369192118	0.574883484
O	-8.566224309	1.981453482	-0.257822050
C	-9.507833468	2.494515479	0.698814854
H	-9.125027070	3.443599701	1.118160391
H	-9.601959072	1.766762944	1.518931113
C	-10.792735838	2.720939592	-0.089015430
H	-11.451164682	3.466561554	0.379991517
H	-11.352270821	1.775931447	-0.186659930
C	-10.239912515	3.160486180	-1.449690403

H	-10.951685602	3.030947378	-2.277725943
H	-9.944347124	4.221694726	-1.413669720
C	-9.006002247	2.269243227	-1.597399823
H	-9.249538931	1.318214008	-2.102974640
H	-8.184509074	2.751483136	-2.149783077
O	-7.760282596	0.475506688	3.083673906
C	-7.485618066	1.557173277	3.990277800
H	-8.004779048	2.460284511	3.633440706
H	-6.399288666	1.759221633	3.992329886
C	-7.967435909	1.081831785	5.359567345
H	-7.416540591	1.554749545	6.185411309
H	-9.039215369	1.305511855	5.487337510
C	-7.748087759	-0.432013401	5.262754495
H	-8.354153563	-1.011199651	5.974328907
H	-6.687245546	-0.677983696	5.432078175
C	-8.128085973	-0.709453544	3.811758373
H	-7.598526816	-1.571020851	3.377357780
H	-9.214723248	-0.878610391	3.701697686
O	7.811332219	-0.552229009	2.994464025
C	8.117769345	0.577281377	3.832364916
H	7.512726917	1.437088264	3.506423201
H	9.184554430	0.835671309	3.707295408
C	7.809710979	0.127813649	5.258407219
H	8.415795198	0.659124718	6.006365298
H	6.746252619	0.300374777	5.490532311
C	8.100799424	-1.375416127	5.187091001
H	7.606195124	-1.957714649	5.977981077
H	9.185859222	-1.560101913	5.248839086
C	7.582332776	-1.724189247	3.795094079
H	8.101172071	-2.576107737	3.329006141
H	6.499566167	-1.945511393	3.814681628
O	8.625410085	-2.016662608	-0.285834216
C	9.777115472	-2.148497774	0.564325350
H	9.867169215	-3.197644313	0.903158024

H	9.629159735	-1.509056179	1.447586934
C	10.959609608	-1.746801362	-0.308452159
H	11.047665654	-0.648100321	-0.345025277
H	11.913415562	-2.159100311	0.051617258
C	10.529837828	-2.297220775	-1.673464004
H	10.726764421	-3.380360210	-1.724101086
H	11.038240584	-1.816855285	-2.521881510
C	9.023896323	-2.027536311	-1.670066231
H	8.786968958	-1.044499151	-2.113899689
H	8.441414574	-2.793334966	-2.206449081
C	-4.556282097	3.944125103	0.743729831
H	-3.984894914	4.171719274	1.660288777
H	-6.353742380	3.498630701	1.912681940
C	-4.093647247	4.918174691	-0.344211817
H	-3.032486448	4.765185638	-0.597116395
H	-4.212684706	5.960587074	-0.006665800
H	-4.681986247	4.805787085	-1.269352506
C	5.969808516	-4.234723028	1.133840976
H	6.819966745	-3.726919433	0.649987875
H	6.223783327	-5.303272411	1.234251666
H	5.859777114	-3.821406656	2.150110807
C	4.676449741	-4.049015120	0.324032014
H	4.850937541	-4.471295802	-0.680321712

**3a**			
O	-1.630268000	-0.186502000	0.065506000
C	-2.863477000	-0.569354000	-0.025156000
B	-4.044870000	0.388481000	0.136463000
K	-0.260869000	2.021896000	-0.116723000
C	-3.002337000	-2.051107000	-0.322499000
C	-3.104050000	-2.991383000	0.729828000
C	-3.039865000	-4.361555000	0.430707000
H	-3.106579000	-5.095933000	1.235801000
C	-2.888468000	-4.802911000	-0.883532000

H	-2.834079000	-5.872782000	-1.099771000
C	-2.825391000	-3.871883000	-1.922134000
H	-2.734897000	-4.223068000	-2.952710000
C	-2.892278000	-2.496343000	-1.661803000
C	-3.697567000	1.920895000	0.400631000
C	-3.674695000	2.822525000	-0.693981000
C	-3.189199000	4.131437000	-0.530283000
H	-3.155053000	4.795878000	-1.396409000
C	-2.717996000	4.592282000	0.706776000
C	-2.780199000	3.710465000	1.795571000
H	-2.422022000	4.057851000	2.769946000
C	-3.249737000	2.394424000	1.660756000
C	-5.505962000	-0.158895000	-0.094371000
N	-5.731507000	-0.974088000	-1.066223000
C	-7.867688000	-0.530229000	-0.055967000
H	-8.456146000	0.230385000	-0.592239000
H	-8.569707000	-1.093119000	0.577800000
C	-7.129253000	-1.438780000	-1.076246000
O	1.630356000	0.186745000	0.066182000
C	2.863567000	0.569526000	-0.024730000
B	4.044935000	-0.388400000	0.136578000
K	0.260875000	-2.021845000	-0.116430000
C	3.002395000	2.051317000	-0.321970000
C	3.103876000	2.991520000	0.730446000
C	3.039614000	4.361711000	0.431424000
H	3.106147000	5.096028000	1.236588000
C	2.888355000	4.803164000	-0.882796000
H	2.833909000	5.873047000	-1.098956000
C	2.825494000	3.872214000	-1.921481000
H	2.735109000	4.223479000	-2.952037000
C	2.892467000	2.496659000	-1.661255000
C	3.697582000	-1.920798000	0.400791000
C	3.674672000	-2.822521000	-0.693756000
C	3.189092000	-4.131386000	-0.529969000

H	3.154891000	-4.795879000	-1.396053000
C	2.717856000	-4.592123000	0.707119000
C	2.780126000	-3.710240000	1.795851000
H	2.421918000	-4.057532000	2.770248000
C	3.249736000	-2.394232000	1.660946000
C	5.506047000	0.158799000	-0.094554000
N	5.731543000	0.974072000	-1.066351000
C	7.867904000	0.529376000	-0.056819000
H	8.455937000	-0.231375000	-0.593363000
H	8.570308000	1.091956000	0.576794000
C	6.746952000	-0.155935000	0.765915000
C	7.129446000	1.438290000	-1.076767000
C	-6.746707000	0.155371000	0.766489000
H	-2.244233000	5.545030000	0.819558000
H	-3.950056000	2.490683000	-1.673244000
H	-3.218378000	1.746020000	2.511337000
H	-6.635048000	-0.284087000	1.735668000
H	-6.910784000	1.207864000	0.867667000
H	-7.121760000	-2.467864000	-0.783280000
H	-7.525575000	-1.343962000	-2.065609000
H	-3.186372000	-2.665849000	1.745776000
H	-2.844068000	-1.791435000	-2.465345000
H	3.186070000	2.665929000	1.746386000
H	2.844379000	1.791807000	-2.464854000
H	7.525410000	1.343399000	-2.066266000
H	7.122432000	2.467355000	-0.783720000
H	6.910755000	-1.208479000	0.867003000
H	6.635714000	0.283519000	1.735145000
H	3.949948000	-2.490743000	-1.673065000
H	2.244022000	-5.544831000	0.819939000
H	3.218471000	-1.745820000	2.511525000

**3b**			
O	-1.630268000	-0.186502000	0.065506000
C	-2.863477000	-0.569354000	-0.025156000
B	-4.044870000	0.388481000	0.136463000
K	-0.260869000	2.021896000	-0.116723000
C	-3.002337000	-2.051107000	-0.322499000
C	-3.104050000	-2.991383000	0.729828000
C	-3.039865000	-4.361555000	0.430707000
H	-3.106579000	-5.095933000	1.235801000
C	-2.888468000	-4.802911000	-0.883532000
H	-2.834079000	-5.872782000	-1.099771000
C	-2.825391000	-3.871883000	-1.922134000
H	-2.734897000	-4.223068000	-2.952710000
C	-2.892278000	-2.496343000	-1.661803000
C	-3.697567000	1.920895000	0.400631000
C	-3.674695000	2.822525000	-0.693981000
C	-3.189199000	4.131437000	-0.530283000
H	-3.155053000	4.795878000	-1.396409000
C	-2.717996000	4.592282000	0.706776000
C	-2.780199000	3.710465000	1.795571000
H	-2.422022000	4.057851000	2.769946000
C	-3.249737000	2.394424000	1.660756000
C	-2.823703000	-1.493657000	-2.804789000
H	-3.173983000	-0.537528000	-2.396458000
C	-3.221290000	-2.527769000	2.176704000
H	-3.662498000	-1.520970000	2.148189000
C	-4.067398000	2.349272000	-2.090545000
H	-4.522098000	1.353167000	-1.984502000
C	-2.044071000	5.947560000	0.867208000
H	-2.168437000	6.245210000	1.923141000
C	-3.205066000	1.470781000	2.872398000
H	-3.644091000	0.513549000	2.553178000
C	-5.505962000	-0.158895000	-0.094371000
N	-5.731507000	-0.974088000	-1.066223000

C	-7.867688000	-0.530229000	-0.055967000
H	-8.456146000	0.230385000	-0.592239000
H	-8.569707000	-1.093119000	0.577800000
C	-7.129253000	-1.438780000	-1.076246000
C	-6.982130000	1.665525000	0.911663000
H	-7.939254000	1.856752000	1.425134000
H	-6.181393000	2.148123000	1.489399000
C	-6.586052000	-0.476921000	2.160942000
H	-7.483205000	-0.294823000	2.775176000
H	-5.723633000	-0.049948000	2.693604000
H	-6.433479000	-1.563870000	2.095559000
C	-7.118486000	-2.917444000	-0.655291000
H	-8.133015000	-3.345956000	-0.696504000
H	-6.734465000	-3.030710000	0.369516000
H	-6.460309000	-3.496532000	-1.320444000
C	-7.696460000	-1.303079000	-2.492199000
H	-8.756402000	-1.603728000	-2.520557000
H	-7.138301000	-1.936638000	-3.198500000
O	1.630356000	0.186745000	0.066182000
C	2.863567000	0.569526000	-0.024730000
B	4.044935000	-0.388400000	0.136578000
K	0.260875000	-2.021845000	-0.116430000
C	3.002395000	2.051317000	-0.321970000
C	3.103876000	2.991520000	0.730446000
C	3.039614000	4.361711000	0.431424000
H	3.106147000	5.096028000	1.236588000
C	2.888355000	4.803164000	-0.882796000
H	2.833909000	5.873047000	-1.098956000
C	2.825494000	3.872214000	-1.921481000
H	2.735109000	4.223479000	-2.952037000
C	2.892467000	2.496659000	-1.661255000
C	3.697582000	-1.920798000	0.400791000
C	3.674672000	-2.822521000	-0.693756000
C	3.189092000	-4.131386000	-0.529969000

H	3.154891000	-4.795879000	-1.396053000
C	2.717856000	-4.592123000	0.707119000
C	2.780126000	-3.710240000	1.795851000
H	2.421918000	-4.057532000	2.770248000
C	3.249736000	-2.394232000	1.660946000
C	2.824065000	1.494048000	-2.804327000
H	3.175002000	0.538080000	-2.396175000
C	3.220934000	2.527825000	2.177309000
H	3.662165000	1.521037000	2.148804000
C	4.067254000	-2.349359000	-2.090388000
H	4.522346000	-1.353428000	-1.984412000
C	2.043830000	-5.947343000	0.867604000
H	2.167883000	-6.244808000	1.923626000
C	3.205200000	-1.470579000	2.872584000
H	3.644106000	-0.513322000	2.553282000
C	5.506047000	0.158799000	-0.094554000
N	5.731543000	0.974072000	-1.066351000
C	7.867904000	0.529376000	-0.056819000
H	8.455937000	-0.231375000	-0.593363000
H	8.570308000	1.091956000	0.576794000
C	6.746952000	-0.155935000	0.765915000
C	7.129446000	1.438290000	-1.076767000
C	6.981980000	-1.666147000	0.910958000
H	7.939356000	-1.857669000	1.423842000
H	7.018652000	-2.157301000	-0.074069000
H	6.181454000	-2.148499000	1.489201000
C	6.586904000	0.476348000	2.160435000
H	7.483921000	0.293371000	2.774606000
H	5.724117000	0.050104000	2.693088000
H	6.435370000	1.563443000	2.095154000
C	7.119368000	2.916922000	-0.655697000
H	8.134061000	3.345027000	-0.697113000
H	6.735625000	3.030268000	0.369206000
H	6.461287000	3.496332000	-1.320664000

C	7.696142000	1.302484000	-2.492918000
H	8.756172000	1.602790000	-2.521610000
H	7.137953000	1.936264000	-3.198999000
H	7.621893000	0.259476000	-2.838783000
C	-6.746707000	0.155371000	0.766489000
H	-7.019580000	2.156630000	-0.073358000
H	-7.622667000	-0.260019000	-2.838035000
H	-2.469108000	6.719303000	0.260037000
H	-0.993932000	5.852829000	0.685180000
H	-4.795405000	2.979741000	-2.556823000
H	-3.198597000	2.233109000	-2.704216000
H	-3.865508000	-3.141749000	2.770767000
H	-2.254427000	-2.438057000	2.626178000
H	-3.483230000	-1.741233000	-3.610165000
H	-1.817969000	-1.355003000	-3.142687000
H	-3.789103000	1.839029000	3.689830000
H	-2.197578000	1.267440000	3.169923000
H	1.818290000	1.354904000	-3.141899000
H	3.483175000	1.742027000	-3.609920000
H	2.254004000	2.438101000	2.626637000
H	3.865067000	3.141786000	2.771485000
H	3.198323000	-2.232852000	-2.703809000
H	4.794886000	-2.980072000	-2.556919000
H	0.993745000	-5.852611000	0.685263000
H	2.469023000	-6.719206000	0.260694000
H	2.197756000	-1.267313000	3.170308000
H	3.789428000	-1.838800000	3.689891000

References

[1] M.-A. Légaré, M. Rang, G. Bélanger-Chabot, J. I. Schweizer, I. Krummenacher, R. Bertermann, M. Arrowsmith, M. C. Holthausen, H. Braunschweig, *Science* **2019**, *363*, 1329–1332.

[2] S. Stoll, A. Schweiger, *J. Magn. Reson.* **2006**, *178*, 42–55.

[3] G. M. Sheldrick, *Acta Cryst.* **2015**, *A71*, 3–8.

[4] G. M. Sheldrick, *Acta Cryst.* **2008**, *A64*, 112–122.

[5] A. L. Spek, *Acta Cryst.* **2015**, *C71*, 9–18.

[6] S. H.Vosko, L. Wilk, M. Nusair, *Can. J. Phys.* **1980**, *58*, 1200–1211.

[7] C. Lee, W. Yang, R. G. Parr, *Phys. Rev. B* **1988**, *37*, 785–789.

[8] A. D. Becke, *J. Chem. Phys.* **1993**, *98*, 5648–5652.

[9] P. J. Stephens, F. J. Devlin, C. F. Chabalowski, M. J. Frisch, *J. Phys. Chem.* **1994**, *98*, 11623–11627.

[10] S. Grimme, J. Antony, S. Ehrlich, H. A. Krieg, *J. Chem. Phys.* **2010**, *132*, 154104.

[11] S. Grimme, S. Ehrlich, L. Goerigk, *J. Comput. Chem.* **2011**, *32*, 1456–1465.

[12] F. Weigend, R. Ahlrichs, *Phys. Chem. Chem. Phys.* **2005**, *7*, 3297.

[13] R. Ditchfield, W. J. Hehre, J. A. Pople, *J. Chem. Phys.* **1971**, *54*, 724–728.

[14] W. J. Hehre, R. Ditchfield, J. A. Pople, *J. Chem. Phys.* **1972**, *56*, 2257–2261.

[15] P. C. Hariharan, J. A. Pople, *Theor. Chim. Acta* **1973**, *28*, 213–222.

[16] J. D. Dill, J. A. Pople, *J. Chem. Phys.* **1975**, *62*, 2921–2923.

[17] T. Clark, J. Chandrasekhar, G. W. Spitznagel, P. v. R. Schleyer, *J. Comput. Chem.* **1983**, *4*, 294–301.

[18] A. V. Marenich, C. J. Cramer, D. G. Truhlar, *J. Phys. Chem. B* **2009**, *113*, 6378–6396.

[19] R. Seeger, J. A. Pople, *J. Chem. Phys.* **1977**, *66*, 3045–3050.

[20] R. Bauernschmitt, R. Ahlrichs, *J. Chem. Phys.* **1996**, *104*, 9047–9052.

[21] R. S. Mulliken, *J. Chem. Phys.* **1955**, *23*, 1833–1840.

[22] J. A. Cioslowski, *J. Am. Chem. Soc.* **1989**, *111*, 8333–8336.

[23] I. Mayer, *Chem. Phys. Lett.* **1983**, *97*, 270–274.

[24] I. Mayer, *Int. J. Quantum Chem.* **1984**, *26*, 151–154.

[25] B. O. Roos, In *Advances in Chemical Physics: Ab Initio Methods in Quantum Chemistry Part 2, e*d.: K. P. Lawley, John Wiley & Sons, **1987**, *69*, 399–445.

[26] C. Angeli, R. Cimiraglia, S. Evangelisti, T. Leininger, J.-P. Malrieu, *J. Chem. Phys.* **2001**, *114*, 10252–10264.

[27] C. Angeli, R. Cimiraglia, J.-P. Malrieu, *Chem. Phys. Lett.* **2001**, *350*, 297–305.

[28] C. Angeli, R. Cimiraglia, J.-P. Malrieu, *J. Chem. Phys.* **2002**, *117*, 9138–9153.

[29] F. Neese, *J. Comput. Chem.* **2003**, *24*, 1740–1747.

[30] F. Neese, F. Wennmohs, A. Hansen, U. Becker, *Chem. Phys.* **2009**, *356*, 98–109.

[31] K. Yamaguchi, *Chem. Phys. Lett.* **1975**, *33*, 330–335.

[32] S. Yamanaka, M. Okumura, M. Nakano, K. Yamaguchi, *J. Mol. Struct.* **1994**, *310*, 205–218.

[33] M. Nakano, *Top. Curr. Chem.* **2017**, *375*, 47.

[34] F. Weinhold, C. R. Landis, E. D. Glendening, *Int. Rev. Phys. Chem.* **2016**, *35*, 399–440.

[35] J. E. Carpenter, F. Weinhold, *J. Mol. Struct.* **1988**, *169*, 41–62.

[36] E. D. Glendening, C. R. Landis, F. Weinhold, *J. Comput. Chem.* **2019**, jcc.25873.

[37] F. Neese, *Wiley Interdiscip. Rev. Comput. Mol. Sci.* **2012**, *2*, 73–78.

[38] T. Lu, F. Chen, *J. Comput. Chem.* **2012**, *33*, 580–592.

[39] M. J. Frisch, G. W. Trucks, H. B. Schlegel, G. E. Scuseria, M. A. Robb, J. R. Cheeseman, G. Scalmani, V. Barone, G. A. Petersson, H. Nakatsuji, X. Li, M. Caricato, A. V. Marenich, J. Bloino, B. G. Janesko, R. Gomperts, B. Mennucci, H. P. Hratchian, J. V. Ortiz, A. F. Izmaylov, J. L. Sonnenberg, D. Williams-Young, F. Ding, F. Lipparini, F. Egidi, J. Goings, B. Peng, A. Petrone, T. Henderson, D. Ranasinghe, V. G. Zakrzewski, J. Gao, N. Rega, G. Zheng, W. Liang, M. Hada, M. Ehara, K. Toyota, R. Fukuda, J. Hasegawa, M. Ishida, T. Nakajima, Y. Honda, O. Kitao, H. Nakai, T. Vreven, K. Throssell, J. A. Montgomery, Jr., J. E. Peralta, F. Ogliaro, M. J. Bearpark, J. J. Heyd, E. N. Brothers, K. N. Kudin, V. N. Staroverov, T. A. Keith, R. Kobayashi, J. Normand, K. Raghavachari, A. P. Rendell, J. C. Burant, S. S. Iyengar, J. Tomasi, M. Cossi, J. M. Millam, M. Klene, C. Adamo, R. Cammi, J. W. Ochterski, R. L. Martin, K. Morokuma, O. Farkas, J. B. Foresman, D. J. Fox, Gaussian 16, Revision B.01. Gaussian, Inc., Wallingford CT, **2016**.

[40] V. Bonačić-Koutecký, J. Koutecký, J. Michl, *Angew. Chemie Int. Ed.* **1987**, *26*, 170–189.

[41] J. Michl, V. Bonačić-Koutecký, *Tetrahedron* **1988**, *44*, 7559–7585.
